# Age-Dependent Brain Gene Expression and Copy Number Anomalies in Autism Suggest Distinct Pathological Processes at Young Versus Mature Ages

**DOI:** 10.1371/journal.pgen.1002592

**Published:** 2012-03-22

**Authors:** Maggie L. Chow, Tiziano Pramparo, Mary E. Winn, Cynthia Carter Barnes, Hai-Ri Li, Lauren Weiss, Jian-Bing Fan, Sarah Murray, Craig April, Haim Belinson, Xiang-Dong Fu, Anthony Wynshaw-Boris, Nicholas J. Schork, Eric Courchesne

**Affiliations:** 1 Department of Neuroscience, NIH–UCSD Autism Center of Excellence, School of Medicine, University of California San Diego, La Jolla, California, United States of America; 2 Division of Medical Genetics, Department of Pediatrics and Institute of Human Genetics, School of Medicine, University of California San Francisco, San Francisco, California, United States of America; 3 Scripps Genomic Medicine and The Scripps Translational Sciences Institute (STSI), La Jolla, California, United States of America; 4 Graduate Program in Biomedical Sciences, Department of Medicine, University of California San Diego, La Jolla, California, United States of America; 5 Department of Cellular and Molecular Medicine, School of Medicine, University of California San Diego, La Jolla, California, United States of America; 6 Department of Psychiatry, School of Medicine, University of California San Francisco, San Francisco, California, United States of America; 7 Illumina, San Diego, California, United States of America; Georgia Institute of Technology, United States of America

## Abstract

Autism is a highly heritable neurodevelopmental disorder, yet the genetic underpinnings of the disorder are largely unknown. Aberrant brain overgrowth is a well-replicated observation in the autism literature; but association, linkage, and expression studies have not identified genetic factors that explain this trajectory. Few studies have had sufficient statistical power to investigate whole-genome gene expression and genotypic variation in the autistic brain, especially in regions that display the greatest growth abnormality. Previous functional genomic studies have identified possible alterations in transcript levels of genes related to neurodevelopment and immune function. Thus, there is a need for genetic studies involving key brain regions to replicate these findings and solidify the role of particular functional pathways in autism pathogenesis. We therefore sought to identify abnormal brain gene expression patterns via whole-genome analysis of mRNA levels and copy number variations (CNVs) in autistic and control postmortem brain samples. We focused on prefrontal cortex tissue where excess neuron numbers and cortical overgrowth are pronounced in the majority of autism cases. We found evidence for dysregulation in pathways governing cell number, cortical patterning, and differentiation in young autistic prefrontal cortex. In contrast, adult autistic prefrontal cortex showed dysregulation of signaling and repair pathways. Genes regulating cell cycle also exhibited autism-specific CNVs in DNA derived from prefrontal cortex, and these genes were significantly associated with autism in genome-wide association study datasets. Our results suggest that CNVs and age-dependent gene expression changes in autism may reflect distinct pathological processes in the developing versus the mature autistic prefrontal cortex. Our results raise the hypothesis that genetic dysregulation in the developing brain leads to abnormal regional patterning, excess prefrontal neurons, cortical overgrowth, and neural dysfunction in autism.

## Author Summary

Autism is a disorder characterized by aberrant social, communication, and restricted and repetitive behaviors. It develops clinically in the first years of life. Toddlers and children with autism often exhibit early brain enlargement and excess neuron numbers in the prefrontal cortex. Adults with autism generally do not display enlargement but instead may have a smaller brain size. Thus, we investigated DNA and mRNA patterns in prefrontal cortex from young versus adult postmortem individuals with autism to identify age-related gene expression differences as well as possible genetic correlates of abnormal brain enlargement, excess neuron numbers, and abnormal functioning in this disorder. We found abnormalities in genetic pathways governing cell number, neurodevelopment, and cortical lateralization in autism. We also found that the key pathways associated with autism are different between younger and older autistic individuals. These findings suggest that dysregulated gene pathways in the early stages of neurodevelopment could lead to later behavioral and cognitive deficits associated with autism.

## Introduction

Clinical and subclinical manifestations of autism begin during the first years of life [1]. Neuroimaging studies of living infants and young children with autism have revealed abnormal brain overgrowth in the majority of cases, particularly in prefrontal, temporal and amygdala regions [2]–[13]. These studies have also shown abnormal functional asymmetry and activation in the cortex and cerebellum [14]–[16]. Children with autism have 67% excess neuron numbers in the prefrontal cortex, a substantial pathology that points to disruption of early developmental mechanisms that govern neuron numbers, and an 18% increase in brain weight at autopsy [17]. However, by late adolescence and early adulthood, the autistic brain commonly displays neuron loss and cortical thinning and is no longer enlarged [2], [8], [13], [18]. Therefore, the underlying developmental molecular defects in the majority of autistic cases seem likely to be most evident at younger rather than older ages.

The average age of individuals studied in more than fifty postmortem analyses of the autistic brain is 22 years [19], making it difficult if not impossible to comprehensively assess early developmental molecular pathologies associated with autism. In studies combining both young and adult autistic brain tissue, transcript and protein expression patterns suggest that cortical patterning, synaptic [20], apoptotic [21], immune [20], [22], and inflammatory [23] aberrances in addition to dysregulation of neurotransmitter systems [24] may be involved in autism. None of these molecular pathologies at older ages, however, can explain the 67% excess prefrontal neuron numbers and brain enlargement at younger ages in autism.

Thus, there seems to be a gap between neuroanatomical and cellular abnormalities reported for autism at younger ages and molecular pathologies in autism at older ages. Molecular pathologies specifically present at younger ages in autism are largely unknown and unexplored. Also, whether some molecular abnormalities detected in the older autistic brain reflect conditions unique to the older brain and whether others are common to both younger and older autistic brains are unknown. Genome-wide analyses of the genes, pathways and processes that exhibit dysregulation specifically in the young autistic brain have not been pursued to a great degree. This is particularly true for the prefrontal and temporal cortex, where abnormalities of growth and function during early development are pronounced and likely to contribute significantly to social, communication, language and emotional deficits associated with autism. Additionally, although copy number variation (CNV) has been hypothesized to play an important role in the pathogenesis of autism [25], [26] and may underlie important differences in gene expression, CNVs identified in brain tissue from individuals with autism have not been examined in relation to aberrant brain gene expression from the same tissue samples.

We examined the linked hypotheses that underlying neuroanatomical differences between younger and adult individuals with autism are a result of differences in molecular pathology. We hypothesize that some molecular pathologies at younger ages in autism may provide insight into the very early neural developmental processes that lead to the disorder. To do so, we identified abnormal genetic pathways in the young autistic brain, determined expression patterns that distinguished the young from the adult autistic brain and identified evidence for gene expression dysregulation that is age-independent using genome-wide expression and genotyping techniques. We found age-dependent gene expression differences between young and adult autistic brains as well as rare and common genetic variants associated with autism that have important roles in neurodevelopment.

## Results

First, we analyzed genome-wide expression of 57 frozen samples of dorsolateral prefrontal cortex (DLPFC, BA 9/46; Table S1) using a DASL-based platform on the Illumina Human-Ref8 v3 microarray [27], [28]. Thirty-three samples in total passed quality control measures (Text S1): 16 of these samples were from young postmortem males (2–14 y; autism = 9, control = 7) and 17 were from adult males (15–56 y, autism = 6, control = 11). Though RNA Integrity Numbers (RIN) are not predictive of array quality [29], multiple RIN assessments were used to determine RNA quality (Table S1). We found no significant differences between the RINs of the autism and control groups (t = 0.16, DF = 20, p = 0.87).

Two-way analysis of variance (ANOVA) and post-hoc pair-wise ANOVA-based comparisons were performed to identify genes exhibiting expression differences in young autistic vs. young control DLPFC compared to adult autistic vs. adult control DLPFC. An overall ANOVA-based F-test p-value<0.05 [empirical false discovery rate (FDR), not threshold, FDR = 0.27] was used to identify genes showing an effect of interaction between age and diagnosis for further analyses. From the two-way ANOVA results, we attempted to tease out expression differences between: 1) young autism, 2) young control, 3) adult autism and 4) adult control groups.

Of the genes exhibiting an effect of interaction from ANOVA-based F-tests with p-values<0.05, 102 genes resulted in ANOVA-based post-hoc contrasts between young autistic and control groups with p-value<0.05 (Figure S1). Seven hundred thirty six genes resulted in an ANOVA-based post-hoc contrast between adult autistic and control cases (p-value<0.05). Finally, an overall ANOVA-based F-test p-value<0.05 (empirical FDR = 0.13) was performed to assess the main effect of diagnosis across all autistic and control cases independent of age. Two thousand seventeen genes resulted in this contrast with p-value<0.05. Genes common between the three comparisons are shown in Figure S1.

Genes exhibiting a diagnosis main effect and a post-hoc pairwise contrast (e.g., autism vs. control, adult vs. young; p-value<0.05) were further subjected to enrichment analyses in the MetaCore software suite (MetaCore from GeneGo Inc.) as well as the online enrichment program DAVID [30], [31]. By focusing on genes exhibiting a diagnosis main effect, we explored pathological mRNA expression in autism irrespective of age. qPCR validation verified expression differences on a subset of the differentially expressed genes (Figure S2).

### Genes regulating cell number, genetic integrity, and neural patterning are dysregulated in young autistic cases

Since early brain overgrowth and the clinical onset of autistic symptoms occur at young ages in autism, we focused on unique gene expression differences between autistic and control cases below the age of 14 years to identify genes that may be dysregulated early in autism pathogenesis. One hundred two genes were differentially expressed in young autistic cases compared with the young control group and exhibited a diagnosis x age group interaction effect (p-value<0.05, empirical FDR = 0.27; Table S2). Many of these genes were previously identified autism candidate loci based on the literature (Table S3).

Pathway enrichment analyses of these 102 genes via the MetaCore software suite (defining an enriched pathway as having an enrichment p-value<0.05 and empirical FDR<0.1) suggested that DNA damage-response, cell cycle and apoptosis-related MetaCore pathways (‘M-Pathways’) were significantly altered (Table 1, Young autism versus young control map folders). Key players such as BRCA1 and CHK2 were downregulated. Most significantly dysregulated M-Pathways in this category included the DNA-damage-induced response and role of NFBD1 in DNA damage response. A Development-Neurogenesis Process Network, Development-Neurogenesis (p = 1.09E-03, 6/192 network objects), was also significantly altered.

**Table 1:** MetaCore Pathway Map Folders (left) and M-Pathways (right) in three ANOVA-based analyses.

Young autism versus young control map folders	P-value	Ratio	Young autism vs. young control pathway name	P-value	Network objects
No significant map folders			DNA damage_DNA-damage-induced responses	0.0004634	2/9
			Immune response_NF-AT signaling and leukocyte interactions	0.0006138	3/46
			DNA damage_Role of NFBD1 in DNA damage response	0.000995	2/13
			Apoptosis and survival_DNA-damage-induced apoptosis	0.001333	2/15
			DNA damage_ATM/ATR regulation of G2/M checkpoint	0.004024	2/26
			DNA damage_Brca1 as a transcription regulator	0.005337	2/30
			DNA damage_Role of Brca1 and Brca2 in DNA repair	0.005337	2/30
			DNA damage_ATM/ATR regulation of G1/S checkpoint	0.006058	2/32
			Cell cycle_Regulation of G1/S transition (part 1)	0.008469	2/38
			Translation _Regulation of EIF2 activity	0.008907	2/39

Adult autism versus adult control map folders	P-value	Ratio	Adult autism vs. adult control pathway name	P-value	Network objects
Cell differentiation	0.000008641	37/920	Development_Role of Activin A in cell differentiation and proliferation	0.00006435	6/40
Mitogenic signaling	0.0001335	24/548	Signal transduction_PKA signaling	0.0002574	6/51
Apoptosis	0.0001475	31/809	Development_MAG-dependent inhibition of neurite outgrowth	0.0004451	5/37
Tissue remodeling and wound repair	0.0007248	21/504	Cell cycle_Regulation of G1/S transition (part 1)	0.0005053	5/38
Cystic fibrosis disease	0.00727	22/649	Development_A2A receptor signaling	0.0009027	5/43
Vasoconstriction	0.007939	13/312	Neurophysiological process_PGE2-induced pain processing	0.0009027	5/43
DNA-damage response	0.008795	13/316	ENaC regulation in airways (normal and CF)	0.002146	5/52
Immune system response	0.009623	28/914	Apoptosis and survival_Endoplasmic reticulum stress response pathway	0.002336	5/53
Vasodilation	0.009723	13/320	Development_Activation of astroglial cells proliferation by ACM3	0.002572	4/33
Neurotransmission	0.01026	22/669	Signal transduction_Activin A signaling regulation	0.002572	4/33
Vascular development (Angiogenesis)	0.01195	17/479	Oxidative stress_Role of ASK1 under oxidative stress	0.002876	4/34
Cardiac Hypertrophy	0.01211	9/190	Apoptosis and survival_Role of CDK5 in neuronal death and survival	0.002876	4/34
Protein degradation	0.01665	11/270	Fatty Acid Omega Oxidation	0.002876	4/34
Estrogen signaling	0.02384	11/285	Development_Role of CDK5 in neuronal development	0.002876	4/34
Inflammatory response	0.03608	18/585			
Protein synthesis	0.03667	11/305			
Calcium signaling	0.03896	14/426			
Blood clotting	0.04603	10/278			

Diagnosis main effect map folders	P-value	Ratio	Diagnosis main effect pathway name	P-value	Network objects
DNA-damage response	2.98E-07	32/316	Development_A2A receptor signaling	1.704E-07	11/43
Apoptosis	8.41E-07	58/809	Development_A2B receptor: action via G-protein alpha s	0.000007049	10/50
Immune system response	4.62E-06	61/914	Transcription_P53 signaling pathway	0.00004903	8/39
Tissue remodeling and wound repair	5.48E-06	40/504	Transcription_CREB pathway	0.0001213	8/44
Cell differentiation	5.73E-06	61/920	Development_Thrombopoietin-regulated cell processes	0.0001431	8/45
Vascular development (Angiogenesis)	2.38E-05	37/479	Development_PIP3 signaling in cardiac myocytes	0.0001965	8/47
Cardiac Hypertrophy	3.28E-05	20/190	Cell adhesion_ECM remodeling	0.000404	8/52
Inflammatory response	3.69E-05	42/585	Cell cycle_Role of 14-3-3 proteins in cell cycle regulation	0.00082	5/22
Cell cycle and its regulation	8.89E-05	32/414	Cytoskeleton remodeling_Cytoskeleton remodeling	0.000841	11/102
Mitogenic signaling	1.90E-04	38/548	Reproduction_GnRH signaling	0.0008578	9/72
Vasodilation	1.93E-04	26/320	Development_Role of IL-8 in angiogenesis	0.000858	8/58
Hematopoiesis	2.07E-04	23/268	Immune response_MIF - the neuroendocrine-macrophage connector	0.0009946	7/46
Calcium signaling	3.43E-04	31/426	Signal transduction_PTEN pathway	0.0009946	7/46
Vasoconstriction	3.77E-03	22/312	Development_HGF signaling pathway	0.001134	7/47
Myogenesis regulation	3.48E-02	8/99	Development_A3 receptor signaling	0.00146	7/49
Blood clotting	3.64E-02	17/278	Development_GM-CSF signaling	0.001647	7/50
Protein synthesis	4.29E-02	18/305	Cytoskeleton remodeling_TGF, WNT and cytoskeletal remodeling	0.001692	11/111
			Development_IGF-1 receptor signaling	0.001853	7/51
			Signal transduction_cAMP signaling	0.001878	6/38
			Cell adhesion_PLAU signaling	0.002156	6/39
			DNA damage_Role of SUMO in p53 regulation	0.002442	4/17
			Cell adhesion_Chemokines and adhesion	0.00254	10/100
			Proteolysis_Putative SUMO-1 pathway	0.003026	5/29
			Apoptosis and survival_BAD phosphorylation	0.003175	6/42
			Development_Osteopontin signaling in osteoclasts	0.003529	5/30

(A–C) MetaCore Map Folders and M-Pathways identified in posthoc young autism vs. young control analysis (p<0.05, FDR = 0.1; A), posthoc adult autism vs. adult control analysis (p<0.05, FDR = 0.1; B) and diagnosis main effect analyses comparing all autism and all control cases (Top 25; C). P-values and numbers of network objects/ratios of differentially expressed genes are shown.

**Figure 1:**
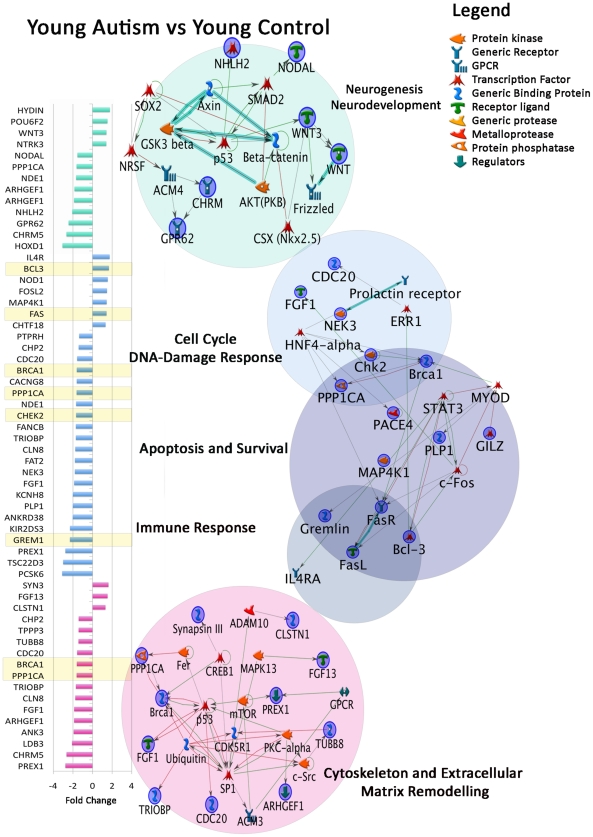
Dysregulated gene expression in developmental M-Pathways and Process Networks in young autistic prefrontal cortex. From differentially expressed genes of young autistic vs. young control cases (Table S2), networks were created using MetaCore Network Analysis. Colors on fold change graph correspond to circles on the right depicting network maps in each category. Yellow = differentially expressed genes in two or more functional domains. Overlapping circles = differentially expressed genes common to two domains.

We additionally annotated all 102 genes using DAVID and identified overlapping sets of 12, 19, 7 and 16 genes involved in DNA damage/cell cycle, apoptosis, immune signaling and neurogenesis and neural development (Figure 1), respectively. Of the 7 immune response genes, 4 (FAS, BCL3, GREM1 and FOSL2) were also found to contribute to apoptosis. Neurogenesis and neural development pathways included the WNT pathway and were driven by dysregulation of the WNT3 gene. WNT pathway genes are known to regulate cell proliferation, cell fate and patterning during embryogenesis [32]. Downstream components of the WNT pathway, such as Dvl1, are also known to regulate social behavior in mouse models [33]. In addition, we found significant downregulation of genes involved in neural patterning and differentiation, such as FGF1, HOXD1, NDE1, NODAL, PCSK6 and GREM1 (Figure 1, Table S2).

Expression anomalies of genes involved in DNA damage, cell cycle and apoptosis may contribute to abnormal brain growth by increasing production or reducing elimination of neurons during development. Dysregulated neural patterning and differentiation genes may lead to abnormal cellular organization and cytoarchitecture. For example, HOX and DLX family genes play important roles in vertebrate patterning and are important for neuronal subtype differentiation [34]. Moreover, NODAL controls dorsal mesoderm induction, anterior patterning and initiation of left-right asymmetry during gastrulation [35]. These findings suggest that in the young autistic brain, genetic regulation of cell number, genetic integrity and neural patterning is disturbed.

### Genes regulating signaling, repair, and response pathways are dysregulated in adult autistic cases

**Figure 2:**
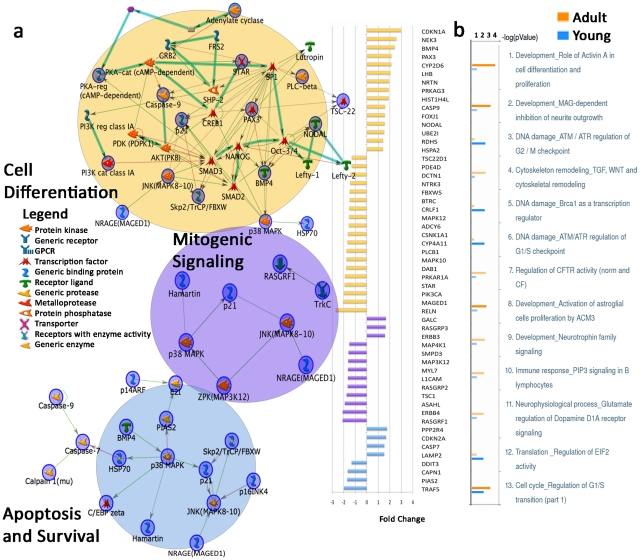
Dysregulated gene expression in top three Map Folders in adult autistic prefrontal cortex and differentially affected M-Pathways of young and adult autistic cases. (A) Graph on the right shows fold change of genes in cell differentiation, mitogenic signaling, and apoptosis and survival Map Folders. Colors on fold change graph correspond to circles on the left depicting network maps in each category. From each category of differentially expressed genes in adult autistic vs. adult control posthoc comparison (Table S4), gene networks on the left were created using MetaCore Network Analysis. (B) Top differentially affected M-pathway comparisons of dysregulated genes between adult and young autistic cases. Bars represent significance of listed pathways. Orange = adult; blue = young; faded color = FDR>0.1 or p>0.05.

As a complement to the analyses of the young autistic brain, we compared the genes dysregulated in young autistic cases to those dysregulated in adult autistic cases. We identified genes using the same ANOVA diagnosis x age interaction effect p-value criterion but with emphasis on genes differentially expressed in adult autistic brains relative to adult control brains (defined as cases ≥15 years of age). Seven hundred thirty six genes were differentially expressed based on this analysis (Figure S1, Table S4). These genes were also analyzed with MetaCore for functional enrichment. The 3 most significant MetaCore Map Folders (‘Map Folders’: a label MetaCore provides to sets of genes with an overarching function of pathway participation) included cell differentiation, mitogenic signaling and apoptosis genes (Table 1, Adult autism versus adult control map folders, Figure 2A; p<0.05, FDR<0.1). Dysregulated pathways specific for the adult brain included multiple signaling and remodeling functions in neurons and glia (Table 1, Adult autism versus adult control map folders). M-Pathway categories that were dysregulated in adults but not young cases included development, signaling and oxidative stress pathways (Table 1, Figure 2B).

The Cell Differentiation Map Folder included significantly dysregulated genes RELN, BTRC, BMP4, MAPK10 and NTRK3 (Figure 2A). This Map Folder also included suggested dysregulation of the ‘Activin A in Cell Differentiation and Proliferation’ pathway, which involved the genes LHB, NODAL, STAR, CDKN1A, PRKAR1A and ADCY6. Genes playing multiple functions in all three top map folders included MAPK12, CDKN1A, NTRK3, PRKAR1A, PIK3CA, CASP9, MAPK10, ADCY6 and MAGED1 (Figure 2A). Notably, Tissue Remodeling and Wound Repair-related genes also exhibited dysregulation in adult autistic cases (Table 1, Adult autism versus adult control map folders).

These analyses suggest that in the adult autistic brain, cell differentiation, mitogenic, apoptotic and remodeling and repair functions could be components of recovery responses, in accordance with previous reports [22], [23], [36]. They could, however, also be signatures of ongoing reparatory neurogenesis processes [37]. For example, the Activin A signaling pathway is expressed by neurons following injury and is essential for adult neurogenesis [38], [39]. BTRC inhibits the beta-catenin (CTNNB1) pathway [40], which in adult animals is upregulated after insults such as seizures [41] and promotes adult neurogenesis [42]. Furthermore, BMP4 expressed in adult subventricular zones serves to inhibit neurogenesis [43]. Thus, it appears that aberrant signaling and repair processes may distinguish adult autistic cases from young cases.

### Genes important in development were dysregulated across both young and adult autistic cases

**Figure 3:**
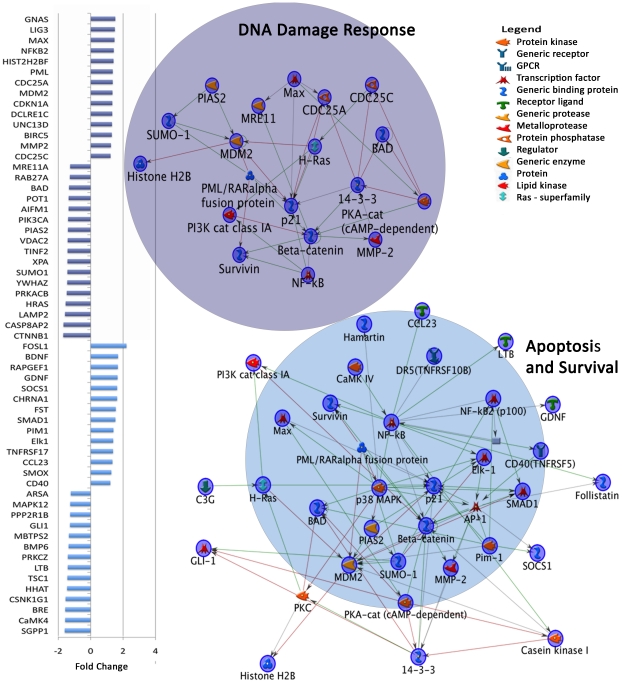
Dysregulated gene expression in top two MetaCore Map Folders in autism independent of age. Graph on left shows fold change of genes in DNA damage response and apoptosis and survival Map Folders. The third most significant map folder is depicted in Figure S3. Colors on fold change graph correspond to circles on the left depicting network maps in each category. From each category of differentially expressed genes in the all autistic vs. all control comparison (Table S5), networks were created using MetaCore Network Analysis.

Finally, we examined genes showing a main effect of diagnosis to identify differentially expressed between autistic and control cases independent of age (i.e., we did not confine attention to genes exhibiting diagnosis x age interaction effects or age-specific effects). More than 2000 such genes were detected based on a simple contrast between autism and control brains (p<0.05, empirical FDR = 0.13; Figure S1, Table S5). Enrichment analyses of these genes using MetaCore suggested that seventeen Map Folders were altered in all autistic brains (p<0.05, FDR = 0.1; Table 1, Diagnosis main effect map folders). The three most significant Map Folders included DNA-damage response, apoptosis and immune system response functions (Figure 3, Figure S3). In addition, the top 25 M-Pathways (Table 1, Diagnosis main effect map folders, Table S6) included cell cycle [14-3-3 (YWHAZ), CDC25A, CDCD25C, ATRX], proliferation [CTNNB1 (beta-catenin), FSHB, PRKACB, PRKCZ], apoptosis (BAD, CASP8, CASP10, MDM2), cytoskeleton and extracellular matrix remodeling (ErbB4, MMP2, NID1, TIMP1, COL4A3) and growth and development [RELN, ROBO1, ADORA2A, p21 (CDKN1A), 14-3-3, HGF, FGFRL1, TSC1] functions. Specifically, the p53 signaling pathway and the PTEN pathway were among these dysregulated M-Pathways (Table 1, Diagnosis main effect map folders).

A number of genes in these M-Pathways have known functions in neuronal development: PTEN signaling regulates proliferation [44] and is associated with macrocephaly in autism [45], [46]. TSC1 is mutated in tuberous sclerosis, acts downstream of PTEN in the mTOR pathway [47] and affects cortical lamination, neuronal migration and axon pathfinding [48]. CTNNB1, a key member of the WNT pathway, regulates cerebral cortical size. CTNNB1 transgenic mice have enlarged and folded cortices [49]. Furthermore, RELN is critical for human neuronal migration [50] and has been previously linked to autism [51]. Genes differentiating autistic cases from controls independent of age have important developmental, immune and cytoskeletal remodeling functions.

Finally, given the novel cytoskeletal age-independent M-Pathways identified in this analysis, we revisited the 102 significant genes identified in the young autism vs. control analysis. We found 17 cytoskeletal and matrix remodeling genes (Figure 1). These analyses support a role for cytoskeletal dysregulation in young autistic brains as well. Cytoskeletal elements have been linked to defects in neuronal migration in other neurodevelopmental disorders such as lissencephaly [52].

### Comparison with a published functional genomic study shows overlap in repair and immune response genes

Due to commonalities between previously identified candidate loci and the genes we found to exhibit expression differences between young autism vs. control, we also investigated similarities between the genes we identified and those identified in a recent functional genomic study. We compared genes showing a main effect of diagnosis in the present study with differentially expressed genes implicated recently by Voineagu et al. [20].

Twenty-five probes detected as p<0.05 in comparing autism and control cases and 21,564 probes detected as p>0.05 in both studies were identified as overlapping (p<0.0001). Among the probes detected at p<0.05 were 6 genes important for Tissue Remodeling and Wound Repair (p = 1.622E-4, FDR<0.05) and 7 genes important for Immune System Response (p = 4.772E-4, FDR<0.05). Ultimately, we found some consistency between genes detected in our analyses with those of a previous functional genomic study, particularly within domains of repair and immune response.

### Genetic variation may underlie cell cycle and cytoskeleton gene dysregulation

To further investigate the findings of dysregulated genetic functions in young autistic cases particularly, we determined whether CNVs in autistic cases were distinct from those in controls. We genotyped prefrontal cortex samples from 55 of the 57 total autistic and control cases in our study. After quality control, CNV enrichment was analyzed in 30 DLPFC cases from male and female autistic and control cases (Table S1) using PennCNV with GC adjustment and CNVision programs [53], [54]. Nearly all (>99%) brain-derived CNVs in autistic and control cases were deletions (Table S7, Table S8). There were no differences between autistic and control cases in numbers of CNVs, numbers of genes per CNV or average CNV size (Figure S4).

**Figure 4:**
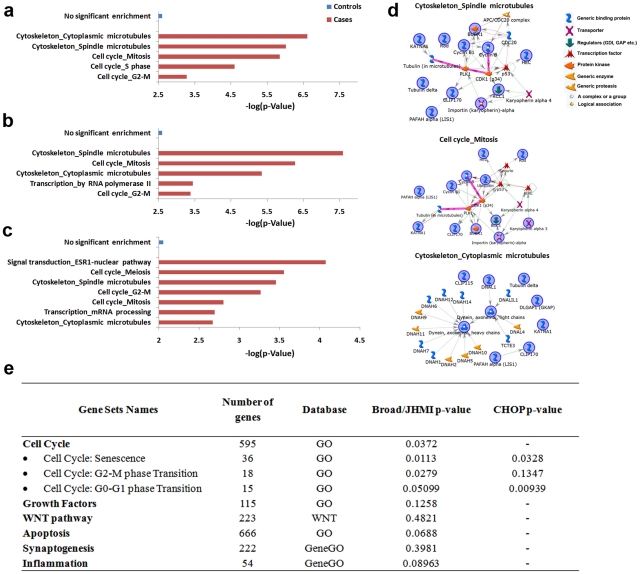
MetaCore process networks and network maps in autism based on gene deletions located in CNVs from DLPFC, and genetic association results. Autistic cases had significant enrichment of MetaCore process networks in genes contained within total CNVs (A) and filtered CNVs (i.e., not present in the Database of Genomic Variants) using PennCNV (B) and CNVision (C). Blue bars = controls; red bars = autistic cases. (D) MetaCore network analysis of the top three enriched MetaCore process networks in (B). Symbols in (D) represent gene types or associations. Genes with blue circles were identified autistic CNVs; genes without circles were summoned by the database to complete network. Pink lines = canonical pathway connections. (E) Gene sets tested in set-based association analysis using PLINK in Broad/JHMI and CHOP datasets, number of genes in each set, database from which genes were taken and p-values of associations.

Restricting analysis to male cases, genes identified in the autistic group were enriched in cell cycle (mitosis and S phase, including BUBR1, Cyclin B, BRCA1, RAD51, CRM1, RFC2 and LIS1) and cytoskeleton (spindle and cytoplasmic microtubules, including CLIP170, Tubulin alpha, Dynein light and heavy chains, DNAL1 and ROD) GeneGO Process Networks. No significant enrichment was identified in the gene content of controls (Figure 4). Similar network enrichment was noted in male autistic cases after filtering out common, non-pathogenic CNVs (Materials and Methods), while no significant network enrichment was found in controls (Figure 4). Notably, BRCA1 expression was found to be dysregulated in the young autistic brain (Figure 1) and predicted as deleted in the CNV analysis (Table S7). Subsequent enrichment analysis of CNVs present in male and female autistic and control cases yielded similar results (Figure S4). These results are consistent with the above described gene expression results showing abnormal expression of cell cycle and developmental pathways in the autistic cases.

To extend these genotypic findings and specifically test whether common variants of cell cycle genes are associated with autism, we determined whether common variants in processes involved in cell cycle and other hypothesized regulatory processes were associated with autism using two previously analyzed SNP datasets: one from the Autism Genetic Resource Exchange (AGRE) and National Institutes of Mental Health genotyped at the Broad Institute and Johns Hopkins Medical Institute (Broad/JHMI) and another from AGRE genotyped at the Children's Hospital of Philadelphia (CHOP) [55], [56]. Set-based association analysis was applied to the Broad/JHMI dataset (Materials and Methods). Four sets of genes postulated to be involved in brain overgrowth (cell cycle, WNT pathway, growth factors and apoptosis) were used as experimental sets for analysis, while other postulated pathological processes such as synaptogenesis and inflammation were used as controls.

We found nominal associations of only cell cycle-related genes with ASD (Figure 4, p = 0.037). Cell cycle genes were then subdivided into twenty-one subgroups. Genes regulating cell cycle senescence (p = 0.011), the G2-M phase transition (p = 0.028) and the G0-G1 phase transition (p = 0.051) in particular were associated with autism in the Broad/JHMI dataset (Figure 4). Because these analyses were not statistically corrected for the number of pathways considered, the findings were replicated for the three significant subcategories (p<0.05) of cell cycle genes using the independent CHOP dataset (Figure 4). Two of the subcategories (senescence and G0-G1 phase transition) were replicated (p<0.0328 and p<0.0093 respectively), while the third (G2-M phase transition) was not. These results suggest that abnormal expression of cell cycle and developmental pathways may originate from genetic variation.

## Discussion

Using genome-wide expression and CNV detection techniques, we discovered specific developmental pathways and processes in the prefrontal cortex that are significantly abnormal in younger autistic cases, other non-developmental processes that are dysregulated in older autism cases and, finally, processes common across younger and older ages in autism. Age-dependent gene expression alterations may underlie the differences in cellular and neuroanatomical pathologies between young and adult ages in autism that have been previously reported in MRI and postmortem research [13], [19]. In addition, we uncovered possible genotypic sources of developmental abnormalities in autism.

### The young autistic prefrontal cortex displays dysregulated developmental genes

It is widely held that maldevelopment of the prefrontal cortex, in conjunction with several other cortical and subcortical regions, plays a pivotal role in the social, communication, cognitive and emotion processing deficits in autism. In the present study, the young autistic prefrontal cortex displayed disturbances in critical developmental pathways and processes that govern cell number, cortical patterning and differentiation, including those regulating proliferation, cell cycle, DNA damage response and apoptosis and survival. We hypothesize that such dysregulation could be the basis of the 67% excess neuron numbers also reported for prefrontal cortex in children with autism [17]. The top dysregulated pathway map in the young autistic brain was the A2A receptor signaling pathway (Table 1, Diagnosis main effect map folders). Adenosine receptors play important roles for both brain development and function including the regulation of neuronal stem cell proliferation (via nitric oxide signaling) [57], synaptic plasticity, motor function, cognition and emotion-related behaviors. This pathway has been a therapeutic target for studies of other complex neurologic and psychiatric disorders [58].

We also found strong evidence of abnormalities in cortical patterning pathways that regulate normal anterior/posterior and dorsal/ventral patterning as well as right/left asymmetry. At young ages in autism, MRI evidence indicates disruption of normal cortical patterning with gray matter enlargement greatest in anterior dorsolateral prefrontal cortex and least in posterior occipital and ventral orbital prefrontal cortex [3], [6], [19]. It will be important to further investigate the possible role that genetic dysregulation of neural patterning may play in causing abnormal left/right functional and structural asymmetries in addition to abnormal anterior/posterior and dorsal/ventral gradients that have been reported in autistic infants, children and adults [3], [6], [14], [15], [19], [XPATH ERROR: unknown variable "next".]. Dysregulation of proliferation, cell cycle and apoptosis may be involved in early brain overgrowth in autism and excess neuron numbers in the prefrontal cortex [17] and could therefore contribute to dysfunction at the neural systems level [19], [26], [60], [61]

### The adult autism prefrontal cortex displays arrested growth and degeneration

In contrast to the younger autistic brain [62], the adult autistic brain shows evidence of neuron loss, reduced or arrested growth, cortical thinning and possible degeneration. It has been speculated that such pathologies may be related directly or indirectly to the earlier pathological overgrowth. It is known that the functions of genes are modulated by the age of the organism, such that dysregulation of the same gene in both young and adult autistic cases may have differing consequences. Genes such as BMP4 and BTRC, which have important functions in neurodevelopment, may regulate recovery functions such as neurogenesis in the adult brain [63]–[65]. Furthermore, we found that genes involved in cell differentiation, including RELN, BMP4, NODAL and NTRK3, and tissue remodeling and wound repair are dysregulated in adults. Though the literature on adult neurogenesis and cell differentiation is sparse, recent studies propose that the brain activates these mechanisms in response to injury in the neocortex [66], [67]. In the context of brain overgrowth, these data suggest the hypothesis that reactive reorganization of functional genetic networks and processes may occur in the adult autistic brain. Such functional reorganization of genetic modules or networks has recently been described for systems that regulate DNA-damage responses [68].

### Age-independent differential expression represents pathological developmental processes

To complement analyses of age-specific expression pathology in autism, we also identified gene expression abnormalities in autism that were age independent. Among the most abnormally expressed genes were those that regulate DNA-damage response, apoptosis and immune system response functions. These genetic systems play important roles during development of the central nervous system [69]–[71] and suggest that the brain in autism arises from defects during this critical period. Several lines of evidence indicate that immune system responses closely participate in the neuropathogenesis of ASD both at young and adult ages. Although little is known about this role, both inflammatory and autoimmune mechanisms are necessary for normal neurodevelopment and are found to be altered in ASD [72]. Microglia activation and increases in inflammatory cytokine and chemokine production have direct effects on neuronal development by affecting cellular proliferation, migration and differentiation as well as synapse formation [72]. For example, TNF-α can modulate neuronal cell proliferation or cell death and plays an important role in synaptic pruning [73], [74]. In addition, auto-antibodies may affect receptor function, activate neuronal and glial cells and induce cellular damage or death [72].

More work remains to be done, however, to uncover the significance of these pathways in the postnatal brain. These results may point to mechanisms in the brain that are continuously pathological throughout the lifespan in autism and suggest possible candidates for genetic therapies.

### Genes in common with a previous genetic study support pathway results

While large sample *in vivo* studies, such as Pinto et al. [26], have identified potential functional genomic abnormalities in autism, biological validation requires postmortem studies, such as the present one and Voineagu et al. [20]. The comparison of findings between the present study and Voineagu et al. [20] demonstrates a consistent overlap involving several pathways including repair- and immune-related processes.

We speculate that the aberrances in these genes, which may be distinct from individual to individual, may collectively underlie the repair or response mechanisms in the mature autistic brain. Commonalities across autistic brains may converge at the level of disturbances in these genetic pathways, and monitoring most commonly affected pathways across functional genomic studies may help subgroup different pathogenic mechanisms.

Though these genetic pathways with high impact on autism susceptibility may be similar between studies, there are also copious pathways that are disparate. The differences in the results between our study and Voineagu et al. [20] may be due to genetic heterogeneity, different criteria for sample selection and age and gender distribution. For example, although our studies assayed a similar number of postmortem cases, our sample consisted of all male autistic and control cases, while that of Voineagu et al. [20] consisted of 36% female autistic and 6% female control cases. Also, to identify gene expression abnormalities in the young autistic brain, we compared expression in the young autistic brain to the young control brain, while Voineagu et al. [20] lacked young controls. Thus, some effects that we observed may not have been identified in Voineagu et al. [20] because of differences in study design.

### Copy number anomalies in autism

Our analyses using mRNA and DNA from the same cortices suggest that a large and heterogeneous array of genes and gene expression abnormalities regulate multiple foundational prenatal processes critical to cortex formation. Although DNA defects vary from autistic case to case, the diverse genetic deletions seem to underlie a relatively common biological theme, hitting a shared set of gene pathways that impact cell cycle, DNA damage detection and repair, migration, neural patterning and cell differentiation. The set of functional gene pathways identified by our direct analyses of autistic brain tissue are consistent with those identified by CNV pathway enrichment analyses in living autistic patients [26].

### Conclusion

In this study, we found evidence for distinct gene expression anomalies in the prefrontal cortex of young autistic individuals that may underlie structural and functional maldevelopment of this and other association cortices. Our evidence shows that some important gene expression abnormalities in the prefrontal cortex change with age in autism and such changes may be related to findings in other studies that report a shift in morphology and function in the adult autistic brain. Enrichment of CNVs and association analysis of cell cycle gene sets, in accordance with early expression defects of cell cycle mechanisms, suggest that genetic variation may contribute to this dysregulation. We also found that irrespective of age, the expression of atypical developmental processes distinguish the autistic brain from that of control individuals. The modulation of these expression mechanisms may underlie the abnormal brain growth trajectory in autism. Further knowledge of the specific developmental neurobiological mechanisms behind the age-dependent anomalies reported here could point to distinct early developmental processes that lead to autism, uncover mechanisms that respond to early pathologies in the mature brain and suggest novel molecular targets for prevention strategies and treatment over the course of the disorder.

## Materials and Methods

### Gene expression analysis

#### Ethics statement

All cases are deceased and are deidentified by the brain banks where tissue was obtained. However, the same human protections procedures were employed as for live subjects. Research procedures employed in this study were approved by the institutional review board of the University of California, San Diego (protocol number 091205).

#### RNA extraction and quantification

A full description of the methods is provided in Text S1. Extraction of total RNA from 5–10 mg of frozen brain tissue from grey and white matter of prefrontal cortex of 57 postmortem autism and control cases aged 2–56 years was performed using the Ambion MELT kit according to manufacturer's instructions. Select samples were analyzed using Bioanalyzer (Agilent) according to the manufacturer's protocol for quality control and quantification (Table S1). RNA from remaining samples was quantified using a NanoDrop spectrophotometer.

#### Microarray processing

Four hundred fifty ng of total RNA from each of 57 cases was submitted to Illumina, Inc. (San Diego, CA) for DASL-based labeling and hybridization to the Illumina HumanRef8 v3 microarray. Using biotinylated random primers and oligo-dT, 200 ng RNA was converted to cDNA. The biotinylated cDNA was then immobilized to a streptavidin-coated solid support and annealed by 24526 pairs of oligonucleotides (18626 genes). Following extension and ligation, the ligated oligonucleotides were PCR-amplified with biotinylated or fluorophore-labeled universal primers and captured using streptavidin paramagnetic beads. Finally, the products were washed and denatured before hybridization to the BeadChips at 58°C for 16 hours. A BeadArray Reader extracted images and read fluorescence intensities, and all data was uploaded into BeadStudio software for quality control and processing.

#### Data preprocessing

Raw data exported from Illumina's BeadStudio underwent outlier removal, log2 transformation and quantile normalization as implemented in the lumi package for R/Bioconductor [75]. To simultaneously remove variance attributed to batch and seizures, we performed batch and covariate correction using ComBat [76]. Multivariate Distance Matrix Regression [77] with 10,000 permutations confirmed that variance attributable to batch and seizures were decreased following correction. Detailed information regarding the methodology for normalizing gene expression data and removing outliers is provided in Chow et al. [78].

### Statistical analysis

Filtering and differential expression analyses were performed using BRBArrayTools (http://linus.nci.nih.gov/BRBArrayTools.html). Filtering was performed based on variance and minimum intensity. Probes with <20% of expression values having at least a 1.5-fold up- or down-regulation from the gene's median value across cases, and probes with a minimum log intensity of less than 15 were excluded.

We identified 1086 probes with an interaction effect of age and diagnosis by performing a two-way analysis of variance with 2 levels of diagnosis (autism and control) and 2 levels of categorical age (2–14 years and 15–56 years, n = 33). Interaction probes of p<0.05, corresponding to an empirical FDR of 0.27 [79], were selected for posthoc analysis to examine which differences were driven by the young autism and young control groups and the adult autism and adult control groups by t-tests (p<0.05) to investigate gene expression changes in the young and adult autistic brain. Probes exhibiting a main effect of diagnosis across age groups were also identified (p<0.05, FDR = 0.13).

### Enrichment analysis

Enrichment analyses were performed using the MetaCore software suite (www.genego.com/metacore.php) and DAVID (http://david.abcc.ncifcrf.gov/) to examine the biological and functional relevance of differentially expressed genes. These genes were uploaded into MetaCore and filtered for known expression specific to the brain or the fetal brain. The default background gene list was used for all enrichment analyses. All results met threshold of corrected p<0.05 and FDR<0.1 [80]. Gene references used to confirm categorizations by MetaCore are listed in Table SXPATH ERROR: unknown variable "checknextn".XPATH ERROR: unknown variable "checknextn".

### Independent validation of microarray results

RNA from 1 male autistic and 1 control case both 31 years old were analyzed using SYBR green RT-PCR to validate the intensity values detected by microarray of 19 genes (Figure S2). Primer3 software [81] was used to design primers across splice junctions to produce amplicons of ∼200 bp.

One mg of total RNA was used for cDNA synthesis using random hexamers and AMV reverse transcriptase. An equivalent of 50 ng of RNA was processed by qPCR using Roche's LightCycler rapid thermal cycler system (Roche Diagnostics Ltd, Lewes, UK) according to the manufacturer's instructions in a 96-well, 10 µL format using standard PCR conditions. One µL of cDNA template, 250 nM of forward and reverse primer and 5 µL of PCR Master Mix (Roche) were mixed for each reaction.

We took the geometric mean of all reference genes and the difference between this mean and the average intensity of experimental genes to find the delta Ct for each experimental gene. Subsequently, log2 fold change was assessed using -(T-C) where T = delta Ct of gene of the autistic case and C = delta Ct of gene of the control case. Using Spearman's rank correlation, the log2 fold changes of these 19 genes across qPCR and microarray platforms were found to be correlated at R = 0.78 (p = 0.000075, DF = 17; Figure S2).

### Comparison between studies

Unique ProbeIDs of differentially expressed in the ‘initial’ dataset from Voineagu et al. [20] (file = ‘nature10110-s3.xls’) were compared with the differential expression gene list in the present study comparing all autism and control cases. Genes overlapping in each study with the present one were subjected to enrichment analysis in MetaCore.

### Copy Number Variation (CNV) detection and association analysis

Fifty-five samples were genotyped on the Illumina 660 Bead Array (Illumina Inc., San Diego, California) and SNP calls were made using the Illumina Genome Studio software.

CNV calls were first obtained using the PennCNV software (http://www.openbioinformatics.org/penncnv/) implementing the wave adjustment procedure via the “–gcmodel” argument [53] and then using the CNVision pipeline [54] to select for high confidence CNV calls.

We first analyzed only male cases that passed QC (12 autism and 12 controls; Figure 4) and identified a total of about 850 CNVs. Known nonpathogenic regions reported in the Database of Genomic Variants (http://projects.tcag.ca/cgi-bin/variation/gbrowse/hg18) were filtered without upper size limits. No large-size CNVs (>1 Mb) were identified in single cases that could potentially drive the gene enrichment results. We next analyzed males and female cases together (14 autism and 16 controls; Figure S4) using the same parameters and identified a total of about 1300 CNVs and then proceeded with the filtering of common regions. We also excluded the possibility of a biased CNV detection given the small sample size (Text S1 and Figure S4).

Due to possible high false positive rates, we re-analyzed all cases using CNVision, a recently described analysis pipeline [54] (www.cnvision.org) that merges results of PennCNV, QuantiSNP and GNOSIS. The analysis of the male cases yielded about 350 CNVs (11 autism and 13 controls). Gene enrichment was performed considering both the gene content of gene-rich CNVs and the nearest gene at the 5- and 3-prime end of gene-desert CNVs. As performed with PennCNV alone, we next analyzed male and female cases together but were unable to compare the gene enrichment of the two categories due to the significant difference in the number of cases that passed QC (12 autism versus 19 controls).

GO enrichment of the gene content of these regions was also performed using the MetaCore software suite (FDR<0.01 and p<0.05). GeneGO Network Processes of each of these gene lists are reported in Figure 4 and Figure S4.

### Gene association analysis

For gene association analysis we utilized the AGRE-NIMH Broad/Johns Hopkins Medical Institute (Broad/JHMI) sample as our experimental dataset using original quality control filters and the Children's Hospital of Philadelphia (CHOP) sample dataset as a replication sample, as previously described [56].

Gene sets were created using Gene Ontology (GO; http://www.geneontology.org/), GeneGO (http://www.genego.com/metacore.php) and the Wnt homepage (http://www.stanford.edu/group/nusselab/cgi-bin/wnt/). We used PLINK [82] (http://pngu.mgh.harvard.edu/~purcell/plink/) to retrieve the SNPs within a window of 20 kb around each gene and to perform set-based association analysis.

Significantly associated pathways (p<0.05) in the cell cycle gene set were then broken down into 21 subsets of genes denoting specific phases of the cell cycle using Ingenuity and set-based tests were again performed on the experimental dataset. Subsets that were found to be significant in the Broad/JHMI sample were then tested on the CHOP sample for replication using the same set-based analysis.

## Supporting Information

Figure S1 Venn diagram showing differentially expressed genes in young autism vs. young control, adult autism vs. adult control and diagnosis main effect ANOVA analyses.
(TIF)

Figure S2 Log2 fold change correlations of selected genes detected by microarray and RT-PCR, and primer sequences. (A) Log2 Fold change detected by RT-PCR is depicted on the x-axis, and change detected by microarray is on the y-axis. Spearman's rank correlation detected R = 0.78 (p = 0.000075, DF = 17) correlation between microarray and qPCR detection of fold change. (B) Forward and reverse primer sequences for 19 experimental and 3 reference genes used for RT-PCR to validate gene expression microarray results.
(TIF)

Figure S3 Third most significant map folder of diagnosis main effect analysis. Graph on left shows fold change of genes in Immune Response Map Folder. This map folder was third most significant in the comparison between all autistic and all control cases. From each category of differentially expressed genes in the all autistic vs. all control comparison (Table S5), networks were created using MetaCore Network Analysis. Genes with blue circles were differentially expressed; genes without circles were summoned by the database to complete network.
(TIF)

Figure S4 Enrichment of CNVs detected in the DLPFC of male and female samples. Metacore analysis of GeneGO Processes significantly represented in genes contained within: (A) total CNVs and B) filtered (i.e., not present in the Database of Genomic Variants) CNVs. Blue bars, controls. Red bars, autistic cases. C) Average number of CNVs in autistic cases and controls. D) Average number of genes per CNV in autistic and control cases. E) Average CNV size comparison between autistic cases and controls. C–E) No statistically significant differences were found between autistic cases and controls, suggesting non-biased CNV detection potentially due to the small sample size. F) Size distribution of the filtered CNVs in the autistic cases is shown.
(TIF)

Table S1 Demographics and clinical information of postmortem cases. (A) ID, diagnosis, age, gender, study, DLPFC RNA integrity number (RIN), basis of diagnosis, ADI measures, and intellectual disability for cases in present study are documented. RIN #1 was performed at the UCSD Biogem core, RIN #2 was performed at UCSF in the Wynshaw-Boris lab, and RIN #3 was performed at the Allen Institute. ADI-R = Autism Diagnostic Interview-Revised; ADOS = Autism Diagnostic Observation Schedule; CNV = copy number variation; ISH = in situ hybridization; Comm = Communication; R & R = Restricted and Repetitive behavior; DLPFC = Dorsolateral Prefrontal Cortex; RIN = RNA integrity number; M = male; F = female; v = verbal; nv = nonverbal; Y = yes; N = no; TARF = existing diagnostic status on donation from The Autism Research Foundation, no other records available; ATP = existing diagnostic status on case from the Autism Tissue Program, no documents available at this date. Pink X = case passed quality control measures for gene expression/CNV analysis; black X = case was processed but did not pass quality control measures for CNV analysis. (B). Detailed clinical information of autistic cases. Available clinical information, including information from the ADI-R assessment, noted seizure activity and intellectual disability, is provided for autistic cases in this study. Assessments were made by a clinical psychologist (C.C.B.). ADI = Autism Diagnostic Interview; RIN = RNA Integrity Score; HX = History; DO = Diagnosis; ADI Comm Score = ADI-R Communication Score; ADI R&R Score = ADI-R Restricted and Repetitive Behavior Score; IVF = In Vitro Fertilization. (C) Detailed clinical information of autistic and control cases. Available clinical information is provided for autistic and control cases in this study. Assessments were made by a clinical psychologist (C.C.B.). HX = History; DO = Diagnosis; IVF = In Vitro Fertilization; PMI = Postmortem Interval.
(PDF)

Table S2 Differentially expressed genes in young autistic cases. All 102 differentially expressed genes in the posthoc comparison passing a threshold of p<0.05 between young autism and young control cases of genes showing an effect of interaction between diagnosis and categorical age. Gene symbols, Illumina probe IDs, p-Value of difference, cytogenetic bands and fold changes are listed. Bold genes are shown in Figure 1.
(PDF)

Table S3 Dysregulated genes of young autistic cases and previously identified candidate genes and loci. Loci of 102 differentially expressed genes in the posthoc comparison and references of previous linkage/association/CNV studies implicating the candidate locus and references of other genes neighboring the candidate loci are listed. NEGATIVE = negative association/linkage.
(PDF)

Table S4 Top downregulated and upregulated genes in adult autistic cases. Genes showing greatest fold change difference in the posthoc comparison passing a threshold of p<0.05 between adult autism and adult control cases of genes showing an effect of interaction between diagnosis and categorical age. Gene symbols, Illumina probe IDs, p-Value of difference, cytogenetic bands and fold changes are listed.
(PDF)

Table S5 Top downregulated and upregulated genes of male autistic cases across all young and older ages (2 to 56 years). Genes showing greatest fold change differences and a main effect of diagnosis passing a threshold of p<0.05 between autism and control cases, Illumina probe IDs, p-value of difference, cytogenetic bands and fold changes are listed.
(PDF)

Table S6 Significant dysregulated pathways of autistic cases across all young and older ages (2 to 56 years). Dysregulated pathways enriched in genes showing a main effect of diagnosis (p<0.05; FDR<0.1) identified using the MetaCore software suite between autism and control cases, p-value and network objects are listed. Pathways are sorted by pathway category name.
(PDF)

Table S7 Chromosomal locations of CNV regions found in all autistic cases (males and females). Chromosomal location, number (N) of SNPs, size of the SNP in base pairs (bp) and annotated gene content of CNV regions found in all autistic cases (males and females) are listed. Overlapping regions with the same gene content but different breakpoints are listed as distinct CNVs. Enrichment results are presented in Figure 4.
(PDF)

Table S8 Chromosomal locations of CNV regions found in all control cases (males and females). Chromosomal location, number (N) of SNPs, size of the SNP in base pairs (bp) and annotated gene content of CNV regions found in all control cases (males and females) are listed. Overlapping regions with the same gene content but different breakpoints are listed as distinct CNVs. Enrichment results are presented in Figure 4.
(PDF)

Table S9 References for genes in noted pathways and networks. Important genes from the pathways and networks identified by gene expression analyses are listed. Gene category, references, and analysis corresponding to these genes are presented. Gex = gene expression.
(PDF)

Text S1 Supporting materials. Supplementary methods, acknowledgements and figures are provided.
(DOCX)
