# Supplementary figures and images for: Age-Dependent Brain Gene Expression and Copy Number Anomalies in Autism Suggest Distinct Pathological Processes at Young Versus Mature Ages
Source: PLoS Genet. 2012 Mar 22;8(3):e1002592. doi: 10.1371/journal.pgen.1002592 (PMC3310790; doi:10.1371/journal.pgen.1002592)

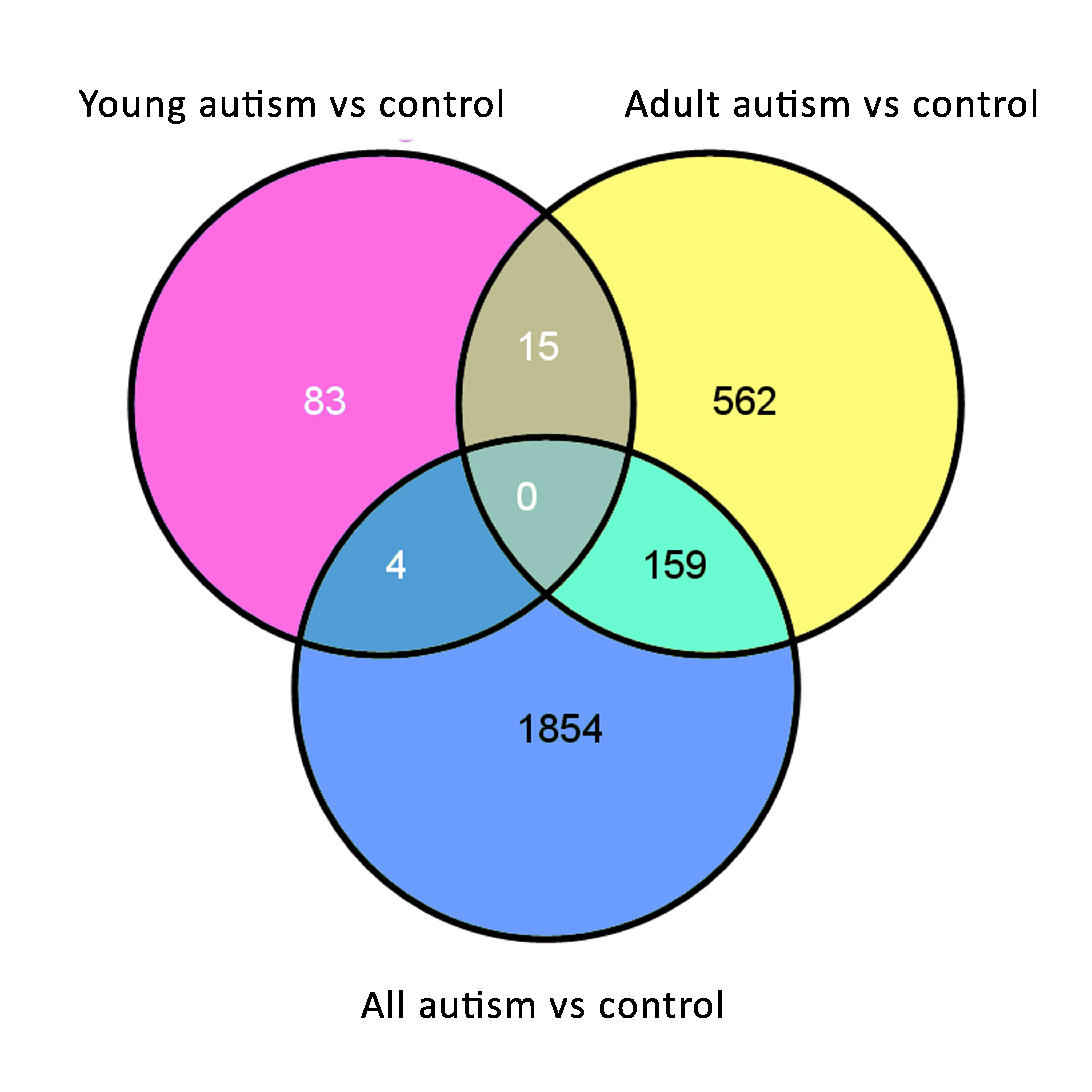

Supplement: Figure S1 — Venn diagram showing differentially expressed genes in young autism vs. young control, adult autism vs. adult control and diagnosis main effect ANOVA analyses. (TIF) [file pgen.1002592.s001.tif]

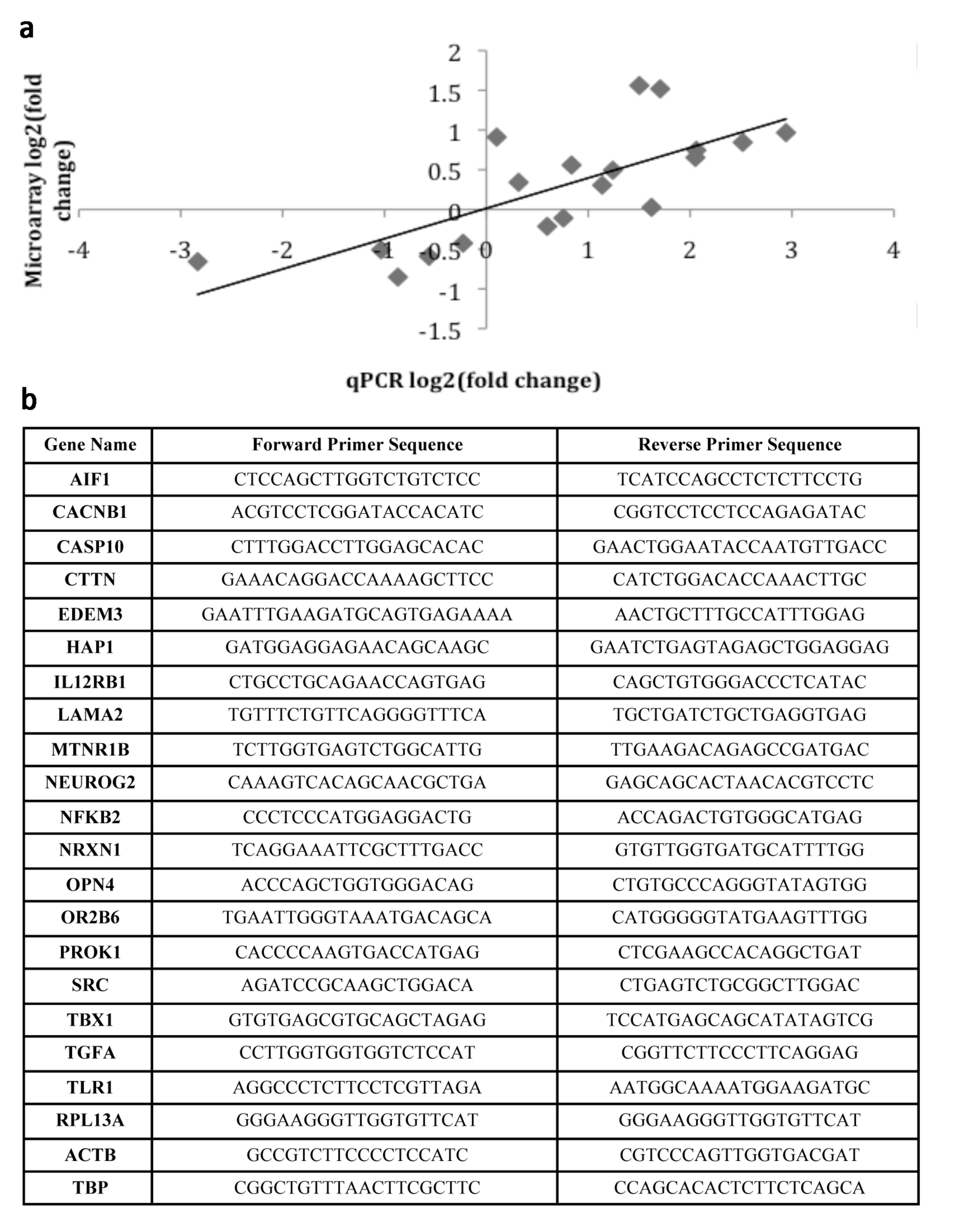

Supplement: Figure S2 — Log2 fold change correlations of selected genes detected by microarray and RT-PCR, and primer sequences. (A) Log2 Fold change detected by RT-PCR is depicted on the x-axis, and change detected by microarray is on the y-axis. Spearman's rank correlation detected R = 0.78 (p = 0.000075, DF = 17) correlation between microarray and qPCR detection of fold change. (B) Forward and reverse primer sequences for 19 experimental and 3 reference genes used for RT-PCR to validate gene expression microarray results. (TIF) [file pgen.1002592.s002.tif]

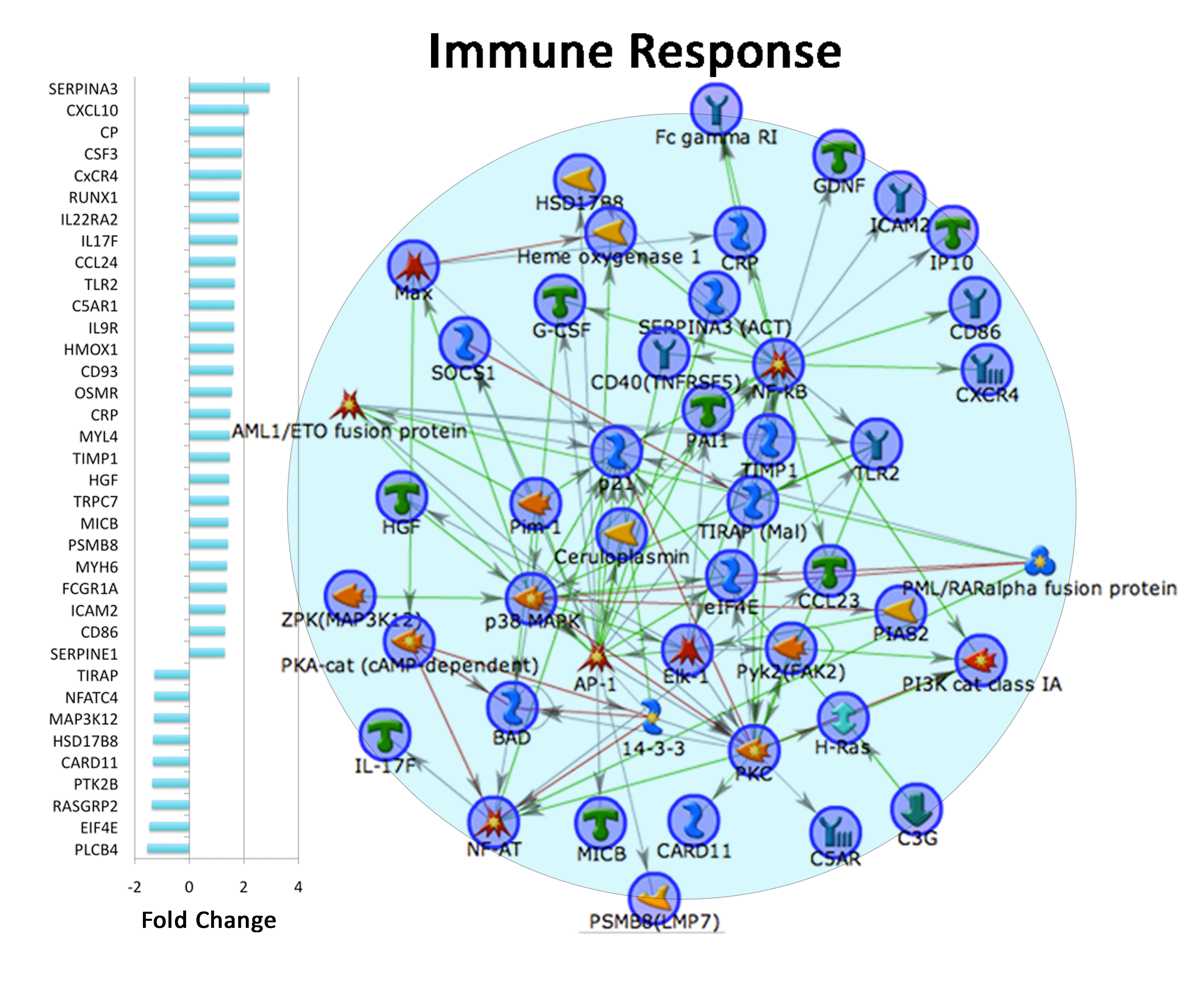

Supplement: Figure S3 — Third most significant map folder of diagnosis main effect analysis. Graph on left shows fold change of genes in Immune Response Map Folder. This map folder was third most significant in the comparison between all autistic and all control cases. From each category of differentially expressed genes in the all autistic vs. all control comparison (Table S5), networks were created using MetaCore Network Analysis. Genes with blue circles were differentially expressed; genes without circles were summoned by the database to complete network. (TIF) [file pgen.1002592.s003.tif]

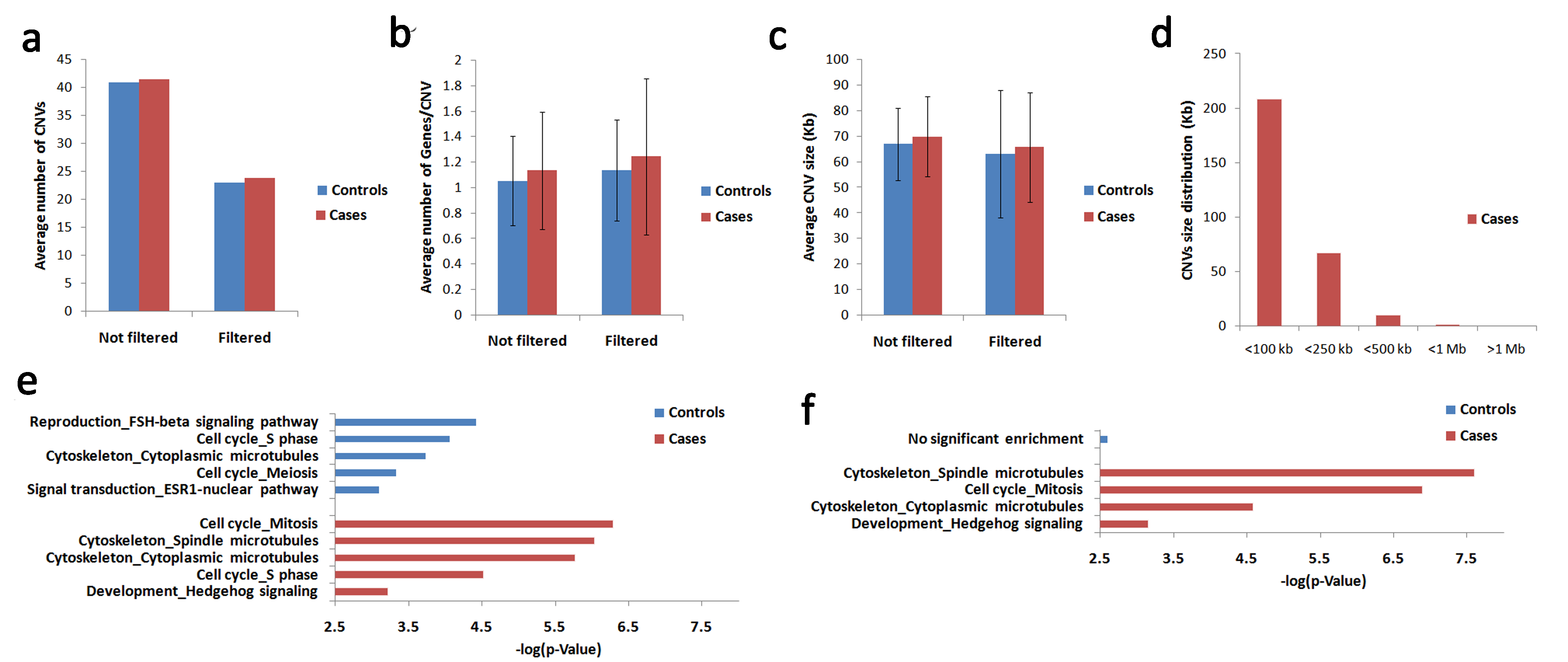

Supplement: Figure S4 — Enrichment of CNVs detected in the DLPFC of male and female samples. Metacore analysis of GeneGO Processes significantly represented in genes contained within: (A) total CNVs and B) filtered (i.e., not present in the Database of Genomic Variants) CNVs. Blue bars, controls. Red bars, autistic cases. C) Average number of CNVs in autistic cases and controls. D) Average number of genes per CNV in autistic and control cases. E) Average CNV size comparison between autistic cases and controls. C–E) No statistically significant differences were found between autistic cases and controls, suggesting non-biased CNV detection potentially due to the small sample size. F) Size distribution of the filtered CNVs in the autistic cases is shown. (TIF) [file pgen.1002592.s004.tif]
