# Supplementary material for: Age-Dependent Brain Gene Expression and Copy Number Anomalies in Autism Suggest Distinct Pathological Processes at Young Versus Mature Ages
Source: PLoS Genet. 2012 Mar 22;8(3):e1002592. doi: 10.1371/journal.pgen.1002592 (PMC3310790; doi:10.1371/journal.pgen.1002592)
Supplement: Table S1 — Demographics and clinical information of postmortem cases. (A) ID, diagnosis, age, gender, study, DLPFC RNA integrity number (RIN), basis of diagnosis, ADI measures, and intellectual disability for cases in present study are documented. RIN #1 was performed at the UCSD Biogem core, RIN #2 was performed at UCSF in the Wynshaw-Boris lab, and RIN #3 was performed at the Allen Institute. ADI-R = Autism Diagnostic Interview-Revised; ADOS = Autism Diagnostic Observation Schedule; CNV = copy number variation; ISH = in situ hybridization; Comm = Communication; R & R = Restricted and Repetitive behavior; DLPFC = Dorsolateral Prefrontal Cortex; RIN = RNA integrity number; M = male; F = female; v = verbal; nv = nonverbal; Y = yes; N = no; TARF = existing diagnostic status on donation from The Autism Research Foundation, no other records available; ATP = existing diagnostic status on case from the Autism Tissue Program, no documents available at this date. Pink X = case passed quality control measures for gene expression/CNV analysis; black X = case was processed but did not pass quality control measures for CNV analysis. (B). Detailed clinical information of autistic cases. Available clinical information, including information from the ADI-R assessment, noted seizure activity and intellectual disability, is provided for autistic cases in this study. Assessments were made by a clinical psychologist (C.C.B.). ADI = Autism Diagnostic Interview; RIN = RNA Integrity Score; HX = History; DO = Diagnosis; ADI Comm Score = ADI-R Communication Score; ADI R&R Score = ADI-R Restricted and Repetitive Behavior Score; IVF = In Vitro Fertilization. (C) Detailed clinical information of autistic and control cases. Available clinical information is provided for autistic and control cases in this study. Assessments were made by a clinical psychologist (C.C.B.). HX = History; DO = Diagnosis; IVF = In Vitro Fertilization; PMI = Postmortem Interval. (PDF) [file pgen.1002592.s005.pdf]

Table S1A

Case ID	Alternate ID	Diagnosis	Age (Years)	Gender	Gene Expression	CNV	DLPFC RIN #1	DLPFC RIN #2	DLPFC RIN #3	Basis of Diagnosis	ADI Social	ADI Comm Score	ADI R & R Score	Intellectual Disability
**B6399**	AN03345	Autism	2	M	X	X	6	3.8	7.6	ADI-R	14	9 (NV)	6	N
**UMB1349**		Autism	5	M	X	X	-	-	8.7	ADOS	No ADI	No ADI	No ADI	Y
**B1469**	AN13364	Autism	5	M	X	X	2.3	2.8	-	TARF	No ADI	No ADI	No ADI	No records
**UMB4849**		Autism	7	M	X	X	6.3	6.6	-	ADI-R	22	18 (V)	8	Y
**UMB1174**		Autism	7	F	X	X	-	-	-	ADI-R	22	11 (NV)	6	Y
**B5666**	AN19511	Autism	8	M	X	X	5	6.7	7.8	ADI-R	19	14 (V)	4	N
**UMB4231**		Autism	8	M	X	X	-	6.5	7.4	Medical Records	No ADI	No ADI	No ADI	Y
**UMB4721**		Autism	8	M	X	X	2.4	1	-	ADI-R	24	10 (NV)	10	Y
**UMB797**		Autism	9	M	X	X	-	-	6.9	ADI-R	24	20 (V)	6	N
**B4925**	AN16641	Autism	9	M	X	X	2.2	4.5	-	ADI-R	24	13 (NV)	4	Y
**UMB1182**		Autism	9	F	X	X	-	-	-	Medical Records	No ADI	No ADI	No ADI	Y
**UMB4899**		Autism	14	M	X	X	-	-	8.5	ADI-R	22	14 (NV)	8	Y
**B7079**	AN04682	Autism	15	M	X	X	5.7	-	5.7	ATP	No records available	No records available	No records available	No records available
**B6337**	AN09730	Autism	22	M	X	X	-	-	-	ADI-R	22	14(NV)	6	Y
**B6994**	AN08166	Autism	28	M	X	X	3	-	-	ADI-R	22	16 (V)	5	N
**B5173**	AN08792	Autism	30	M	X	X	-	-	-	ADI-R	10	12 (NV)	3	Y
**B6677**	AN11989	Autism	30	M	X	X	-	3.5	-	ADI-R	26	22 (V)	12	Y
**UMB1445**		Autism	45	M	X	X	-	2.4	-	Medical Records	No ADI	No ADI	No ADI	Y
**B7109**	AN17254	Autism	51	M	X	X	4	5.4	-	ADI-R	27	19 (V)	6	N
**B4498**	AN01093	Autism	56	M	X	X	-	-	-	ADI-R &	25	12 (NV)	7	Y
**UMB1377**		Control	3	F	X	X	-	-	-	-	-	-	-	-
**UMB1185**		Control	4	M	X	X	2.1	2.3	6.8	-	-	-	-	-
**UMB4670**		Control	4	M	X	X	2.2	6.3	-	-	-	-	-	-
**UMB4898**		Control	7	M	X	X	5.1	6.8	7.7	-	-	-	-	-
**UMB1860**		Control	8	M	X	X	-	4.4	7.5	-	-	-	-	-
**UMB1674**		Control	8	M	X	X	-	3.2	-	-	-	-	-	-
**UMB1650**		Control	10	M	X	X	2.1	2.2	7.3	-	-	-	-	-
**UMB1714**		Control	12	M	X	X	2.5	-	7.7	-	-	-	-	-
**UMB4787**		Control	12	M	X	X	6.4	-	6.5	-	-	-	-	-
**UMB4722**		Control	14	M	X	X	1.8	1.9	7.7	-	-	-	-	-
**UMB4638**		Control	15	F	X	X	-	1.1	8.8	-	-	-	-	-
**UMB1796**		Control	16	M	X	X	-	7.1	-	-	-	-	-	-

**B6756**	AN07591	Control	16	M	X	X	-	-	-	-	-	-	-	-
**B6207**	AN17425	Control	16	M	X	X	-	-	-	-	-	-	-	-
**B5251**	AN03217	Control	19	M	X	X	3.5	4.3	-	-	-	-	-	-
**UMB1649**		Control	20	M	X	X	4.9	5.6	-	-	-	-	-	-
**BTB3960**		Control	25	F	X	X	-	-	-	-	-	-	-	-
**UMB818**		Control	27	M	X	X	1.9	-	-	-	-	-	-	-
**B5873**	AN19760	Control	28	M	X	X	-	-	-	-	-	-	-	-
**B5334**	AN15622	Control	30	M	X	X	5.7	5.7	-	-	-	-	-	-
**B5352**	AN12137	Control	31	M	X	X	3.8	6	-	-	-	-	-	-
**B5813**	AN01410	Control	41	M	X	X	5.2	5.1	-	-	-	-	-	-
**B6208**	AN19440	Control	50	F	X	X	-	-	-	-	-	-	-	-
**B4756**	AN10606	Control	56	M	X	X	5.9	5.6	-	-	-	-	-	-
**B6860**	AN13295	Control	56	M	X	X	6.3	6.8	-	-	-	-	-	-

Table S1B

Case ID	Alternate ID	Age	Sex	Diagnosis	Basis of Diagnosis	Verbal Compre-hension	Verbal Expression	Age of 1st Concern	Language Regression	IQ	Self injury	Engagement in Appropriate Activity	Simplex/ Multiplex	Seizures
**B6399**	AN03345	2	M	Autism	ADI-R	Some words and commands	Fewer than 5 words total/ speech not used daily	15m	N	at 27 months got IQ of 78 on Merrill-Palmer	N	Limited constructive play with repetitive activities	Multiplex	N
**UMB1349**		5	M	Autism	ADOS	Some words and commands	Fewer than 5 wds total / speech not used daily	<24m	Y	16m age equiv. @ 33m	Y	Limited constructive play with repetitive activity and motor stereotypies	Simplex	N
**B1469**	AN13364	5	M	Autism	TARF	No information-TARF	No information-TARF	No information-TARF	No information-TARF	No information-TARF	No information-TARF	No information-TARF	No information-TARF	No information-TARF
**UMB1174**		7	F	Autism	ADI-R	Many words and some commands	Fewer than 5 wds total / speech not used daily	5m	Y	13-17m age equiv. @ approx. 31m	N	Engages in passive, but otherwise appropriate activity such as tv or radio	Simplex	N
**UMB4849**		7	M	Autism	ADI-R	Many words (more than 50) and some commands	Functional use of language with three or more word phrases restricted in frequency and contexts used; echolalia	2 yrs	reported loss of 4 words acquired at age 2 (insufficient language to code true regression on ADI)	<50 overall on Bayley Scales	N	Limited constructive play with repetitive activities	Multiplex	N
**B5666**	AN19511	8	M	Autism	ADI-R	Most words and language	Functional use of language involving three or more word phrases; occasional echolalia	36m	N	No scores available	N	Initiates a limited range of appropriate activities	Simplex	teacher thought he was having petite mals -- EEG showed "unusual brain activity but not seizures

														"
**UMB4231**		8	M	Autism	Medical Records	No ADI- neurologist report states child is able to follow a few commands	No ADI – neurologist report states child is non-verbal	No ADI	None Reported	No scores available	None reported	No ADI- neurologist reports states overactivity and spinning of objects	Simplex	Neuro report states dysmorphic features, large head, history of mental retardation
**UMB4721**		8	M	Autism	ADI-R	Many words (more than 50) and some commands	Fewer than 5 wds total / speech not used daily	18m	N	No scores available	N	Engages in passive, but otherwise appropriate activity	Simplex	N
**UMB1182**		9	F	Autism	Medical Records	Not reported	Approximately 50-75 reported at age 7, with use of the Picture Exchange Communication System (PECS)	Not reported	None reported	No scores available	N	Restricted and stereotypic play	Simplex	N
**UMB797**		9	M	Autism	ADI-R		Functional use of language involving three or more word phrases	30m	N	No scores available	N	Initiates a limited range of appropriate activities, some repetitive	Simplex	N
**B4925**	AN16641	9	M	Autism	ADI-R	Many words (more than 50) and some commands	Fewer than5 wds total / speech not used daily	12m	N	Catell = 36; Mullen <39		Engages in passive activities (watching videos); active play, and repetitive activities	Simplex	Y
**UMB4899**		14	M	Autism	ADI-R		Fewer than 5 words total / speech not used daily	12m	Y		Y	Engages in repetitive play.	Multiplex	Y
**B7079**	AN04682	15	M	Autism	ATP	No records yet available	No records yet available	No records yet available	No records yet available	No records yet available	No records yet available	No records yet available	No records yet available	ATP has not yet completed collection of clinical documents

**B6337**	AN09730	22	M	Autism	ADI-R	Some words and commands	Use of familiar words and names (~20)	14m	N	Not testable; adaptive skills @ 1st percentile	N	Engages in repetitive activities	Simplex	Y
**B6994**	AN08166	28	M	Autism	ADI-R	Most words and language	Functional use of language involving three or more word phrases			IQ tests: 57 at age 10 but in later years scored 80 Full Scale			Simplex	Y
**B5173**	AN08792	30	M	Autism	ADI-R		Fewer than5 words total / speech not used daily	5m	N	Scores not available	Y	Engages in repetitive activities	Simplex	Y
**B6677**	AN11989	30	M	Autism	ADI-R		Some single words	24m	N	Mental age 16 months at 34 months chronological age			Simplex	Y - "related to brain tumor"
**UMB1445**		45	M	Autism	Medical Records	Many words (more than 50) and some commands	Fewer than5 wds total / speech not used daily	Not reported	N	Severe MR on standardized assessments			Multiplex probably; see notes	N
**B7109**	AN17254	51	M	Autism	ADI-R		Functional use of language used on a daily basis involving three or more word phrases	9m	N	No IQ measure; described as HFA, drove and had a job as early as age 16	N		Simplex	N
**B4498**	AN01093	56	M	Autism	ADI-R & Records	Many words (more than 50) and some commands	Fewer than5 words total / speech not used daily	18m	Y	Untestable	N	Engages in passive activities ; some repetitive interests (ceiling fans)	Simplex (only child)	Y

Table S1C

Case ID	Alternate ID	Age	Gender	Diagnosis	Race	Hx Meds	Prenatal Hx	IVF/ Fertility Hx	Psych DO in Family	Cause of Death	PMI	Other
**B6399**	AN03345	2	M	Autism	Japanese, Native American, Caucasian, Hispanic	None reported	Repeated C-Sec, mother miscarried twice; slight jaundice at birth, slightly blue at birth, got oxygen	N	Maternal 3rd cousin with autism.	Drowning	4	-
**UMB1349**		5	M	Autism	Caucasian	Repeated antibiotics due to ear infections	Fallen cervix during pregnancy; occasional cigarette use during pregnancy; no alcohol use; induced labor	None reported	Mother reported that father had: "mood swings"; Maternal male cousin had behavior problems and delayed speech.	Drowning	39	Notes indicated marked obesity, hypogonadism, and high height/weight percentile before age 1; neurologist wanted to check for chromosomal disorder.
**B1469**	AN13364	5	M	Autism	Caucasian	No information-TARF case	No information-TARF case	No information-TARF case	No information- TARF case	No information-TARF case	42.83	-
**UMB1174**		7	F	Autism			Nothing remarkable reported			Multi-symptom organ failure	14	Immediately preceding death had a sever respiratory infection
**UMB4849**		7	M	Autism	African-American	Inpatient chelation due to lead poisoning (plumbism) @ age 3	Full term, cesarean; heroine, cocaine, alcohol and cigarette use during pregnancy; child hospitalized with withdrawal for first 3 weeks of life	None reported	Younger female sibling with autism	Drowning	20	
**B5666**	AN19511	8	M	Autism		Depakote, Chemotherapy	Nothing remarkable reported		Paternal uncle “antisocial” with possible Aspergers; paternal and maternal cousin with”delays”;	Mestatic Rhabdomysarcoma (Cancer)	22.2	
**UMB4231**		8	M	Autism	African American		Nothing remarkable reported		None	Drowning	12	

						Zyprexa, Reminyl, Adderal						
**UMB4721**		8	M	Autism	African-American	None	Nothing remarkable reported		Brother with speech delay	Drowning	16	
**UMB1182**		9	F	Autism	African American	None reported	Nothing remarkable reported	None reported	None	Smoke inhalation	24	Child diagnosed with pica; psychiatrist recommended that certain medications be considered (Luvox or Prozac and<br><br>Ritalin or Dexedrine, but no record of child taking medications; no toxicity test at autopsy
**UMB797**		9	M	Autism	Caucasian	Desipramine (antidepressant) for ADD	Nothing remarkable reported	None reported	None	Drowning	13	Autopsy said child mistakenly given a double dose of med 1 week before death and was treated in the hospital; pm toxicology revealed high level in blood at time of death but about in the normal therapeutic level, but seizures and cardiac arrhythmia are reported complications of the med
**B4925**	AN16641	9	M	Autism		Fluvozamine, Tegretol, Ritalin, Clonodine, Prozac, Luvox, Fenfluramine, and possibly Risperdal	Nothing remarkable reported		One sister with seizures; one sister with speech delay and learning problems, paternal brother with history of social difficulties, hallucinations, and paranoia, and on	Seizure Disorder	27	

									psychotropic medication.			
**UMB4899**		14	M	Autism	Caucasian	Trileptal, Zoloft, Clonidine, Melatonin	Nothing remarkable reported		Fraternal twin brother with autism	Drowning	9	
**B7079**	AN04682	15	M	Autism						Asphyxia by hanging	23.23	Brain weight 1370
**B6337**	AN09730	22	M	Autism						Asphyxia	25	
**B6994**	AN08166	28	M	Autism	Caucasian	Seizure, antipsychotic and anxiety drug history including Geodon, Carbamazepine, Trileptal, methylphenidate, Buspar, Tenex, Lithium, Serzone, Effexor, Zyprexa, Risperdal, Stellazine, Clonodine, Abilify, Aricept, Amantadine, Ritalin, Seroquel , and allergy medications	Premature birth at 32 weeks gestation; birth complicated by pre-eclampsia in 6th month and toxemia in 8th month, diabetes, c-section, diabetes and HPB, performed as the donor was in breech position. Born at 10 lbs, ICU for 2, days to stabilize his blood sugar and received oxygen for respiratory distress	Y - Clomid	Mother has essential tremor, is anxious and cervical dystonia & spasmodic dystonia; Paternal nephew with behavior problems, aggression, difficulty with social interaction, impulsive behavior, learning problems and mood swings; Paternal grandfather with impulsive behavior; Paternal cousins with history of difficulty with social interactions, learning problems, mood swings, speech-language problems, and substance abuse.	Seizure disorder	43.25	Obesity
**B5173**	AN08792	30	M	Autism		Dilantin, Depakote, Tranxene, Cisapride, Clorazepate , Lactulose; Phenobarbital, and probable Trileptal	Labor induced with oxytocin	N	Maternal family history of Alzheimer's, Parkinson's, Depression, OCD, epilepsy; Mother diagnosed with OCD and had a Subependymoma brain tumor (which can reoccur) removed; father abused alcohol; father, brother, and paternal half-brother IQ over 140. Full brother normal with no delays.	Complications of gastrointestinal bleeding	20.3	Child had a number of medical issues; ; umbilical cord wrapped around neck 3 times at birth very tightly; odd involuntary movements noticed at 5 months of age, seizures began at around 7 months of age; Myoclonic seizures began later along with falling episodes requiring him to wear a helmet and mouth

												guard all the time; neurologist reported EEG quite abnormal with diffuse slowing and frequent spike discharges more prominent on the left; placed in walker at age 5 and lost ability to sit or stand unsupported by 12; self injury; feeding tube for seizure meds; never achieved bladder/bowel control; lived in nursing home; negative for fragile x
**B6677**	AN11989	30	M	Autism		Unidentified medication for continence at age 6; Colipromine, Zoloft, indentified medications for pain management of congestive heart failure; significant medical history including surgery for scoliosis, heart murmur, aortic insufficiency, brain surgery to remove tumor, seizures related to brain tumor	None reported	None reported	Mother reported she had difficulty with social interactions	Congestive heart failure	16	Positive history for seizures related to brain tumor; ; received all vaccinations
**UMB1445**		45	M	Autism	Caucasian	None reported	No information	None reported	Brother in residential facility, likely also autistic but diagnosis not reported	Complications of ALS / Autism	23	Reported to also have ALS (amyotrophic lateral sclerosis); lived in residential facility; based on social service notes seems to have one unaffected brother who visited him but could not provide

												the care he needed and one brother also in the residential facility;
**B7109**	AN17254	51	M	Autism				None reported	Paternal family history negative for disorders; maternal family history significant for diabetes, migraines, and SIDS; brother with dyslexia, other brother no difficulties	Cardiac arrest	22	History of hypoglycemia
**B4498**	AN01093	56	M	Autism	Caucasian	Lithonate, Cogentin, Haldol, Thorazine, Cogentin, Benadryl	Preterm cesarean; exact gestation not reported	None reported		Anoxic Encephalopathy	19.48	Developed Keratoconus (eye condition = legally blind); parents reported 15 words by age 15 months then regression at 18 months and use of augmentative communication (card w/ typewriter key) by age 7 and could spell out his needs
**Case #**	**Alternate ID**	**Age**	**Gender**	**Diagnosis**	**Race**	**Hx Meds**	**Prenatal Hx**	**IVF/ Fertility Hx**	**Psych DO in Family**	**Cause of Death**	**PMI**	**Other**
**UMB1185**		4	M	Control	Caucasian	Atropine and Epi at death scene to revive heart with no response	None reported	None reported	None reported	Drowning	17	drowned at beach; HIV negative
**UMB4670**		4	M	Control	Caucasian	None reported	None reported	None reported	None reported	Commotio Cordis	17	cardiac arrest immediate following baseball to sternum; HIV negative
**UMB1377**		6	F	Control	Caucasian	None reported	None reported	None reported	None reported	Drowning	20	child in 3ft inflatable pool face down; HIV negative
**UMB4898**		7	M	Control	Caucasian	Concerta and Clonodine				Drowning	12	Diagnosed with hyperactive disorder; premature gestation of 5-6

												months
**UMB1860**		8	M	Control	Caucasian	None reported	None reported	None reported	None reported	Cardiac Arrhythmia	5	Child had cardiac issues, diagnosed with abnormal coronary artery several days before he died; autopsy results indicated inflammatory myofibroblastic tumor of the aortic valve; HIV negative
**UMB1674**		8	M	Control	Caucasian	None reported	None reported	None reported	None reported	Hypothermia & Drowning	36	Child fell through ice in a park; remained under ice for 60 minutes; unresponsive to intubation HIV negative. (report says hyperthermia but I changed based on description)
**UMB1650**		10	M	Control	Caucasian	None reported	None reported	None reported	None reported	Sudden Unexplained Death in Childhood (SUDC)	24	Diagnosed with polymyosis (uncommon connective tissue disease with inflammation) and undergoing evaluation at time of death; HIV negative
**UMB1714**		12	M	Control	African American	None reported	None reported	None reported	None reported	Cardiac Arrthymia	22	history of high cholesterol and enlarged heart; HIV negative
**UMB4787**		12	M	Control	African American	Singulair, Albuterol, Prednisone, Claritin	None reported	None reported	None reported	Asthma	15	HIV negative, history of asthma and peanut & apple allergies
**UMB4722**		14	M	Control	Caucasian	None reported	None reported	None reported	None reported	Multiple injuries	16	ATV accident; non smoker; HIV negative

**UMB4638**		15	F	Control	Caucasian	None reported	None reported	None reported	None reported	Chest Injuries	5	passenger in car accident; HIV negative
**UMB1796**		16	M	Control	Caucasian	None reported	None reported	None reported	None reported	Multiple injuries	16	HIV Negative; passenger in car accident, went into cardiac arrest due to multiple injuries
**B6756**	AN07591	16	M	Control	Unknown	None reported	None reported	None reported	None reported	Heart attack	22	Brain weight 1230; history of. epilepsy
**B6207**	AN17425	16	M	Control	Unknown	None reported	None reported	None reported	None reported	Heart attack (secondary = heart disease	26.16	
**B5251**	AN03217	19	M	Control	Unknown	None reported	None reported	None reported	None reported	Pneumonia; secondary cause is respiratory infection	18.58	Brain weight 1555
**UMB1649**		20	M	Control	(Hispanic)	None reported	None reported	None reported	None reported	Multiple injuries	22	HIV negative
**BTB3960**		25	F	Control	Caucasian	None reported	None reported	None reported	None reported	Gunshot wound to chest	26	neuropath states "normal brain", brain weight is 1520 g
**UMB818**		27	M	Control	Caucasian	None reported	None reported	None reported	None reported	Multiple injuries	10	car accident (driver), open container (beer) in car
**B5873**	AN19760	28	M	Control	Unknown	None reported	None reported	None reported	None reported	Unknown	23.25	Brain weight 1580; no known disorder.
**B5334**	AN15622	30	M	Control	Unknown	None reported	None reported	None reported	None reported	Unknown	14.8	
**B5352**	AN12137	31	M	Control	Unknown	None reported	None reported	None reported	None reported	None reported	33	
**B5813**	AN01410	41	M	Control	Unknown	None reported	None reported	None reported	None reported	Unknown	27	

**B6208**	AN19440	50	F	Control	Unknown	None reported	None reported	None reported	None reported	Heart attack	20.25	Brain weight 1320
**B4756**	AN10606	56	M	Control	Unknown	None reported	None reported	None reported	None reported	Unknown	23	
**B6860**	AN13295	56	M	Control	Unknown	None reported	None reported	None reported	None reported	Unknown	22	
