# Supplementary material for: Age-Dependent Brain Gene Expression and Copy Number Anomalies in Autism Suggest Distinct Pathological Processes at Young Versus Mature Ages
Source: PLoS Genet. 2012 Mar 22;8(3):e1002592. doi: 10.1371/journal.pgen.1002592 (PMC3310790; doi:10.1371/journal.pgen.1002592)
Supplement: Table S2 — Differentially expressed genes in young autistic cases. All 102 differentially expressed genes in the posthoc comparison passing a threshold of p<0.05 between young autism and young control cases of genes showing an effect of interaction between diagnosis and categorical age. Gene symbols, Illumina probe IDs, p-Value of difference, cytogenetic bands and fold changes are listed. Bold genes are shown in Figure 1. (PDF) [file pgen.1002592.s006.pdf]

Supplementary Table 2: Differentially Expressed Genes of Young Autistic Cases					
**#**	**Probe ID**	**p-Value**	**Gene**	**Cytoband**	**Fold Change**
37	ILMN_1767556	0.0178242	C10orf10	10q11.21c	2.283901423
18	ILMN_1702296	0.0091403	CARTPT	5q13.2b	2.093626699
3	ILMN_2415157	0.0024493	ARID5A	2q11.2a	2.073438838
10	ILMN_1782050	0.0056193	CEBPD	8q11.21a	1.841181227
2	**ILMN_2286379**	**0.0017495**	**HYDIN**	**16q22.1f-q22.3a**	**1.769483623**
39	ILMN_1740426	0.0184694	RASD1	17p11.2g	1.747239056
34	**ILMN_1652185**	**0.0166762**	**IL4R**	**16p12.1a**	**1.74636822**
9	ILMN_1763587	0.0051548	TNMD	Xq22.1b	1.742366729
97	**ILMN_1721774**	**0.0470183**	**MPP7**	**10p11.23c**	**1.711712913**
14	ILMN_2409167	0.0075977	ANXA2	15q22.2a	1.692925858
65	**ILMN_1710514**	**0.0294053**	**BCL3**	**19q13.31b**	**1.669903233**
22	ILMN_1692332	0.0125032	ALOX12B	17p13.1c	1.657099882
87	ILMN_2391912	0.0393724	SEC14L1	17q25.2b	1.64442072
31	**ILMN_2282019**	**0.0164827**	**SYN3**	**22q12.3a**	**1.626937071**
59	ILMN_1761309	0.0266007	ADCK5	8q24.3h	1.595471013
76	ILMN_1694913	0.0340732	LMO3	12p12.3d	1.593570791
33	ILMN_2113728	0.0166613	FLJ31568	22q11.23a	1.580422773
12	**ILMN_2114422**	**0.0071293**	**NOD1**	**7p15.1b**	**1.554913981**
85	ILMN_1655713	0.0388536	OR2M7	1q44f	1.540474863
86	**ILMN_1653826**	**0.0389161**	**FGF13**	**Xq26.3d**	**1.538524147**
56	ILMN_2089616	0.0248697	FBXO10	9p13.2a	1.529865637
60	**ILMN_1773914**	**0.0266055**	**POU6F2**	**7p14.1d**	**1.525413123**
32	ILMN_2086612	0.0165765	CMAH	6p21.32	1.510486073
49	ILMN_1773271	0.0218208	SCN7A	2q24.3d	1.496558223
7	ILMN_1808810	0.0045008	CERKL	2q31.3b	1.492776302
38	**ILMN_1725175**	**0.0180504**	**FOSL2**	**2p23.2b**	**1.476985004**
95	ILMN_1664170	0.0452175	CUGBP2	10p14Bp14a	1.47148914
81	ILMN_1687495	0.0364861	SLC37A1	21q22.3b	1.461054692
54	ILMN_1753111	0.0233274	NAMPT	7q22.2c	1.453358726
67	ILMN_1811608	0.0296929	FLJ14107	8p21.3	1.443912079
82	**ILMN_2365111**	**0.0376773**	**MAP4K1**	**19q13.2a**	**1.443153441**
55	**ILMN_2319077**	**0.0243218**	**FAS**	**10q23.31b**	**1.429930875**
5	**ILMN_1803593**	**0.002886**	**WNT3**	**17q21.32a**	**1.418587714**
66	ILMN_1792094	0.0294248	TMEM63C	14q24.3c	1.418420387
75	**ILMN_1687967**	**0.0340483**	**NTRK3**	**15q25.3d**	**1.405410192**
90	ILMN_2351241	0.0404107	RNF14	5q31.3c	1.363125044
88	ILMN_1692742	0.0397669	DENND3	8q24.3c	1.34144919
96	**ILMN_1756705**	**0.0453123**	**CHTF18**	**16p13.3f**	**1.330898774**
**24**	**ILMN_1720181**	**0.0135588**	**CLSTN1**	**1p36.22d**	**1.32232156**
102	ILMN_1742968	0.0492937	NMNAT2	1q25.3d	1.31346127
44	**ILMN_1741465**	**0.0199732**	**PTPRH**	**19q13.42b**	**-1.361593416**
41	**ILMN_1695631**	**0.0190538**	**CHP2**	**16p12.1b**	**-1.415551164**
17	ILMN_1795673	0.0089112	LOC441476	9q34.3f	-1.418359605
64	ILMN_1815165	0.0293862	SLC28A1	15q25.3a	-1.420135748
**84**	**ILMN_1695036**	**0.0383753**	**TPPP3**	**16q22.1b**	**-1.421260234**
**1**	**ILMN_2178994**	**0.0016877**	**TUBB8**	**10p15.3d**	**-1.434781489**
77	ILMN_1775136	0.0347722	C3orf45	3p21.31b	-1.47778267
94	ILMN_1726210	0.0444314	GPIHBP1	8q24.3f	-1.502684371
36	ILMN_1785413	0.0174033	ATP2C2	16q24.1a	-1.517927532
35	ILMN_2277334	0.0167047	KCNK2	1q41a	-1.523586114
63	**ILMN_1712537**	**0.0279818**	**NODAL**	**10q22.1b**	**-1.537181567**
74	ILMN_1684742	0.0335874	TCEA2	20q13.33e	-1.547607636
50	ILMN_1754132	0.0220576	FLJ45079	17q25.3a	-1.550516956
57	**ILMN_1663390**	**0.0258167**	**CDC20**	**1p34.2a**	**-1.567522565**
69	ILMN_1673605	0.0303997	PRSSL1	19p13.3j	-1.579873391
52	ILMN_1665311	0.0231118	STH	17q21.31e	-1.580142072
98	ILMN_2102422	0.0470686	ABCB8	7q36.1d	-1.581219165
61	ILMN_1738539	0.0271168	OPLAH	8q24.3g	-1.586292139
91	**ILMN_2311089**	**0.0407431**	**BRCA1**	**17q21.31a**	**-1.603892207**
**15**	**ILMN_2106874**	**0.0079741**	**CACNG8**	**19q13.42a**	**-1.607977761**
8	**ILMN_2377980**	**0.004787**	**PPP1CA**	**11q13.1f**	**-1.621301223**
79	ILMN_1710312	0.0351176	TMEM31	Xq22.2a	-1.632386858
101	ILMN_1688646	0.0484697	LCN10	9q34.3e	-1.659630008
29	ILMN_1810092	0.0156641	TMEM179	14q32.33b	-1.659998524
62	ILMN_1667840	0.0277708	CMTM3	16q21e-q22.1a	-1.661106161

42	**ILMN_1739805**	**0.0194196**	**NDE1**	**16p13.11Bp13.11a**	**-1.669673154**
80	**ILMN_1759585**	**0.0359602**	**CHEK2**	**22q12.1c**	**-1.670873753**
46	**ILMN_1698252**	**0.0203824**	**FANCB**	**Xp22.2**	**-1.697438213**
23	**ILMN_2370588**	**0.0133007**	**TRIOBP**	**22q13.1a**	**-1.697645607**
99	ILMN_1727045	0.047567	RASGRP3	2p22.3d	-1.699373189
27	ILMN_1722070	0.0153361	APOC3	11q23.3b	-1.73837072
71	ILMN_1671362	0.0321787	UBE2J2	1p36.33a	-1.754267722
45	**ILMN_1684576**	**0.0203819**	**CLN8**	**8p23.3a**	**-1.755853888**
48	ILMN_1677108	0.0214474	CAPN13	2p23.1Bp23.1a	-1.78376345
93	**ILMN_2128931**	**0.0442292**	**FAT2**	**5q33.1d**	**-1.79130078**
30	**ILMN_2238795**	**0.0156762**	**NEK3**	**13q14.3d**	**-1.802075509**
40	ILMN_2111932	0.0190218	SERINC2	1p35.2a	-1.819803173
78	ILMN_1698060	0.0349873	FLJ43860	8q24.3d	-1.832144494
**28**	**ILMN_2293131**	**0.0155328**	**ARHGEF1**	**19q13.2c**	**-1.838419525**
**83**	**ILMN_2349982**	**0.0378258**	**FGF1**	**5q31.3d**	**-1.873113689**
26	ILMN_1772370	0.0152546	ARHGEF1	19q13.2c	-1.879996133
89	**ILMN_1661875**	**0.0400691**	**ANK3**	**10q21.2a**	**-1.92792007**
**53**	**ILMN_1750941**	**0.0232784**	**DLX4**	**17q21.33a**	**-1.93072079**
100	ILMN_1761965	0.0476763	ZNF200	16p13.3c	-1.940928777
70	ILMN_1775348	0.0308159	KCNH8	3p24.3c	-2.014486404
68	**ILMN_1790106**	**0.0303653**	**PLP1**	**Xq22.2a**	**-2.017485029**
4	**ILMN_1776936**	**0.0026995**	**ANKRD38**	**1p31.3d**	**-2.023986121**
73	ILMN_1803850	0.0333949	TSPAN15	10q21.3e	-2.068264427
**25**	**ILMN_2208777**	**0.014313**	**NHLH2**	**1p13.1d**	**-2.068656311**
47	ILMN_1795300	0.0208269	FLJ41603	5q33.1b	-2.070391027
**6**	**ILMN_2238389**	**0.004419**	**LDB3**	**10q23.2a**	**-2.09188599**
58	ILMN_1672443	0.026461	QDPR	4p15.32b	-2.13894527
**51**	**ILMN_2175715**	**0.0229608**	**KIR2DS3**	**19q13.4**	**-2.295779916**
13	**ILMN_2124585**	**0.007413**	**GREM1**	**15q13.3c-q13.3d**	**-2.305262746**
**92**	**ILMN_1756807**	**0.0429335**	**GPR62**	**3p21.1e**	**-2.435674441**
72	ILMN_1751785	0.0326038	DMRT2	9p24.3b	-2.586787043
**21**	**ILMN_1746646**	**0.0121327**	**CHRM5**	**15q14a**	**-2.656677881**
16	ILMN_1720727	0.0084141	SLCO1A2	12p12.1e	-2.765890212
19	**ILMN_1777342**	**0.0105735**	**PREX1**	**20q13.13b**	**-2.770779816**
11	**ILMN_1781356**	**0.005856**	**TSC22D3**	**Xq22.3b**	**-2.993190062**
20	**ILMN_1717381**	**0.0116866**	**HOXD1**	**2q31.1h**	**-3.063724889**
43	**ILMN_1676384**	**0.0196882**	**PCSK6**	**15q26.3d**	**-3.094558732**
