# Supplementary material for: Age-Dependent Brain Gene Expression and Copy Number Anomalies in Autism Suggest Distinct Pathological Processes at Young Versus Mature Ages
Source: PLoS Genet. 2012 Mar 22;8(3):e1002592. doi: 10.1371/journal.pgen.1002592 (PMC3310790; doi:10.1371/journal.pgen.1002592)
Supplement: Table S3 — Dysregulated genes of young autistic cases and previously identified candidate genes and loci. Loci of 102 differentially expressed genes in the posthoc comparison and references of previous linkage/association/CNV studies implicating the candidate locus and references of other genes neighboring the candidate loci are listed. NEGATIVE = negative association/linkage. (PDF) [file pgen.1002592.s007.pdf]

Supplementary Table 3: Dysregulated genes of young autistic cases and previously identified candidate genes and loci				
Gene Symbol	Probe ID	Cytoband	Locus Linkage/ Association/CNV	Genes near locus
C10orf10	ILMN_1767556	10q11.21c	Trikalinos et al., 2006	
RASD1	ILMN_1740426	17p11.2g	Trikalinos et al., 2006	OMG on 17q11.2 (Martin et al., 2007); SLC6A4 (Cook 1997, Yirmiya et al., 2001, Kim et al., 2002 and Sutcliffe et al., 2005)
IL4R	ILMN_1652185	16p12.1a	Risch et al., 1999; Yang and Gill, 2007; Kumar and Christian, 2009	GRIN2A and ABAT are on 16p11-13 (Barnby et al., 2005)
MPP7	ILMN_1721774	10p11.23c	Lauritsen et al., 2006	
ALOX12B	ILMN_1692332	17p13.1c	Risch et al., 1999	
SYN3	ILMN_2282019	22q12.3a	IMGSAC 1998	
FLJ31568	ILMN_2113728	22q11.23a	Itsara et al., 2009	
NOD1	ILMN_2114422	7p15.1b		HOXA1 is on 7p15.3 (POSITIVE: Ingram et al., 2000; NEGATIVE: Li et al., 2002)
OR2M7	ILMN_1655713	1q44f	Risch et al., 1999	
POU6F2	ILMN_1773914	7p14.1d	Anney et al., 2010	
CMAH	ILMN_2086612	6p21.32		HLA-A is on 6p21.3 (Torres 2006); HLA-DRB1 (Torres 2002* and Warren 1996); GRIK2 (Jamain 2002 and Shuang 2004); GLO1 (Junaid 2004); NEGATIVE: HLA (Stubbs 1980)
SCN7A	ILMN_1773271	2q24.3d	Philippe et al., 1999; Segurado et al., 2005	NEGATIVE: CMYA3 2q24.3 (Blasi 2006); POSITIVE SLC25A12 is on 2q24 (Freitag 2007, Silverman 2008)
CERKL	ILMN_1808810	2q31.3b	IMGSAC 2001; Lauritsen et al., 2006	
FOSL2	ILMN_1725175	2p23.2b	Ronald et al., 2009	
CUGBP2	ILMN_1664170	10p14b-p14a	Buxbaum 2004, Risch et al., 1999	
NAMPT	ILMN_1753111	7q22.2c	Trikalinos et al., 2006	FOXP2, RELN, PTPRZ1, NRCAM, WNT2, HOXA1 are on 7q22-36 (Persico et al., 2001; Serajee et al., 2006; Skaar et al., 2005; Freitag et al., 2010)
FLJ14107	ILMN_1811608	8p21.3	IMGSAC 1998	
MAP4K1	ILMN_2365111	19q13.2a		NEGATIVE: APOE on 19q13.2 (Raiford 2004)
FAS	ILMN_2319077	10q23.31b	Buxbaum et al., 2004	PTEN is on 10q23.31 (Butler 2005; Herman et al., 2007)
WNT3	ILMN_1803593	17q21.32a	Cantor et al., 2005; Kumar and Christian 2009	

TMEM63C	ILMN_1792094	14q24.3c	Lauritsen et al., 2006	
NTRK3	ILMN_1687967	15q25.3d	Szatmari et al., 2007	
CHTF18	ILMN_1756705	16p13.3f	Kumar and Christian 2009; Risch et al.,1999; Lauritsen et al.,2006	
CHP2	ILMN_1695631	16p12.1b	Risch et al., 1999; Yang and Gill, 2007; Kumar and Christian, 2009	
SLC28A1	ILMN_1815165	15q25.3a	Szatmari et al., 2007	
C3orf45	ILMN_1775136	3p21.31b	Ylisaukko-oja et al., 2006; Rehnstrom et al., 2006; Stuart et al., 2007	
KCNK2	ILMN_2277334	1q41a	Maussion et al., 2008	
NODAL	ILMN_1712537	10q22.1b		EGR2 is on 10q21.2-q22.1 (Swanberg et al., 2009)
PRSSL1	ILMN_1673605	19p13.3j	Philippe et al., 1999	
STH	ILMN_1665311	17q21.31e	Cantor et al., 2005; Kumar and Christian 2009	
ABCB8	ILMN_2102422	7q36.1d	Molloy et al., 2005	CNTNAP2 7q26-q36 (Arking 2008; Bakkaloglu et al., 2008), EN2 is on 7q36 (Brune 2008; Freitag 2007; Wang 2008)
BRCA1	ILMN_2311089	17q21.31a	Cantor et al., 2005; Kumar and Christian 2009	ITGB3 and HOXB1 on 17q21.3 (Ingram 2000)
LCN10	ILMN_1688646	9q34.3e		DBH on 9q34 (Robinson et al., 2001)
NDE1	ILMN_1739805	16p13.11b-	Kumar and Christian, 2009	
TRIOBP	ILMN_2370588	22q13.1a	Durand et al., 2007	SHANK3 is on 22q13.3 (Moessner et al., 2007)
DLX4	ILMN_1750941	17q21.33a	Cantor et al., 2005; Kumar and Christian, 2009	
ZNF200	ILMN_1761965	16p13.3c	Kumar and Christian, 2009; Risch et al.,1999; Lauritsen et al., 2006	TSC2 is on 16p13.3 (Kumar and Christian, 2009)
KCNH8	ILMN_1775348	3p24.3c		OXTR is on 3p24-26 (Jacob 2007; Lerer 2007; Wu 2005)
NHLH2	ILMN_2208777	1p13.1d	Risch et al., 1999; Auranen et al 2002, 2000	
QDPR	ILMN_1672443	4p15.32b		DRD5 on 4p16.1-p15.3 (Philippe et al 2002)
GREM1	ILMN_2124585	15q13.3c-q13.3d	Miller et al., 2008; Pagnamenta et al., 2008; Ben-Shacher et al., 2009	
GPR62	ILMN_1756807	3p21.1e	Ylisaukko-oja et al., 2004; Rehnstrom et al., 2006	

CHRM5	ILMN_1746646	15q14a	Lauritsen et al., 2006	
SLCO1A2	ILMN_1720727	12p12.1e	McCauley et al., 2005	
TSC22D3	ILMN_1781356	Xq22.3b	Jamain et al., 2003	NLGN4 is on Xp22.33 (Jamain et al., 2003)
HOXD1	ILMN_1717381	2q31.1h	IMGSAC 2001; Lauritsen et al., 2006	NEGATIVE: 2q21-q33 (Bacchelli 2003) including DLX2, TBR1, GAD1, DLX1, RAPGEF, CHN1, ATF2, HOXD1, NEUROD1
