# Supplementary material for: Age-Dependent Brain Gene Expression and Copy Number Anomalies in Autism Suggest Distinct Pathological Processes at Young Versus Mature Ages
Source: PLoS Genet. 2012 Mar 22;8(3):e1002592. doi: 10.1371/journal.pgen.1002592 (PMC3310790; doi:10.1371/journal.pgen.1002592)
Supplement: Table S4 — Top downregulated and upregulated genes in adult autistic cases. Genes showing greatest fold change difference in the posthoc comparison passing a threshold of p<0.05 between adult autism and adult control cases of genes showing an effect of interaction between diagnosis and categorical age. Gene symbols, Illumina probe IDs, p-Value of difference, cytogenetic bands and fold changes are listed. (PDF) [file pgen.1002592.s008.pdf]

Supplementary Table 4: Top downregulated and upregulated genes of adult autistic cases					
**#**	**Probe ID**	**p-Value**	**Symbol**	**Cytoband**	**Fold Change**
20	ILMN_1731958	0.0003479	SYT10	12p11.1b	-2.730056126
428	ILMN_1753005	0.0135952	RELN	7q22.1g	-2.698836674
687	ILMN_1715505	0.0414112	TTC6	14q21.1a	-2.680432024
604	ILMN_1736238	0.0297894	GNMT	6p21.1d	-2.65171187
236	ILMN_2355549	0.0054637	GSTT2	22q11.23a-	-2.644603509
350	ILMN_1813607	0.0090789	ANKRD30A	10p11.21a	-2.519140966
27	ILMN_1757639	0.0003921	C3orf57	3q26.1b	-2.51071156
61	ILMN_1799980	0.0009225	LOC220115		-2.450734642
273	ILMN_1730256	0.0063925	GNG13	16p13.3f	-2.415651692
295	ILMN_2176625	0.007156	LOC285735	6q23.2c	-2.336855447
131	ILMN_1797816	0.0023888	KIAA1303	17q25.3e	-2.314081652
42	ILMN_1811048	0.0006414	GPR107	9q34.11d-	-2.311350685
4	ILMN_1723398	0.0000261	CSNK1G1	15q22.31a	-2.30997356
496	ILMN_2081465	0.0192068	APLP2	11q24.3c	-2.292577492
376	ILMN_2393149	0.0102471	ALOX15B	17p13.1d	-2.273181748
121	ILMN_1712419	0.0022239	DCDC2	6p22.2c-	-2.266754793
347	ILMN_1749846	0.0090397	OMD	9q22.31a	-2.257742223
217	ILMN_1719309	0.0046324	LRRC39	1p21.2a	-2.236685403
25	ILMN_1727909	0.0003856	CNTN5	11q22.1b-	-2.235990212
58	ILMN_1810053	0.0008772	SLC16A7	12q14.1b	-2.192796201
243	ILMN_2406035	0.0057495	LAMA3	18q11.2b-	-2.184068113
351	ILMN_1750044	0.0090995	ZNHIT3	17q12b	-2.181620396
72	ILMN_2386040	0.0011519	MYO19	17q12b	-2.175203764
202	ILMN_1695093	0.0042731	SLC7A8	14q11.2f	-2.156862339
264	ILMN_1786239	0.0062313	TACR1	2p13.1a	-2.152921371
2	ILMN_1667362	0.0000194	GABRB2	5q34a	-2.141128966
31	ILMN_1786254	0.0004513	DLGAP4	20q11.23a	-2.124198134
116	ILMN_1748083	0.0021464	RASGRF1	15q25.1a	-2.09927096
37	ILMN_1653728	0.0005512	ERBB4	2q34c-q34e	-2.093557826
258	ILMN_2397294	0.0060534	PLCB4	20p12.2b	-2.091533951
532	ILMN_2340919	0.0222906	GRB10	7p12.2a	-2.083989163
10	ILMN_1733564	0.000147	GPLD1	6p22.2b	-2.083641179
5	ILMN_2282366	0.0000475	IQSEC3	12p13.33d	-2.073151213
434	ILMN_1805131	0.0139148	C17orf90	17q25.3f	-2.064650058
105	ILMN_2246382	0.0018601	MAGED1	Xp11.22b	-2.060191098
366	ILMN_1658206	0.0098383	C1orf102	1p34.3d	-2.049788892
89	ILMN_1756443	0.0014781	INHA	2q35f	-2.034228764
404	ILMN_2211483	0.0119833	MAEL	1q24.1c	-2.01396759
187	ILMN_1679290	0.0038203	LTK	15q15.1c	-2.011554319
226	ILMN_1688843	0.0049296	GRK4	4p16.3a	-2.00422234
238	ILMN_1677104	0.0055164	TRAF5	1q32.3a	-1.999025173
393	ILMN_2259633	0.0111892	MLL5	7q22.2a	-1.990349299
28	ILMN_1705396	0.0003973	PIK3CA	3q26.32c	-1.988389774
81	ILMN_2334322	0.0013385	MIER1	1p31.3a	-1.98564354
348	ILMN_1768391	0.0090666	ARL4C	2q37.1e	-1.985565677
146	ILMN_2348194	0.0027697	OLA1	2q31.1f	-1.984668824
394	ILMN_1711681	0.0112681	SPATS1	6p21.1b	-1.983065722
48	ILMN_1675454	0.0006883	CDH9	5p14.1c	-1.96926315
107	ILMN_1782032	0.0018768	ASH1L	1q22a-q22b	-1.951597338
408	ILMN_1728714	0.012227	SSSCA1	11q13.1c	-1.947847512
43	ILMN_2285648	0.0006455	KCNIP4	4p15.31d-	-1.945641418
469	ILMN_1726392	0.0167632	NIN	14q22.1b-	-1.945394588
531	ILMN_1733541	0.0222792	GPR123	10q26.3f	-1.940560717
398	ILMN_2193761	0.0114331	LOC442245		-1.934364419
364	ILMN_2391176	0.009805	STAR	8p12a	-1.927782991
167	ILMN_1741143	0.0032139	TXK	4p12a	-1.926998269
142	ILMN_1683752	0.0027166	LHX6	9q33.2b	-1.91859797
68	ILMN_2146495	0.0010823	GPR153	1p36.31a	-1.915121627
487	ILMN_2391512	0.0184667	ASAHL	4q21.1a	-1.912485356
173	ILMN_1677887	0.0034115	PMS2		-1.911807915
129	ILMN_1793347	0.0023755	KIAA1529	9q22.33a	-1.910805021
138	ILMN_2254583	0.0025829	SNX14	6q14.3c	-1.903550686
290	ILMN_2398995	0.0070144	MRPL24	1q23.1a	-1.901466172
253	ILMN_2277077	0.0059395	PRKAR1A	17q24.2c	-1.900331278
585	ILMN_2341132	0.0280251	TNNT2	1q32.1c	-1.896070124

296	ILMN_1719616	0.0071676	DNASE1	16p13.3c	-1.892763018
45	ILMN_2363450	0.0006628	NOXO1	16p13.3e	-1.889079265
237	ILMN_1726107	0.0054822	UBE2V1	20q13.13e	-1.888565254
154	ILMN_1656840	0.0030177	VPS13D	1p36.22a	-1.882562208
136	ILMN_1753597	0.0025542	FKRP	19q13.32b	-1.882078442
179	ILMN_1677610	0.0035561	OVCA2		-1.880744657
405	ILMN_2366205	0.0120498	CACNB1	17q12c	-1.877961266
340	ILMN_1689899	0.0086154	MTERFD2	2q37.3f	-1.876189905
501	ILMN_1776850	0.0196462	ITGB1BP3	19p13.3e	-1.873415612
197	ILMN_1718971	0.0040133	TTC7B	14q32.11a	-1.866143041
86	ILMN_1787513	0.0014274	ACTR3B	7q36.1e	-1.857312441
216	ILMN_1779386	0.004623	RASGRF1	15q25.1a	-1.856020487
84	ILMN_1766366	0.0013785	ICA1L	2q33.1g	-1.853009525
328	ILMN_2211122	0.0081799	SEDLP		-1.85045701
50	ILMN_1680757	0.0007145	LRRC26	9q34.3e	-1.844116734
76	ILMN_2296644	0.0012424	PCDHGA9	5q31.3c	-1.840520028
212	ILMN_1678391	0.0045444	SPEF2	5p13.2c	-1.839149728
7	ILMN_1795756	0.0000666	CNKSR2	Xp22.12a	-1.832325097
215	ILMN_1801408	0.004586	GABRA4	4p12b	-1.828721857
144	ILMN_2363805	0.0027549	YAF2	12q12e	-1.828456423
369	ILMN_2145708	0.0099773	NPAL2	8q22.2a	-1.824643576
280	ILMN_1692020	0.0066386	PRSS16	6p22.1c	-1.817867017
119	ILMN_2161820	0.0021787	GLYATL2	11q12.1c	-1.817863644
186	ILMN_1694823	0.0037752	PYDC1	16p11.2c	-1.817004706
196	ILMN_1676626	0.0040053	ARID4A	14q23.1b	-1.81423539
319	ILMN_2059996	0.0078656	MEGF8	19q13.2c	-1.807428704
447	ILMN_1776631	0.014853	KIAA1862	7q36.1b	-1.806265038
370	ILMN_1789692	0.0100095	MARK2	11q13.1a	-1.800040961
1	ILMN_1765518	0.0000099	RNASEH2C	11q13.1d	-1.797630225
161	ILMN_2321648	0.0031505	SH2D3C	9q34.11a	-1.795888022
102	ILMN_1758871	0.0018089	DAB1	1p32.2b-	-1.792076548
15	ILMN_2351754	0.000216	ADAM22	7q21.12b	-1.791300726
127	ILMN_2242533	0.0023391	FAIM	3q22.3c	-1.78569189
429	ILMN_1673455	0.0136079	RASAL2	1q25.2b	-1.782953233
681	ILMN_1712608	0.0402171	BFSP1	20p12.1a	-1.780303759
**#**	**Probe ID**	**p-Value**	**Symbol**	**Cytoband**	**Fold Change**
575	ILMN_1690993	0.0265037	NEUROG2	4q25f	2.110366626
729	ILMN_2274586	0.0478994	PKD1L2	16q23.2b	2.118376589
342	ILMN_1670767	0.0087638	LPO	17q22d	2.118813455
204	ILMN_1802360	0.0042837	KRTAP5-2	11p15.5b	2.120914902
241	ILMN_1733298	0.0056912	C1orf158	1p36.21d	2.129208656
571	ILMN_1677497	0.0262563	STAB2	12q23.3a	2.132389307
545	ILMN_1683492	0.0235903	GPR172B	17p13.2b	2.132858102
477	ILMN_2383124	0.0176078	CYP2D6	22q13.2b	2.139395268
607	ILMN_2320932	0.0299752	PAX3	2q36.1b	2.141778981
29	ILMN_1701881	0.0004285	ZNF365	10q21.2b	2.142510279
466	ILMN_1731525	0.0165927	GJB3	1p34.3f	2.143497102
416	ILMN_2294542	0.0126515	IL17RD	3p14.3b	2.147038974
335	ILMN_2173291	0.0084948	CYP4B1	1p33d	2.152494028
38	ILMN_2224765	0.0005634	TMEM75	8q24.21b	2.155406085
281	ILMN_1752995	0.0067415	FAM83A	8q24.13b	2.159132555
35	ILMN_2321150	0.0005151	GSTTP2		2.164403325
463	ILMN_1739440	0.0161493	VPS16	20p13c	2.166587797
46	ILMN_1684866	0.0006686	C21orf49		2.175351789
491	ILMN_1728972	0.0189685	FAM64A	17p13.2a	2.192711039
67	ILMN_1749234	0.0010676	MGC34774	7q21.11a	2.196125251
163	ILMN_2103107	0.0031782	ADAMDEC1	8p21.2d	2.212384972
382	ILMN_1794112	0.0104556	C3orf56	3q21.3a	2.225315811
11	ILMN_1700703	0.0001598	GADL1	3p24.1a-p23c	2.226444943
160	ILMN_1762142	0.0031374	CDCA7	2q31.1e	2.228033028
306	ILMN_2173719	0.0074067	DEFB114	6p12.3b	2.228137266
270	ILMN_1733531	0.0063667	C20orf134	20q11.22a	2.237253229
36	ILMN_2215881	0.0005361	ARHGAP11B	15q13.2b	2.247283036
503	ILMN_1750508	0.0197464	STMN1	1p36.11b	2.248861001
380	ILMN_1809676	0.0104089	CACNG6	19q13.42a	2.249456814
378	ILMN_2406656	0.0103751	GATA3	10p14d	2.252084415
256	ILMN_1802735	0.006019	FFAR3	19q13.12a	2.254687928

209	ILMN_1723443	0.0044722	LRP2	2q31.1a	2.254791494
112	ILMN_2203149	0.0020283	TMPRSS12	12q13.13b	2.259374315
269	ILMN_1670672	0.0063665	TMEM37	2q14.2b	2.271133241
26	ILMN_2283245	0.0003905	C9orf84	9q31.3b	2.278488583
170	ILMN_2096442	0.0032791	LOC260339		2.289283484
494	ILMN_1664267	0.0191902	WBSCR28	7q11.23b	2.289375672
53	ILMN_2090004	0.0007641	AQP10	1q21.3e	2.312584177
666	ILMN_1715612	0.0372906	OTOP2	17q25.1c	2.31853824
221	ILMN_1708267	0.0047071	CLDN15	7q22.1d	2.349077908
122	ILMN_1808388	0.002225	FOXI2	10q26.2c	2.351667206
149	ILMN_1728707	0.0028606	COLQ	3p24.3e	2.360840761
74	ILMN_2376152	0.0011893	METT11D1	14q11.2c	2.361134087
407	ILMN_2167961	0.0121899	FLJ42133	20q11.23b	2.365713576
402	ILMN_1665686	0.0117595	FAM38B	18p11.22a	2.36619872
110	ILMN_1758066	0.0020113	DSCR8	21q22.13b	2.371778547
182	ILMN_1795464	0.0036703	LTA	6p21.33a	2.372399539
672	ILMN_2175715	0.0385757	KIR2DS3		2.388833123
231	ILMN_1762713	0.0053286	C19orf59	19p13.2e	2.396810776
108	ILMN_1711346	0.0019508	EGFLAM	5p13.2a-	2.397376268
374	ILMN_1709177	0.0101663	ITIH5L	Xp11.21a	2.402538988
653	ILMN_1711032	0.0353797	IFITM5	11p15.5d	2.425044302
582	ILMN_1693749	0.0276913	BMP4	14q22.2b	2.429929417
417	ILMN_2337928	0.0126861	CXCR5	11q23.3e	2.442366075
479	ILMN_1768099	0.017686	SCGB1C1	11p15.5d	2.446051021
152	ILMN_1691606	0.0029068	LYG2	2q11.2c	2.446776169
126	ILMN_1782741	0.002327	CD300LB	17q25.1b	2.448088175
114	ILMN_2209088	0.0020921	KRTAP9-4	17q21.2a	2.455444626
225	ILMN_1777541	0.00492	KLK6	19q13.33d	2.4588339
260	ILMN_1792404	0.0061034	TM4SF4	3q25.1a	2.475633006
134	ILMN_2185866	0.00247	PCDHB17		2.477277009
413	ILMN_1661454	0.0124882	DGKA	12q13.2c	2.483148951
635	ILMN_1717381	0.0326863	HOXD1	2q31.1h	2.488961641
223	ILMN_2329290	0.0048037	TRIM10	6p21.33b	2.493164446
14	ILMN_2415329	0.0002014	SULT1C2	2q12.3c	2.5106449
66	ILMN_2143427	0.0010069	GLULD1	6q12a	2.518958424
12	ILMN_1707945	0.0001791	HELB	12q14.3b	2.53311269
437	ILMN_2277426	0.014103	LCK	1p35.1b	2.546365671
360	ILMN_2362809	0.0095269	TCL6	14q32.13b	2.549057534
391	ILMN_2170157	0.0110775	VCY	Yq11.221a	2.556831302
694	ILMN_1670302	0.0425944	HK3	5q35.2d	2.570257586
203	ILMN_2149292	0.0042784	TMEM40	3p25.1d	2.572981807
141	ILMN_2253145	0.0026814	NEK3	13q14.3d	2.576860748
341	ILMN_1687002	0.0087252	OSR2	8q22.2a	2.578091595
87	ILMN_1796316	0.0014442	MMP9	20q13.12b	2.578346331
3	ILMN_1694400	0.0000224	MSR1	8p22c	2.580374218
16	ILMN_1695135	0.0002183	GCM1	6p12.1d	2.647001195
298	ILMN_1759087	0.0071881	OR10V1	11q12.1d	2.659067089
113	ILMN_1711899	0.0020574	ANXA2	15q22.2a	2.669004215
34	ILMN_1744295	0.0004869	CDKN2A	9p21.3c	2.682000515
78	ILMN_2233334	0.0012525	MYH16		2.685025506
73	ILMN_1751164	0.0011618	ARHGAP30	1q23.3a	2.690895066
233	ILMN_1758888	0.0053477	PADI3	1p36.13e	2.692602216
96	ILMN_1748349	0.0016546	OR10G2	14q11.2d	2.711349703
41	ILMN_1815388	0.0006276	CLDN17	21q21.3c	2.728987478
586	ILMN_1730817	0.0281593	ING1	13q34a	2.74637199
414	ILMN_1717492	0.012496	GHSR	3q26.31a	2.770418605
275	ILMN_2068498	0.0064163	POU5F1P1		2.873951951
51	ILMN_1803218	0.0007543	GPR139	16p12.3a	2.886282852
75	ILMN_2188247	0.001212	IL17F	6p12.2a	2.898159247
456	ILMN_1791587	0.0153475	OR2Y1	5q35.3f	2.97215162
82	ILMN_2298464	0.0013666	MOBP	3p22.1c	2.998003085
148	ILMN_1787212	0.0028366	CDKN1A	6p21.31a	3.010425564
140	ILMN_2278413	0.0026473	VCX2	Xp22.31c	3.088647362
6	ILMN_2283001	0.0000611	DSE	6q22.1d	3.114125013
242	ILMN_2203753	0.0057353	VENTXP7		3.164731664
145	ILMN_1811289	0.00276	COL4A6	Xq22.3c	3.350160307
139	ILMN_1653073	0.0026214	OR10Q1	11q12.1b	3.361394615

327	ILMN_2391071	0.0081652	CSH2	17q23.3b	3.364312148
274	ILMN_1744432	0.0064048	COX8C	14q32.13a	3.504065795
410	ILMN_2295559	0.0122545	A3GALT2	1p35.1a	4.499833268
