# Supplementary material for: Age-Dependent Brain Gene Expression and Copy Number Anomalies in Autism Suggest Distinct Pathological Processes at Young Versus Mature Ages
Source: PLoS Genet. 2012 Mar 22;8(3):e1002592. doi: 10.1371/journal.pgen.1002592 (PMC3310790; doi:10.1371/journal.pgen.1002592)
Supplement: Table S5 — Top downregulated and upregulated genes of male autistic cases across all young and older ages (2 to 56 years). Genes showing greatest fold change differences and a main effect of diagnosis passing a threshold of p<0.05 between autism and control cases, Illumina probe IDs, p-value of difference, cytogenetic bands and fold changes are listed. (PDF) [file pgen.1002592.s009.pdf]

Supplementary Table 5: Top upregulated and downregulated genes of autistic cases across young and older ages (2-56 years)					
#	Unique id	P-value	Symbol	Cytoband	Fold Change
184	ILMN_2208903	0.0024303	CD52	1p36.11b	-2.410674105
152	ILMN_2052079	0.0020735	ZNF544	19q13.43c	-2.143945694
1773	ILMN_1730256	0.0413185	GNG13	16p13.3f	-2.082915908
641	ILMN_2381020	0.0103796	SLCO1A2	12p12.1e	-2.048057703
1727	ILMN_1767393	0.0395188	GRIA3	Xq25b	-2.023978355
492	ILMN_2393149	0.0078457	ALOX15B	17p13.1d	-2.015891746
343	ILMN_2379823	0.0049367	TNFRSF19	13q12.12a	-2.012101301
422	ILMN_2176625	0.0064175	LOC285735	6q23.2c	-1.992223017
443	ILMN_1757639	0.0067223	C3orf57	3q26.1b	-1.955654811
117	ILMN_1731958	0.0015902	SYT10	12p11.1b	-1.915423598
136	ILMN_1743187	0.0019092	C6orf120	6q27f	-1.912032862
119	ILMN_1663062	0.001667	CCDC67	11q21a	-1.868389293
255	ILMN_2415447	0.0035365	KCNQ4	1p34.2c	-1.815448225
1687	ILMN_1746646	0.0381878	CHRM5	15q14a	-1.766627349
1455	ILMN_1781356	0.0302485	TSC22D3	Xq22.3b	-1.763821158
1135	ILMN_1753005	0.0214601	RELN	7q22.1g	-1.753085136
1034	ILMN_2118773	0.0193053	ASAH2B	10q11.23b	-1.741433805
114	ILMN_2123557	0.0015271	FAM73A	1p31.1e	-1.721109387
958	ILMN_2043569	0.0168192	MOSPD2	Xp22.2	-1.7130517
191	ILMN_1765257	0.0025085	CINP	14q32.31c	-1.703683781
337	ILMN_1651617	0.0048523	CTNNB1	3p22.1b	-1.691706334
1649	ILMN_1786239	0.0369945	TACR1	2p13.1a	-1.689192389
710	ILMN_1732189	0.0120642	SYCE1	10q26.3f	-1.685862436
330	ILMN_2162603	0.0047823	SMPX	Xp22.12a	-1.684780938
654	ILMN_1747129	0.010571	CASP8AP2	6q15d	-1.677791686
618	ILMN_2054652	0.0100063	TYRP1	9p23b	-1.675826976
24	ILMN_1730917	0.0004379	KMO	1q43e	-1.667494409
105	ILMN_1733564	0.0014416	GPLD1	6p22.2b	-1.651537764
761	ILMN_1719309	0.0129776	LRRC39	1p21.2a	-1.648665058
110	ILMN_1787408	0.0014958	NUDT17	1q21.1b	-1.647011505
1485	ILMN_2049184	0.0315735	DNASE1L3	3p14.3a	-1.641646708
120	ILMN_2296011	0.0016837	BRWD1	21q22.2a-q22.2b	-1.641608276
341	ILMN_1738672	0.0049123	TRIM33	1p13.2Bp13.2a	-1.639341873
1618	ILMN_2342835	0.0360879	P2RY14	3q25.1Bq25.1c	-1.636370025
62	ILMN_1678435	0.0009251	SRD5A3	4q12d	-1.632757655
118	ILMN_1653728	0.0016093	ERBB4	2q34c-q34e	-1.630507485
590	ILMN_2189815	0.009477	SLC22A10	11q12.3b	-1.628676424
737	ILMN_1713161	0.0124575	USP16	21q21.3c	-1.628597753
1072	ILMN_1752457	0.0200432	DDX4	5q11.2e	-1.626834268
87	ILMN_2262543	0.0012252	C20orf7	20p12.1d	-1.624474811
104	ILMN_2388716	0.0014371	KCNC4	1p13.3a	-1.621522673
1036	ILMN_2405756	0.0193171	VAMP1	12p13.31d	-1.621206674
1574	ILMN_2258409	0.0344905	P2RY14	3q25.1Bq25.1c	-1.617849604
553	ILMN_2082109	0.0089075	ZNF214	11p15.4b	-1.61759371
1140	ILMN_1773485	0.0216142	QKI	6q26c	-1.617285558
434	ILMN_1675053	0.0065985	DMN	15q26.3b	-1.616979002
1862	ILMN_1665714	0.0444812	C4orf22	4q21.21c	-1.609701068
1546	ILMN_1783131	0.0335151	SAMD13	1p31.1a	-1.607617581
1223	ILMN_1679837	0.0236763	SGPP1	14q23.2b	-1.59889703
1577	ILMN_1670193	0.0345835	ACY3	11q13.2a	-1.598672992
66	ILMN_2293012	0.0009626	ADAM32	8p11.23Bp11.23a	-1.590280446
1081	ILMN_2225104	0.020231	ZNF569	19q13.12c	-1.586342253
1651	ILMN_2092232	0.0370329	TSR1	17p13.3c	-1.584913022
584	ILMN_2382974	0.0093547	CCDC7	10p11.22b	-1.583323003
1988	ILMN_1659753	0.0490985	LAMP2	Xq24d	-1.583147012
333	ILMN_2147133	0.0048134	NBPF15	1q21.1d	-1.580338152
79	ILMN_2282366	0.0011305	IQSEC3	12p13.33d	-1.579910353
1809	ILMN_2071737	0.0427711	EIF5A2	3q26.2c	-1.579101323
1644	ILMN_2237474	0.0368357	TBC1D8B	Xq22.3b	-1.578991182
1715	ILMN_2366205	0.0391057	CACNB1	17q12c	-1.574539839
149	ILMN_1753567	0.0020553	ZNF451	6p12.1a	-1.572884474
1972	ILMN_2166582	0.0485356	CAMK4	5q22.1b	-1.572301997
1147	ILMN_2305599	0.0217509	LDB3	10q23.2a	-1.569753875
1623	ILMN_1658805	0.0362422	BRE	2p23.2b	-1.569300373
1000	ILMN_1790230	0.0180802	ZNF181	19q13.11c	-1.568361777

25	ILMN_1692271	0.0004431	CCM2	7p13c	-1.563241268
1453	ILMN_2265082	0.0301822	FUSIP1	1p36.11d	-1.562064858
4	ILMN_2341711	0.0000862	ISCA1L	5q12.1d	-1.559656876
1265	ILMN_1753663	0.0246874	ARL4A	7p21.3a	-1.559389506
1525	ILMN_1785010	0.0326705	TPTE2	13q12.11a	-1.553372491
662	ILMN_1806366	0.0108019	ENOX2	Xq25h-q26.1a	-1.550674744
535	ILMN_1697168	0.0087047	PRPF4B	6p25.2a	-1.55051529
1021	ILMN_1708959	0.0189585	MFN1	3q26.32c	-1.548304332
670	ILMN_2253272	0.010993	C1orf9	1q24.3d	-1.548027528
16	ILMN_1723398	0.000354	CSNK1G1	15q22.31a	-1.547782686
1003	ILMN_2205896	0.0181499	MEIS3P1		-1.545899885
1905	ILMN_1724915	0.0458729	C6orf191	6q22.33e	-1.544661349
90	ILMN_1699798	0.001259	HRAS	11p15.5d	-1.54465518
1184	ILMN_1784099	0.0227746	VPS13A	9q21.13c	-1.54437913
37	ILMN_2328952	0.0005605	CLCC1	1p13.3c-p13.3b	-1.542775885
70	ILMN_1717541	0.0010297	CNTNAP3	9p13.1Bp13.1a	-1.540142854
1284	ILMN_2397294	0.0252059	PLCB4	20p12.2b	-1.539821305
985	ILMN_1738572	0.0175794	USP48	1p36.12b	-1.539648149
1837	ILMN_1770857	0.0434953	SYT2	1q32.1d	-1.538811603
815	ILMN_2061641	0.0138677	SGOL2	2q33.1e	-1.538810974
1010	ILMN_1664560	0.0183222	DYRK1A	21q22.13b	-1.538056832
1293	ILMN_1803490	0.0256417	CCDC34	11p14.1d	-1.53722867
1368	ILMN_1813341	0.0274475	PTGFR	1p31.1e	-1.536839634
1241	ILMN_1787988	0.024201	UBE2D1	10q21.1e	-1.536341237
1920	ILMN_2193761	0.0464745	LOC442245		-1.53565212
929	ILMN_2225151	0.0160995	DLX6	7q21.3c	-1.535440803
68	ILMN_1657818	0.0009699	IQCB1	3q13.33c	-1.535400975
975	ILMN_2386040	0.0173869	MYO19	17q12b	-1.533441485
837	ILMN_1741143	0.0142681	TXK	4p12a	-1.530777572
157	ILMN_1731729	0.0021466	OCLN	5q13.2a	-1.530025071
461	ILMN_1803217	0.0073125	KIAA0514	10q11.22a	-1.529675424
781	ILMN_1747577	0.0133895	ALAD	9q32c	-1.529076318
103	ILMN_1798952	0.001412	KDELR3	22q13.1b	-1.528886658
31	ILMN_1769245	0.0004912	GLIPR1	12q21.2a	-1.528265649
1423	ILMN_1783840	0.0294419	FLJ42986	2q11.2e	-1.527526322
**#**	**Unique id**	**P-value**	**Symbol**	**Cytoband**	**Fold Change**
760	ILMN_1753166	0.0129621	PREI3	2q33.1b	3.163095105
253	ILMN_1715169	0.0034973	HLA-DRB1	6p21.32b	3.072859818
403	ILMN_1788874	0.0060707	SERPINA3	14q32.13b	2.941806537
487	ILMN_2206126	0.0077479	RAET1L	6q25.1b	2.633618398
972	ILMN_2074477	0.0173429	GPR4	19q13.32a	2.553466997
36	ILMN_1806607	0.0005519	SFN	1p36.11a	2.437586931
260	ILMN_2215418	0.0036355	SERPINA6	14q32.13a	2.435505988
175	ILMN_1689734	0.0023758	IL1RN	2q13d	2.362526116
53	ILMN_1737096	0.000787	CLDN10	13q32.1b	2.361541173
126	ILMN_1789162	0.0017554	MAB21L2	4q31.3a	2.324730682
209	ILMN_1755674	0.0028003	FAM12A	14q11.2b	2.311026679
10	ILMN_2309615	0.0001947	PSG6	19q13.31a	2.219710147
49	ILMN_1771841	0.0007337	FOSL1	11q13.1d	2.210575573
225	ILMN_2086143	0.0031126	CCR4	3p22.3c	2.208332245
339	ILMN_2313672	0.0048925	IL1RL1	2q12.1a	2.188175227
15	ILMN_1667754	0.0003509	MC3R	20q13.2d	2.186412895
1116	ILMN_1791759	0.0210929	CXCL10	4q21.1a	2.171008349
72	ILMN_1768120	0.0010429	C3orf48	3p24.3c	2.162513314
1024	ILMN_1693976	0.0189992	KRTAP9-3	17q21.2a	2.160325955
1289	ILMN_1781745	0.0253758	C9orf152	9q31.3a	2.153004856
131	ILMN_1722850	0.0018112	VGLL2	6q22.2a	2.146396291
214	ILMN_2071028	0.0028571	C3orf24	3p25.3c-p25.3b	2.139502961
767	ILMN_2275760	0.0131291	TFAP2A	6p24.3a	2.133200071
1175	ILMN_2070375	0.0226226	GPR82	Xp11.4a	2.12887513
1376	ILMN_1697069	0.0278279	CSF3	17q21.1a	2.119800211
459	ILMN_2194619	0.0072459	AMAC1L2	8p23.1b	2.116896693
881	ILMN_1716512	0.0152096	MGC70870		2.106414899
35	ILMN_1758164	0.0005274	STC1	8p21.2d	2.10637289
904	ILMN_1735737	0.0156016	SLC11A1	2q35e	2.101896093
404	ILMN_1653073	0.0060769	OR10Q1	11q12.1b	2.095635241
496	ILMN_1766401	0.0079355	OR1M1	19p13.2d	2.095066016
887	ILMN_2279645	0.0152821	BCL2L14	12p13.2a	2.083421271
99	ILMN_1779890	0.0013657	OTOA	16p12.2a	2.077906662

359	ILMN_1784884	0.0052713	LILRB3	19q13.42a	2.076099817
182	ILMN_1772179	0.0024228	OR2G2	1q44e	2.073002692
124	ILMN_1813776	0.001746	OR4D6	11q12.1d	2.067387091
6	ILMN_1693487	0.0001593	OR2W1	6p22.1a	2.066688293
107	ILMN_1695658	0.0014547	KIF20A	5q31.2c	2.062731605
366	ILMN_1731529	0.0053882	MS4A12	11q12.2a	2.050919002
593	ILMN_1787509	0.0095384	PRIC285	20q13.33e	2.048410321
424	ILMN_2361810	0.0064279	DRD3	3q13.31a	2.044891521
73	ILMN_1751028	0.0010487	SERPINH1	11q13.5a	2.044843707
116	ILMN_1774219	0.0015884	C2orf51	2p11.2c	2.035096123
248	ILMN_1764402	0.0033944	FAM12B	14q11.2b	2.033908337
647	ILMN_1669119	0.0104821	LOC728946		2.025835019
938	ILMN_1691264	0.0163231	NAT8B	2p13.2a	2.025108439
338	ILMN_1721916	0.0048807	LOC389151	3q22.3c	2.023243277
69	ILMN_2145490	0.0010248	LOC283677	15q24.1a	2.022440581
347	ILMN_1777917	0.00501	DSCR10	21q22.13b	2.016119774
1545	ILMN_1681576	0.0334144	IQCF2	3p21.1e	2.005690025
517	ILMN_1741165	0.008413	SLC11A1	2q35e	1.996124475
291	ILMN_1813206	0.0041172	CP	3q24f-q25.1a	1.99041984
471	ILMN_1815480	0.0074524	NAT8	2p13.2a	1.989906773
159	ILMN_1714841	0.0021933	OR4D9	11q12.1d	1.982635097
58	ILMN_1694166	0.0008844	HIST1H2A	6p22.2a	1.977219677
267	ILMN_1655565	0.0037057	UGT3A2	5p13.2c	1.975246829
860	ILMN_2092118	0.0147335	FPR1	19q13.33e	1.975030425
17	ILMN_1714446	0.0003614	PLG	6q26a	1.973673776
410	ILMN_1780368	0.0062054	GPR18	13q32.3a	1.968653067
208	ILMN_2123719	0.0027892	LOC651503	6p22.1a	1.967328655
247	ILMN_1774685	0.0033591	IL24	1q32.1h	1.964159629
165	ILMN_1710993	0.0022502	C1orf111	1q23.3b	1.952692909
3	ILMN_1682592	0.0000638	IL19	1q32.1h	1.949276851
215	ILMN_1713927	0.002866	AMTN	4q13.3a	1.948099745
543	ILMN_2415393	0.0088195	FSHB	11p14.1a	1.945617075
401	ILMN_1787212	0.0060387	CDKN1A	6p21.31a	1.940516202
421	ILMN_1808674	0.0064025	CPN1	10q24.2c	1.935519723
326	ILMN_1696284	0.0046968	CLDN18	3q22.3b	1.931688688
19	ILMN_1741430	0.000385	MAGEA10	Xq28e	1.927681042
12	ILMN_1694400	0.0002441	MSR1	8p22c	1.926152655
1397	ILMN_2322768	0.0285995	CSF3	17q21.1a	1.923600388
274	ILMN_1727459	0.0037901	ORC1L	1p32.3d	1.919166103
254	ILMN_1798000	0.0035331	PSG1	19q13.31a	1.918322512
143	ILMN_1749368	0.0019862	HIST1H3H	6p22.1c	1.916871375
500	ILMN_1783969	0.0080127	FAM24A	10q26.13b	1.916612987
51	ILMN_1775879	0.00076	KRTAP19-2	21q22.11a	1.913584764
672	ILMN_1672076	0.0110124	WFDC10B	20q13.12b	1.906941589
192	ILMN_1801584	0.0025158	CXCR4	2q21.3b	1.902028509
396	ILMN_1773848	0.0059503	C15orf32	15q26.1d	1.90004053
50	ILMN_2256050	0.0007436	SERPINA1	14q32.13a	1.899160712
173	ILMN_1697466	0.002367	OR5AY1	1q44e	1.898447824
529	ILMN_1686245	0.0085856	OR6Q1	11q12.1b	1.895309148
1275	ILMN_1711111	0.0249492	SLC9A10	3q13.2a	1.893507324
71	ILMN_1701376	0.0010374	MGC34821	11q12.3b	1.893141053
1288	ILMN_1813561	0.025332	SCIN	7p21.3a	1.8816685
342	ILMN_1705551	0.0049136	OR56A4	11p15.4c	1.876572144
170	ILMN_2168421	0.0023309	SPATA8	15q26.2c	1.875293706
453	ILMN_1749341	0.0069923	LOC554226		1.872041954
67	ILMN_1793182	0.0009661	SLC36A2	5q33.1d	1.869309791
1986	ILMN_2138801	0.0490656	TP73L	3q28b	1.866656901
280	ILMN_2357419	0.0039279	LILRA5	19q13.42a	1.865735075
1317	ILMN_2205012	0.0263422	SFTPA2	10q22.3f	1.864205816
93	ILMN_1738691	0.0013038	POU4F1	13q31.1a	1.863802677
648	ILMN_1811289	0.0104964	COL4A6	Xq22.3c	1.863073275
64	ILMN_1775257	0.0009608	PROK2	3p13d	1.862821045
384	ILMN_1731928	0.0057217	LY9	1q23.3a	1.861813297
320	ILMN_1708248	0.0046579	LILRB1	19q13.42a	1.85726951
1615	ILMN_1663793	0.0360043	MIST	4p16.1a	1.856856476
649	ILMN_1652137	0.0105149	PPYR1	10q11.22a	1.854606176
491	ILMN_1656802	0.0078408	LOXL2	8p21.3a	1.853159173
