# Supplementary material for: Age-Dependent Brain Gene Expression and Copy Number Anomalies in Autism Suggest Distinct Pathological Processes at Young Versus Mature Ages
Source: PLoS Genet. 2012 Mar 22;8(3):e1002592. doi: 10.1371/journal.pgen.1002592 (PMC3310790; doi:10.1371/journal.pgen.1002592)
Supplement: Table S6 — Significant dysregulated pathways of autistic cases across all young and older ages (2 to 56 years). Dysregulated pathways enriched in genes showing a main effect of diagnosis (p<0.05; FDR<0.1) identified using the MetaCore software suite between autism and control cases, p-value and network objects are listed. Pathways are sorted by pathway category name. (PDF) [file pgen.1002592.s010.pdf]

Supplementary Table 6: Dysregulated pathways of autistic cases across all young and older ages (2 to 56 years)			
**#**	**Name**	**pValue**	**Network**
24	Apoptosis and survival_BAD phosphorylation	3.18E-03	6/42
44	Cardiac Hypertrophy_NF-AT signaling in Cardiac Hypertrophy	7.36E-03	7/65
52	Cell adhesion_Cell-matrix glycoconjugates	9.90E-03	5/38
22	Cell adhesion_Chemokines and adhesion	2.54E-03	10/100
7	Cell adhesion_ECM remodeling	4.04E-04	8/52
30	Cell adhesion_Histamine H1 receptor signaling in the interruption of cell barrier integrity	4.52E-03	6/45
20	Cell adhesion_PLAU signaling	2.16E-03	6/39
8	Cell cycle_Role of 14-3-3 proteins in cell cycle regulation	8.20E-04	5/22
9	Cytoskeleton remodeling_Cytoskeleton remodeling	8.41E-04	11/102
50	Cytoskeleton remodeling_RalB regulation pathway	9.36E-03	3/13
17	Cytoskeleton remodeling_TGF, WNT and cytoskeletal remodeling	1.69E-03	11/111
1	Development_A2A receptor signaling	1.70E-07	11/43
2	Development_A2B receptor: action via G-protein alpha s	7.05E-06	10/50
15	Development_A3 receptor signaling	1.46E-03	7/49
46	Development_Activation of ERK by Kappa-type opioid receptor	7.87E-03	5/36
35	Development_Angiotensin activation of ERK	5.40E-03	5/33
33	Development_G-Proteins mediated regulation MARK-ERK signaling	5.04E-03	6/46
34	Development_GDNF family signaling	5.04E-03	6/46
16	Development_GM-CSF signaling	1.65E-03	7/50
48	Development_Hedgehog and PTH signaling pathways in bone and cartilage development	8.85E-03	5/37
14	Development_HGF signaling pathway	1.13E-03	7/47
32	Development_HGF-dependent inhibition of TGF-beta-induced EMT	4.71E-03	5/32
18	Development_IGF-1 receptor signaling	1.85E-03	7/51
25	Development_Osteopontin signaling in osteoclasts	3.53E-03	5/30
6	Development_PIP3 signaling in cardiac myocytes	1.97E-04	8/47
11	Development_Role of IL-8 in angiogenesis	8.58E-04	8/58
45	Development_TGF-beta receptor signaling	7.62E-03	6/50
37	Development_TGF-beta-dependent induction of EMT via MAPK	5.62E-03	6/47
41	Development_Thrombopoetin signaling via JAK-STAT pathway	6.54E-03	4/22
5	Development_Thrombopoietin-regulated cell processes	1.43E-04	8/45
21	DNA damage_Role of SUMO in p53 regulation	2.44E-03	4/17
38	G-protein signaling_G-Protein alpha-q signaling cascades	6.15E-03	5/34
28	Immune response_ETV3 affect on CSF1-promoted macrophage differentiation	4.09E-03	5/31
26	Immune response_IL-4 - antiapoptotic action	3.53E-03	5/30
12	Immune response_MIF - the neuroendocrine-macrophage connector	9.95E-04	7/46
47	Membrane-bound ESR1: interaction with G-proteins signaling	8.39E-03	6/51
39	Muscle contraction_Relaxin signaling pathway	6.24E-03	6/48
43	Neurophysiological process_Long-term depression in cerebellum	6.90E-03	6/49
23	Proteolysis_Putative SUMO-1 pathway	3.03E-03	5/29
29	Regulation of lipid metabolism_Regulation of lipid metabolism by niacin and isoprenaline	4.52E-03	6/45
51	Regulation of lipid metabolism_Regulation of lipid metabolism via LXR, NF-Y and SREBP	9.90E-03	5/38
10	Reproduction_GnRH signaling	8.58E-04	9/72
49	Signal transduction_Activation of PKC via G-Protein coupled receptor	9.21E-03	6/52
27	Signal transduction_AKT signaling	3.58E-03	6/43
19	Signal transduction_cAMP signaling	1.88E-03	6/38
42	Signal transduction_IP3 signaling	6.90E-03	6/49
13	Signal transduction_PTEN pathway	9.95E-04	7/46
31	Transcription_Androgen Receptor nuclear signaling	4.52E-03	6/45
36	Transcription_ChREBP regulation pathway	5.50E-03	4/21
4	Transcription_CREB pathway	1.21E-04	8/44
3	Transcription_P53 signaling pathway	4.90E-05	8/39
40	Transcription_Role of heterochromatin protein 1 (HP1) family in transcriptional silencing	6.54E-03	4/22
