# Supplementary material for: Age-Dependent Brain Gene Expression and Copy Number Anomalies in Autism Suggest Distinct Pathological Processes at Young Versus Mature Ages
Source: PLoS Genet. 2012 Mar 22;8(3):e1002592. doi: 10.1371/journal.pgen.1002592 (PMC3310790; doi:10.1371/journal.pgen.1002592)
Supplement: Table S7 — Chromosomal locations of CNV regions found in all autistic cases (males and females). Chromosomal location, number (N) of SNPs, size of the SNP in base pairs (bp) and annotated gene content of CNV regions found in all autistic cases (males and females) are listed. Overlapping regions with the same gene content but different breakpoints are listed as distinct CNVs. Enrichment results are presented in Figure 4. (PDF) [file pgen.1002592.s011.pdf]

Supplementary Table 7: Chromosomal locations of CNV regions found in all autistic cases (males and females)				
**Location**	**N**	**Size**	**Del/Dup**	**CNV Gene Content**
chr1:148542646-148699426	15	156781	Del	MRPS21,PRPF3,RPRD2
chr1:159566115-159596015	5	29901	Del	SDHC
chr1:159566115-159605684	6	39570	Del	C1orf192,SDHC
chr1:183388645-183438492	6	49848	Del	C1orf25,C1orf26
chr1:198226632-198264026	8	37395	Del	NR5A2
chr1:198736128-198748379	8	12252	Del	
chr1:209418136-209449752	8	31617	Del	
chr1:210362194-210391111	8	28918	Del	
chr1:211088985-211131668	11	42684	Del	FLVCR1,NCRNA00292
chr1:234525338-234556802	6	31465	Del	
chr1:234879762-234895971	5	16210	Del	
chr1:27911734-27979646	10	67913	Del	FAM76A,STX12
chr1:27944423-27979646	7	35224	Del	FAM76A,STX12
chr1:28522964-28847988	15	325025	Del	MED18,PHACTR4,RAB42,RCC1,RNU11,SNHG12,SNHG3,SNORA16A
chr1:28532283-28811725	13	279443	Del	MED18,PHACTR4,RAB42,RCC1,SNHG12,SNHG3,SNORA16A,SNOR
chr1:28608937-28811725	11	202789	Del	PHACTR4,RAB42,RCC1,SNHG12,SNHG3,SNORA16A,SNORA44,SNO
chr1:3043966-3099011	15	55046	Dup	PRDM16
chr1:32803048-32907361	7	104314	Del	RBBP4,ZBTB8A,ZBTB8OS
chr1:33071409-33094383	6	22975	Del	S100PBP
chr1:62430118-62464680	13	34563	Del	L1TD1
chr1:79256875-79266524	6	9650	Del	
chr1:93283975-93304294	9	20320	Del	
chr1:93287637-93304294	8	16658	Del	
chr2:120457553-120619637	12	162085	Del	EPB41L5
chr2:172523756-172565120	6	41365	Del	HAT1
chr2:172523756-172604833	10	81078	Del	HAT1,METAP1D
chr2:174951747-175039184	16	87438	Del	CIR1,GPR155,SCRN3
chr2:174987149-175047750	11	60602	Del	GPR155,SCRN3
chr2:202873779-203017311	14	143533	Del	BMPR2,NOP58
chr2:202873779-203140049	24	266271	Del	BMPR2,NOP58
chr2:203017311-203140049	11	122739	Del	BMPR2
chr2:203454130-203873416	25	419287	Del	ALS2CR8,CYP20A1,NBEAL1,WDR12
chr2:203454130-203934576	28	480447	Del	ABI2,ALS2CR8,CYP20A1,NBEAL1,WDR12
chr2:224324400-224421599	19	97200	Del	AP1S3
chr2:23939110-24044136	8	105027	Del	ATAD2B,UBXN2A
chr2:38876325-38895284	6	18960	Del	DHX57
chr2:38876325-38900658	7	24334	Del	DHX57
chr2:38876325-38963062	18	86738	Del	DHX57,MORN2
chr2:47452958-47467374	6	14417	Del	EPCAM,MIR559
chr2:54178205-54239579	13	61375	Del	ACYP2
chr2:54212021-54239579	10	27559	Del	ACYP2
chr2:55274329-55345580	14	71252	Del	C2orf63,MTIF2,RPS27A
chr2:61183721-61222036	8	38316	Del	KIAA1841
chr2:61326136-61394146	10	68011	Del	USP34
chr2:61723068-61772891	7	49824	Del	
chr2:78625722-78636272	5	10551	Del	
chr3:104579967-104598179	6	18213	Del	
chr3:110555013-110575074	7	20062	Del	
chr3:113735222-113815319	7	80098	Del	ATG3,CCDC80,SLC35A5
chr3:137570728-137783422	15	212695	Del	STAG1
chr3:139934042-140084943	11	150902	Del	PIK3CB
chr3:139993073-140084943	7	91871	Del	
chr3:143182477-143222766	7	40290	Del	TFDP2
chr3:149257402-149280584	5	23183	Del	
chr3:171401005-171415703	5	14699	Del	
chr3:171448794-171577602	16	128809	Del	PRKCI,SKIL
chr3:182960104-183075368	25	115265	Del	

chr3:182998542-183040424	8	41883	Del	
chr3:182998542-183044024	9	45483	Del	
chr3:183004714-183054110	8	49397	Del	
chr3:27506800-27529296	6	22497	Del	
chr3:32674426-32814611	15	140186	Del	CNOT10
chr3:41935010-42026840	8	91831	Del	ULK4
chr3:47682816-47746291	10	63476	Del	SMARCC1
chr3:47682816-47797547	13	114732	Del	SMARCC1
chr3:57432480-57464631	10	32152	Del	DNAH12
chr3:57432480-57469178	11	36699	Del	DNAH12
chr4:113714056-113919598	18	205543	Del	C4orf21,LARP7,MIR302A,MIR302B,MIR302C,MIR302D,MIR367
chr4:113744172-113832321	8	88150	Del	C4orf21,LARP7,MIR302A,MIR302B,MIR302C,MIR302D,MIR367
chr4:113744172-113919598	17	175427	Del	C4orf21,LARP7,MIR302A,MIR302B,MIR302C,MIR302D,MIR367
chr4:113759418-113832321	6	72904	Del	C4orf21,LARP7,MIR302A,MIR302B,MIR302C,MIR302D,MIR367
chr4:154533886-154591421	8	57536	Del	MND1
chr4:154533886-154610890	10	77005	Del	KIAA0922,MND1
chr4:166189075-166211082	7	22008	Del	
chr4:166189075-166226719	9	37645	Del	TMEM192
chr4:166259035-166277171	5	18137	Del	
chr4:24872576-24903317	5	30742	Del	PI4K2B
chr4:39068335-39097264	7	28930	Del	KLB
chr4:39217807-39266759	6	48953	Del	C4orf34
chr4:39217807-39282064	8	64258	Del	C4orf34
chr4:39224507-39276405	6	51899	Del	C4orf34
chr4:39224507-39282064	7	57558	Del	C4orf34
chr4:39675489-39797957	15	122469	Del	LOC344967,N4BP2
chr4:39675489-39810009	17	134521	Del	LOC344967,N4BP2
chr4:41626538-41720041	11	93504	Del	DCAF4L1,SLC30A9,TMEM33
chr4:56188697-56227094	7	38398	Del	NMU
chr4:71943570-71995799	7	52230	Del	MOBKL1A
chr4:71967109-72178204	18	211096	Del	DCK,MOBKL1A
chr4:77065779-77108118	7	42340	Del	NAAA,SDAD1
chr4:89525529-89535789	7	10261	Del	HERC6
chr4:92502794-92541356	6	38563	Del	FAM190A
chr5:125933468-126000523	12	67056	Del	ALDH7A1,C5orf48,PHAX
chr5:125933468-126082966	21	149499	Del	ALDH7A1,C5orf48,PHAX
chr5:129902971-129949287	5	46317	Del	
chr5:130644691-130701975	6	57285	Del	CDC42SE2
chr5:145568676-145667488	15	98813	Del	RBM27
chr5:156432559-156464314	6	31756	Del	HAVCR2
chr5:37349755-37427371	8	77617	Del	NUP155,WDR70
chr5:37354052-37473605	13	119554	Del	NUP155,WDR70
chr5:61775220-61930880	14	155661	Del	IPO11,LRRC70
chr5:68415592-68470725	10	55134	Del	SLC30A5
chr5:68432336-68504814	13	72479	Del	CCNB1,SLC30A5
chr5:78556487-78593704	5	37218	Del	JMY
chr5:79600414-79614553	6	14140	Del	
chr5:79600414-79616574	7	16161	Del	
chr5:79841521-79883132	5	41612	Del	FAM151B
chr6:111057837-111264286	18	206450	Del	CDK19
chr6:111384438-111473158	17	88721	Del	GTF3C6,RPF2
chr6:134550947-134600782	10	49836	Del	SGK1
chr6:134550947-134604849	14	53903	Del	SGK1
chr6:134557712-134596031	6	38320	Del	SGK1
chr6:13808398-13863495	9	55098	Del	RANBP9
chr6:139202954-139228150	5	25197	Del	ECT2L
chr6:149951184-150156438	17	205255	Del	C6orf72,KATNA1,LATS1,NUP43,PCMT1
chr6:149999877-150156438	14	156562	Del	KATNA1,LATS1,NUP43,PCMT1

chr6:149999877-150208262	19	208386	Del	KATNA1,LATS1,LRP11,NUP43,PCMT1
chr6:17470094-17489796	5	19703	Del	
chr6:17470094-17494874	6	24781	Del	
chr6:20612830-20632361	7	19532	Del	
chr6:24477494-24509633	6	32140	Del	DCDC2
chr6:42598500-42735412	12	136913	Del	UBR2
chr6:71384308-71408533	7	24226	Del	
chr6:74163467-74434186	37	270720	Del	C6orf150,DDX43,EEF1A1,MTO1,SLC17A5
chr6:74266184-74434186	26	168003	Del	EEF1A1,MTO1,SLC17A5
chr6:74266184-74447143	31	180960	Del	EEF1A1,MTO1,SLC17A5
chr6:74392545-74434186	10	41642	Del	SLC17A5
chr6:86262042-86321093	8	59052	Del	NT5E,SNX14
chr6:86262042-86336456	10	74415	Del	NT5E,SNX14
chr6:89816359-89925006	14	108648	Del	PM20D2,PNRC1,SRSF12
chr7:113873430-113934453	8	61024	Del	FOXP2
chr7:134785342-134820988	6	35647	Del	CNOT4
chr7:138008996-138028345	8	19350	Del	SVOPL
chr7:138008996-138031017	10	22022	Del	SVOPL
chr7:138635584-138709343	7	73760	Del	C7orf55,LUC7L2,UBN2
chr7:151809888-151873168	9	63281	Del	
chr7:151809888-151886472	10	76585	Del	
chr7:63065485-63088984	8	23500	Del	
chr7:73272823-73321316	6	48494	Del	LAT2,RFC2
chr7:77060822-77354679	31	293858	Del	PHTF2,PTPN12,RSBN1L,TMEM60
chr7:77106138-77158324	8	52187	Del	PTPN12
chr7:94651324-94678852	5	27529	Del	PPP1R9A
chr8:70990827-71020425	11	29599	Del	
chr8:70990827-71033622	15	42796	Del	
chr8:96840081-96907849	18	67769	Del	
chr9:131706896-131822805	13	115910	Del	FNBP1
chr9:131706896-131827349	15	120454	Del	FNBP1
chr9:33998406-34072144	13	73739	Del	UBAP2
chr9:33998406-34085717	17	87312	Del	DCAF12,UBAP2
chr9:36515772-36637786	11	122015	Del	MELK
chr9:5956898-5968437	5	11540	Del	KIAA2026
chr9:73884517-73962968	15	78452	Del	GDA
chr10:12068234-12175894	19	107661	Del	DHTKD1,UPF2
chr10:12079184-12186864	19	107681	Del	DHTKD1,UPF2
chr10:12332460-12399447	17	66988	Del	CDC123
chr10:123686677-	17	85727	Del	NSMCE4A,TACC2
chr10:12369408-12399447	8	30040	Del	
chr10:12383635-12391095	5	7461	Del	
chr10:15097380-15129986	12	32607	Del	OLAH
chr10:15483641-15510072	6	26432	Del	
chr10:26863051-26898866	11	35816	Del	APBB1IP
chr10:69797702-69933585	13	135884	Del	DNA2,RUFY2,SLC25A16
chr10:70002586-70051899	8	49314	Del	TET1
chr10:73648734-73666540	5	17807	Del	ANAPC16
chr10:74025594-74133869	6	108276	Del	CBARA1,CCDC109A
chr11:10057767-10099689	5	41923	Del	SBF2
chr11:118210355-	8	30332	Del	
chr11:32483814-32552861	15	69048	Del	
chr11:80606592-80620137	14	13546	Del	
chr11:9265981-9320188	8	54208	Del	TMEM41B
chr11:9346451-9496239	17	149789	Del	IPO7,LOC644656,SNORA23,ZNF143
chr11:97676619-97795166	21	118548	Del	
chr12:100801180-	7	17570	Del	DRAM1
chr12:10492957-10600080	13	107124	Del	KLRC1
chr12:121368594-	30	320618	Del	CLIP1,KNTC1,RSRC2,ZCCHC8

121689211				
chr12:32077191-32121084	18	43894	Del	
chr12:32107892-32121084	6	13193	Del	
chr12:32107892-32126302	8	18411	Del	
chr12:49014078-49127324	14	113247	Del	FAM186A,LARP4
chr12:49014078-49145087	17	131010	Del	FAM186A,LARP4
chr12:62930978-63035639	19	104662	Del	C12orf56
chr12:917792-948337	12	30546	Del	RAD52
chr12:942651-1056227	24	113577	Del	ERC1
chr13:41693991-41699654	5	5664	Del	DGKH
chr13:49192057-49239176	9	47120	Del	KPNA3
chr13:79722991-79809526	13	86536	Del	SPRY2
chr13:80558774-80571474	5	12701	Del	
chr13:94967687-94984576	5	16890	Del	CLDN10
chr14:35148467-35216763	7	68297	Del	RALGAPA1
chr14:35148467-35371747	19	223281	Del	BRMS1L,RALGAPA1
chr14:35148467-35408995	20	260529	Del	BRMS1L,RALGAPA1
chr14:46720671-46767552	11	46882	Del	MDGA2
chr14:52200902-52228076	8	27175	Del	ERO1L
chr14:54373759-54509818	17	136060	Del	GCH1,MIR4308,WDHD1
chr14:58050270-58091995	5	41726	Del	KIAA0586
chr14:63089400-63284561	13	195162	Del	SGPP1,WDR89
chr14:73171175-73238184	8	67010	Del	DNAL1
chr14:73874924-73887171	6	12248	Del	C14orf115
chr14:77057711-77110927	20	53217	Del	SPTLC2
chr14:77098556-77110927	5	12372	Del	SPTLC2
chr14:91569101-91670551	20	101451	Del	ATXN3,CPSF2,NDUFB1,TRIP11
chr14:91593892-91625808	7	31917	Del	ATXN3
chr15:33004392-33041888	6	37497	Del	AQR
chr15:38265123-38315447	6	50325	Del	BUB1B,PAK6
chr15:39421879-39439013	5	17135	Del	NUSAP1
chr15:39421879-39476524	7	54646	Del	NDUFAF1,NUSAP1
chr15:40988401-41123636	8	135236	Del	TTBK2,UBR1
chr15:41081510-41123636	5	42127	Del	UBR1
chr15:48476200-48500329	5	24130	Del	
chr15:48489350-48577525	11	88176	Del	USP8
chr15:53414820-53547867	19	133048	Del	CCPG1,DYX1C1,MIR628,PIGB
chr15:53475261-53547867	11	72607	Del	CCPG1,DYX1C1
chr15:53480496-53547867	10	67372	Del	CCPG1,DYX1C1
chr15:53480496-53565080	12	84585	Del	CCPG1,DYX1C1
chr15:53482122-53547867	9	65746	Del	CCPG1,DYX1C1
chr15:62381664-62572511	12	190848	Del	CSNK1G1,KIAA0101,TRIP4
chr15:63372790-63417235	10	44446	Del	IGDCC3
chr15:73542520-73711498	10	168979	Del	PTPN9,SNUPN
chr15:73557085-73639839	5	82755	Del	PTPN9
chr15:74758511-74963213	16	204703	Del	SCAPER
chr15:74938615-74963213	7	24599	Del	SCAPER
chr15:83265502-83301233	20	35732	Del	SLC28A1
chr15:88174443-88201888	6	27446	Del	AP3S2
chr15:96929526-96950172	11	20647	Del	
chr16:73220098-73241238	5	21141	Del	RFWD3
chr17:16020935-16218501	18	197567	Del	CENPV,MIR1288,NCOR1,PIGL
chr17:16020935-16259657	21	238723	Del	CENPV,MIR1288,NCOR1,PIGL,TRPV2,UBB
chr17:16125354-16266693	17	141340	Del	CENPV,MIR1288,PIGL,TRPV2,UBB
chr17:2456111-2507919	6	51809	Del	PAFAH1B1
chr17:24587116-24627212	8	40097	Del	CRYBA1,NUFIP2
chr17:25481175-25555919	9	74745	Del	CCDC55,SLC6A4
chr17:26097956-26250354	9	152399	Del	ATAD5,C17orf42,CRLF3,SUZ12P

chr17:26131834-26250354	8	118521	Del	ATAD5,C17orf42,CRLF3
chr17:26138589-26250354	7	111766	Del	ATAD5,C17orf42,CRLF3
chr17:27214461-27246115	6	31655	Del	UTP6
chr17:34761298-34863649	5	102352	Del	FBXL20,MED1
chr17:34761298-34869863	6	108566	Del	FBXL20,MED1
chr17:38465179-38552381	13	87203	Del	BRCA1,NBR2
chr17:54063018-54698963	35	635946	Del	C17orf71,GDPD1,MIR301A,MIR454,PPM1E,PRR11,RAD51C,SKA2,TE
chr17:55173488-55351797	15	178310	Del	MIR21,RPS6KB1,TMEM49,TUBD1
chr17:63187880-63236615	6	48736	Del	
chr17:70602916-70715732	9	112817	Del	ARMC7,HN1,NT5C,NUP85,SLC16A5,SUMO2
chr18:16954953-17049834	10	94882	Del	
chr18:17202748-17526315	18	323568	Del	ABHD3,ESCO1,GREB1L,MIR320C1,SNRPD1
chr18:2584549-2632376	14	47828	Del	NDC80
chr18:27852807-27910954	10	58148	Del	RNF125
chr18:3559620-3565933	6	6314	Del	DLGAP1
chr18:3559620-3570742	10	11123	Del	DLGAP1
chr18:50007104-50029385	5	22282	Del	
chr18:54622597-54679876	6	57280	Del	
chr18:59167645-59180491	5	12847	Del	KDSR
chr19:19759149-19814114	5	54966	Del	ZNF506
chr19:62298373-62341712	14	43340	Del	USP29,ZIM3
chr20:4985774-5006969	8	21196	Del	
chr20:49892937-49941210	18	48274	Del	
chr20:49981704-50065689	27	83986	Del	
chr20:50031593-50065689	10	34097	Del	
chr21:26056693-26094576	5	37884	Del	GABPA
