# Supplementary material for: Age-Dependent Brain Gene Expression and Copy Number Anomalies in Autism Suggest Distinct Pathological Processes at Young Versus Mature Ages
Source: PLoS Genet. 2012 Mar 22;8(3):e1002592. doi: 10.1371/journal.pgen.1002592 (PMC3310790; doi:10.1371/journal.pgen.1002592)
Supplement: Table S8 — Chromosomal locations of CNV regions found in all control cases (males and females). Chromosomal location, number (N) of SNPs, size of the SNP in base pairs (bp) and annotated gene content of CNV regions found in all control cases (males and females) are listed. Overlapping regions with the same gene content but different breakpoints are listed as distinct CNVs. Enrichment results are presented in Figure 4. (PDF) [file pgen.1002592.s012.pdf]

Supplementary Table 8: Chromosomal locations of CNV regions found in all control cases (males and females)				
**Location**	**N**	**Size**	**Del/Dup**	**CNV Gene content**
chr1:159566115-159596015	5	29901	Del	SDHC
chr1:159566115-159605684	6	39570	Del	C1orf192,SDHC
chr1:172041575-172098724	5	57150	Del	CENPL,DARS2
chr1:174450679-174491369	8	40691	Del	
chr1:177194050-177223176	9	29127	Del	
chr1:177694193-177740676	10	46484	Del	C1orf125
chr1:209418136-209444575	7	26440	Del	
chr1:209418136-209449752	8	31617	Del	
chr1:211088985-211131668	11	42684	Del	FLVCR1,NCRNA00292
chr1:211097781-211131668	10	33888	Del	FLVCR1,NCRNA00292
chr1:234879762-234905949	8	26188	Del	
chr1:234883633-234905949	7	22317	Del	
chr1:244921485-244938321	5	16837	Del	
chr1:24859464-25002670	11	143207	Del	CLIC4,SRRM1
chr1:27944423-27979646	7	35224	Del	FAM76A,STX12
chr1:28512480-28614909	11	102430	Del	MED18,PHACTR4
chr1:28512480-28774042	15	261563	Del	MED18,PHACTR4,RCC1,SNHG3,TRNAU1AP
chr1:28512480-28811725	16	299246	Del	MED18,PHACTR4,RAB42,RCC1,SNHG12,SNHG3,SNORA16A,SN
chr1:28522964-28811725	14	288762	Del	MED18,PHACTR4,RAB42,RCC1,SNHG12,SNHG3,SNORA16A,SN
chr1:28532283-28614909	8	82627	Del	MED18,PHACTR4
chr1:28608937-28614909	6	5973	Del	PHACTR4
chr1:32778890-32839178	6	60289	Del	ZBTB8A
chr1:32785388-32839178	5	53791	Del	ZBTB8A
chr1:36262773-36321744	6	58972	Del	EIF2C3
chr1:3980640-4695274	259	714635	Dup	AJAP1,LOC284661
chr1:61936303-61972291	6	35989	Del	TM2D1
chr1:65352916-65365995	7	13080	Del	
chr1:77575717-77583700	5	7984	Del	AK5
chr1:92983464-93047038	5	63575	Del	EVI5
chr1:9909594-9958596	7	49003	Del	LZIC,NMNAT1
chr1:9909594-9969047	8	59454	Del	LZIC,NMNAT1
chr2:122181035-122284084	11	103050	Del	MKI67IP,TSN
chr2:127027131-127047424	5	20294	Del	
chr2:159860033-159872962	7	12930	Del	
chr2:159860033-159890548	9	30516	Del	BAZ2B
chr2:159860033-159901397	13	41365	Del	BAZ2B
chr2:172523756-172565120	6	41365	Del	HAT1
chr2:174987149-175039184	10	52036	Del	GPR155,SCRN3
chr2:174994117-175024625	5	30509	Del	GPR155,SCRN3
chr2:201421699-201444411	5	22713	Del	CLK1,PPIL3
chr2:201632323-201677185	5	44863	Del	FAM126B,NDUFB3
chr2:202834698-202934594	12	99897	Del	NOP58,SNORD11,SNORD11B,SNORD70
chr2:202834698-203140049	27	305352	Del	BMPR2,NOP58,SNORD11,SNORD11B,SNORD70
chr2:203543422-203873416	18	329995	Del	ALS2CR8,CYP20A1,NBEAL1
chr2:224324400-224421599	19	97200	Del	AP1S3
chr2:224366977-224421599	9	54623	Del	AP1S3
chr2:228315600-228330372	7	14773	Del	
chr2:23939110-24044136	8	105027	Del	ATAD2B,UBXN2A
chr2:32132286-32326630	10	194345	Del	NLRC4,SLC30A6,SPAST
chr2:38862453-38879136	5	16684	Del	DHX57,GEMIN6
chr2:38895284-38959285	12	64002	Del	DHX57,MORN2
chr2:39226711-39259074	8	32364	Del	
chr2:42547841-42800389	26	252549	Del	KCNG3,MTA3
chr2:42713218-42790051	9	76834	Del	MTA3
chr2:47437468-47467374	8	29907	Del	EPCAM,MIR559
chr2:47452958-47467374	6	14417	Del	EPCAM,MIR559
chr2:47454610-47500472	8	45863	Del	EPCAM,MIR559,MSH2

chr2:65248218-65329904	12	81687	Del	ACTR2
chr2:65251099-65329904	10	78806	Del	ACTR2
chr2:65251099-65345306	15	94208	Del	ACTR2
chr3:102789197-102828655	6	39459	Del	PCNP
chr3:104579967-104598179	6	18213	Del	
chr3:110495798-110598826	23	103029	Del	DPPA2,DPPA4
chr3:114896721-115083660	15	186940	Del	ATP6V1A,GRAMD1C,KIAA2018,NAA50
chr3:134057434-134079689	5	22256	Del	NCRNA00119
chr3:137570728-137796809	16	226082	Del	STAG1
chr3:139868105-140084943	17	216839	Del	PIK3CB
chr3:139934042-140084943	11	150902	Del	PIK3CB
chr3:143702232-143775208	9	72977	Del	ATR
chr3:149434837-149443355	5	8519	Del	
chr3:171401005-171415703	5	14699	Del	
chr3:171401005-171525496	18	124492	Del	PRKCI
chr3:171525496-171577602	8	52107	Del	SKIL
chr3:182998542-183044024	9	45483	Del	
chr3:182998542-183075368	15	76827	Del	
chr3:183007211-183056639	8	49429	Del	
chr3:183010723-183075368	11	64646	Del	
chr3:187089013-187100960	6	11948	Del	
chr3:27465198-27545290	8	80093	Del	SLC4A7
chr3:41802030-41834977	5	32948	Del	ULK4
chr3:41935010-41982343	5	47334	Del	ULK4
chr3:57432480-57457928	8	25449	Del	DNAH12
chr3:57432480-57739237	39	306758	Del	ARF4,DNAH12,FAM116A,PDE12,SLMAP
chr4:111301759-111392827	19	91069	Dup	ELOVL6
chr4:113744172-113832321	8	88150	Del	C4orf21,LARP7,MIR302A,MIR302B,MIR302C,MIR302D,MIR367
chr4:113798058-113883554	8	85497	Del	LARP7
chr4:129180019-129243723	5	63705	Del	LARP1B
chr4:129901826-129923483	7	21658	Del	
chr4:140371186-140407790	9	36605	Del	C4orf49
chr4:150604556-150652973	5	48418	Del	
chr4:154533886-154591421	8	57536	Del	MND1
chr4:154569464-154591421	5	21958	Del	
chr4:163678637-163785175	17	106539	Del	
chr4:183476755-183512752	15	35998	Del	ODZ3
chr4:24872576-24903317	5	30742	Del	PI4K2B
chr4:39068335-39114544	13	46210	Del	KLB
chr4:39198618-39234259	12	35642	Del	C4orf34,UGDH
chr4:39214189-39266759	9	52571	Del	C4orf34
chr4:39217807-39276405	7	58599	Del	C4orf34
chr4:39217807-39414067	28	196261	Del	C4orf34,UBE2K
chr4:39331852-39375133	8	43282	Del	
chr4:39331852-39494511	25	162660	Del	UBE2K
chr4:39362912-39387042	8	24131	Del	UBE2K
chr4:39362912-39494511	22	131600	Del	UBE2K
chr4:39482900-39494511	6	11612	Del	
chr4:39482900-39520488	10	37589	Del	PDS5A
chr4:39712008-39810009	14	98002	Del	LOC344967,N4BP2
chr4:42211968-42256079	11	44112	Dup	ATP8A1,MIR548M
chr4:54705880-54751401	10	45522	Del	
chr4:62634834-62742267	11	107434	Del	
chr4:71930576-71995799	8	65224	Del	MOBKL1A
chr4:71967109-72211493	22	244385	Del	DCK,MOBKL1A
chr4:72167168-72208285	8	41118	Del	
chr4:72176427-72218576	10	42150	Del	
chr5:100451980-100522078	5	70099	Del	
chr5:125911240-126000523	23	89284	Del	ALDH7A1,C5orf48,PHAX

chr5:125929340-126057234	20	127895	Del	ALDH7A1,C5orf48,PHAX
chr5:130609094-130688294	9	79201	Del	CDC42SE2
chr5:130644691-130688294	5	43604	Del	CDC42SE2
chr5:130644691-130701975	6	57285	Del	CDC42SE2
chr5:134001788-134218124	14	216337	Del	C5orf24,CAMLG,DDX46,SEC24A
chr5:137846077-137885966	5	39890	Del	ETF1
chr5:145542758-145667488	19	124731	Del	RBM27
chr5:145563816-145667488	16	103673	Del	RBM27
chr5:145568676-145667488	15	98813	Del	RBM27
chr5:156351368-156407976	9	56609	Del	HAVCR1
chr5:31902694-31921478	10	18785	Del	PDZD2
chr5:37321333-37427371	11	106039	Del	NUP155,WDR70
chr5:37321333-37473605	17	152273	Del	NUP155,WDR70
chr5:37321333-37514955	18	193623	Del	NUP155,WDR70
chr5:37354052-37473605	13	119554	Del	NUP155,WDR70
chr5:55118231-55144489	6	26259	Del	DDX4
chr5:65207263-65256098	11	48836	Del	
chr5:68443472-68470725	8	27254	Del	SLC30A5
chr5:72283624-72331333	6	47710	Del	FCHO2
chr5:76085588-76129130	10	43543	Del	
chr5:76097012-76129130	7	32119	Del	
chr5:79578880-79616574	10	37695	Del	SERINC5
chr5:79600414-79616574	7	16161	Del	
chr5:99895770-99934121	9	38352	Del	FAM174A
chr6:111384438-111450087	12	65650	Del	GTF3C6,RPF2
chr6:111399325-111443128	10	43804	Del	RPF2
chr6:134557712-134596031	6	38320	Del	SGK1
chr6:13827652-13888496	13	60845	Del	
chr6:139586039-139659446	13	73408	Del	TXLNB
chr6:15261077-15309758	8	48682	Del	
chr6:155865681-155884768	7	19088	Del	
chr6:17458478-17480021	6	21544	Del	
chr6:17470094-17519564	8	49471	Del	CAP2
chr6:18265947-18294526	7	28580	Del	KDM1B
chr6:20612830-20632361	7	19532	Del	
chr6:21720928-21801131	9	80204	Del	FLJ22536
chr6:42879360-42931507	5	52148	Del	KIAA0240
chr6:73979282-74013350	7	34069	Del	KHDC1,KHDC1L
chr6:74163467-74398310	30	234844	Del	C6orf150,DDX43,EEF1A1,MTO1,SLC17A5
chr6:74212067-74404863	26	192797	Del	C6orf150,EEF1A1,MTO1,SLC17A5
chr6:74212067-74410896	28	198830	Del	C6orf150,EEF1A1,MTO1,SLC17A5
chr6:74266184-74309987	8	43804	Del	EEF1A1,MTO1
chr6:74303113-74410896	16	107784	Del	SLC17A5
chr6:74309987-74347012	7	37026	Del	
chr6:76462420-76519431	7	57012	Del	MYO6,SENP6
chr6:86262042-86311241	7	49200	Del	NT5E,SNX14
chr6:86262042-86336456	10	74415	Del	NT5E,SNX14
chr6:88375602-88424354	10	48753	Del	ORC3
chr6:89826343-89925006	13	98664	Del	PM20D2,PNRC1,SRSF12
chr7:129374460-129424930	9	50471	Del	UBE2H
chr7:129394423-129416799	5	22377	Del	
chr7:129481974-129525687	7	43714	Del	KLHDC10
chr7:137996551-138028345	9	31795	Del	SVOPL
chr7:138137036-138159945	8	22910	Del	TMEM213
chr7:142741558-142756304	6	14747	Del	CLCN1
chr7:151809888-151873168	9	63281	Del	
chr7:151809888-151886472	10	76585	Del	
chr7:151916674-152026508	23	109835	Del	XRCC2
chr7:152011384-152026508	6	15125	Del	

chr7:23495452-23531150	5	35699	Del	RPS2P32,TRA2A
chr7:39923354-39997013	8	73660	Del	CDK13
chr7:68462034-68576554	16	114521	Del	
chr7:77106138-77226404	15	120267	Del	PTPN12,RSBN1L
chr7:97485127-97522404	9	37278	Del	
chr8:121011481-121063575	13	52095	Del	DEPDC6
chr8:30322253-30357424	9	35172	Del	
chr8:30574742-30655202	11	80461	Del	GSR,GTF2E2
chr8:70990827-71020425	11	29599	Del	
chr8:70990827-71053049	18	62223	Del	
chr8:70990827-71074850	20	84024	Del	
chr8:70999209-71033622	12	34414	Del	
chr8:95798679-95827942	5	29264	Del	DPY19L4
chr8:95798679-95829499	6	30821	Del	DPY19L4
chr8:98807085-98872905	10	65821	Del	LAPTM4B,MTDH
chr9:106747174-106766642	6	19469	Del	
chr9:113661675-113675515	7	13841	Del	
chr9:126992567-127162141	15	169575	Del	GAPVD1,HSPA5,RABEPK
chr9:131706896-131843027	17	136132	Del	FNBP1
chr9:133185787-133257282	17	71496	Del	
chr9:33407096-33419322	5	12227	Del	
chr9:33998406-34016538	8	18133	Del	UBAP2
chr9:33998406-34072144	13	73739	Del	UBAP2
chr9:4774933-4805648	11	30716	Del	RCL1
chr9:73884517-73923515	7	38999	Del	
chr10:104651474-	10	96226	Del	AS3MT,CNNM2
chr10:12068234-12116049	10	47816	Del	UPF2
chr10:12086552-12116049	8	29498	Del	UPF2
chr10:12332460-12363470	9	31011	Del	CDC123
chr10:12350743-12391095	11	40353	Del	
chr10:13325305-13339929	8	14625	Del	
chr10:15097380-15129986	12	32607	Del	OLAH
chr10:15097380-15136454	14	39075	Del	OLAH
chr10:16545723-16557790	7	12068	Del	PTER
chr10:21627502-21780646	22	153145	Del	
chr10:26863051-26897445	10	34395	Del	APBB1IP
chr10:65100822-65167843	17	67022	Del	
chr10:69797702-70017557	21	219856	Del	DNA2,RUFY2,SLC25A16,TET1
chr10:69859940-69933585	10	73646	Del	DNA2,SLC25A16
chr10:69859940-70280215	43	420276	Del	CCAR1,DNA2,SLC25A16,SNORD98,STOX1,TET1
chr10:76115212-76210993	18	95782	Del	ADK
chr10:76151317-76175577	6	24261	Del	
chr10:81784143-81837894	9	53752	Del	C10orf57,LOC219347
chr10:81801560-81881186	18	79627	Del	C10orf57,LOC219347
chr11:101350321-	7	38967	Del	KIAA1377
chr11:101466109-	5	29354	Del	YAP1
chr11:109730648-	7	37306	Del	
chr11:118189820-	11	50867	Del	
chr11:118210355-	8	30332	Del	
chr11:122460612-	16	45822	Del	ASAM
chr11:122481125-	15	44317	Del	ASAM
chr11:17257420-17277373	5	19954	Del	NUCB2
chr11:18528736-18584994	5	56259	Del	SPTY2D1,UEVLD
chr11:41413568-42223242	128	809675	Del	
chr11:42392810-42704558	66	311749	Del	
chr11:47859459-48063062	18	203604	Del	PTPRJ
chr11:77484811-77505604	9	20794	Del	ALG8
chr11:9273306-9320188	6	46883	Del	TMEM41B
chr11:97776889-97826974	12	50086	Del	

chr12:100328580-	20	101139	Del	SPIC
chr12:100409830-	12	44445	Del	
chr12:100801180-	6	13106	Del	DRAM1
chr12:117022062-	5	26409	Del	VSIG10
chr12:121805573-	8	72432	Del	CCDC62,DENR
chr12:127961067-	6	18015	Del	GLT1D1
chr12:32032686-32052364	11	19679	Del	C12orf35
chr12:32097827-32123489	9	25663	Del	
chr12:32871284-32886262	7	14979	Del	PKP2
chr12:47817725-47931507	7	113783	Del	TUBA1A
chr12:49079113-49127324	8	48212	Del	LARP4
chr12:49079113-49145087	11	65975	Del	LARP4
chr12:49100053-49145087	8	45035	Del	LARP4
chr12:55991898-56097681	7	105784	Del	
chr12:63368597-63377581	5	8985	Del	RASSF3
chr12:68270273-68294340	6	24068	Del	CCT2,LRRC10
chr12:864191-917792	9	53602	Dup	RAD52,WNK1
chr12:91853140-91872651	6	19512	Del	
chr12:94472225-94491588	9	19364	Del	
chr12:948155-971902	7	23748	Del	ERC1
chr12:97492358-97567338	16	74981	Del	APAF1,IKBIP,SLC25A3,SNORA53
chr13:20523473-20553335	6	29863	Del	LATS2
chr13:40107236-40228171	15	120936	Del	FOXO1,MIR320D1,MRPS31
chr13:82844883-82863121	6	18239	Del	
chr14:20820148-20840570	6	20423	Del	RPGRIP1
chr14:34039184-34274999	38	235816	Del	CFL2,EAPP,SNX6
chr14:34610515-34646534	6	36020	Del	FAM177A1,PPP2R3C
chr14:35148467-35222928	8	74462	Del	RALGAPA1
chr14:35945479-35971688	5	26210	Del	
chr14:47959934-48007525	11	47592	Del	
chr14:50438152-50478508	14	40357	Del	ABHD12B,PYGL
chr14:58050270-58097759	6	47490	Del	KIAA0586
chr14:63078562-63138559	7	59998	Del	PPP2R5E,WDR89
chr14:63078562-63226870	10	148309	Del	PPP2R5E,SGPP1,WDR89
chr14:63089400-63138559	6	49160	Del	WDR89
chr14:63089400-63243775	12	154376	Del	SGPP1,WDR89
chr14:73003728-73046687	9	42960	Del	C14orf169,HEATR4
chr14:73171175-73233378	7	62204	Del	DNAL1
chr14:73171175-73238184	8	67010	Del	DNAL1
chr15:32998973-33041888	7	42916	Del	AQR
chr15:39082307-39476524	40	394218	Del	CHP,EXD1,INO80,LOC729082,NDUFAF1,NUSAP1,OIP5
chr15:39171941-39317004	17	145064	Del	CHP,EXD1,INO80
chr15:39421879-39476524	7	54646	Del	NDUFAF1,NUSAP1
chr15:40997322-41112234	6	114913	Del	TTBK2,UBR1
chr15:42300382-42385934	6	85553	Del	CASC4
chr15:42300382-42462132	8	161751	Del	CASC4
chr15:48476200-48516566	6	40367	Del	USP8
chr15:48476200-48610580	16	134381	Del	USP50,USP8
chr15:53452605-53610894	21	158290	Del	CCPG1,DYX1C1
chr15:53475261-53547867	11	72607	Del	CCPG1,DYX1C1
chr15:53475261-53577602	15	102342	Del	CCPG1,DYX1C1
chr15:53480496-53547867	10	67372	Del	CCPG1,DYX1C1
chr15:60404485-60417907	5	13423	Del	
chr15:62352329-62721035	19	368707	Del	CSNK1G1,KIAA0101,TRIP4,ZNF609
chr15:62497880-62572511	6	74632	Del	TRIP4
chr15:63372790-63406850	6	34061	Del	IGDCC3
chr15:63543026-63656325	18	113300	Del	DPP8,PTPLAD1
chr15:63838399-63865123	5	26725	Del	DENND4A
chr15:73505722-73711498	11	205777	Del	PTPN9,SIN3A,SNUPN

chr15:73593966-73711498	7	117533	Del	PTPN9,SNUPN
chr15:88826834-88846423	5	19590	Del	IQGAP1
chr16:11924420-11968080	7	43661	Del	TNFRSF17
chr16:20924771-20953576	6	28806	Del	DNAH3
chr16:48606452-48712314	14	105863	Del	HEATR3,TMEM188
chr16:68017092-68051780	8	34689	Del	CYB5B
chr16:70318562-70559809	19	241248	Del	AP1G1,ATXN1L,KIAA0174,PKD1L3,SNORD71,ZNF821
chr16:70374400-70425669	5	51270	Del	AP1G1
chr16:70419634-70558637	12	139004	Del	ATXN1L,KIAA0174,PKD1L3,ZNF821
chr17:16038155-16227633	18	189479	Del	CENPV,MIR1288,NCOR1,PIGL,UBB
chr17:16088546-16259657	16	171112	Del	CENPV,MIR1288,PIGL,TRPV2,UBB
chr17:16088546-16266693	18	178148	Del	CENPV,MIR1288,PIGL,TRPV2,UBB
chr17:16136781-16162044	6	25264	Del	PIGL
chr17:16136781-16173942	7	37162	Del	PIGL
chr17:24793724-24859695	5	65972	Del	TAOK1
chr17:26138589-26250354	7	111766	Del	ATAD5,C17orf42,CRLF3
chr17:26160691-26191779	5	31089	Del	ATAD5,CRLF3
chr17:27219954-27269857	6	49904	Del	UTP6
chr17:34761298-34869863	6	108566	Del	FBXL20,MED1
chr17:54063018-54726214	36	663197	Del	C17orf71,GDPD1,MIR301A,MIR454,PPM1E,PRR11,RAD51C,SKA
chr17:55857642-56252870	24	395229	Del	APPBP2,BCAS3,C17orf64,PPM1D
chr17:55894285-56144864	15	250580	Del	APPBP2,BCAS3,PPM1D
chr17:62611701-62669917	8	58217	Del	HELZ
chr17:62642338-62740277	9	97940	Del	HELZ
chr17:63164976-63232361	7	67386	Del	NOL11,SNORA38B
chr17:63187880-63232361	5	44482	Del	
chr18:16954953-17049834	10	94882	Del	
chr18:17312540-17526315	11	213776	Del	ABHD3,ESCO1,GREB1L,MIR320C1,SNRPD1
chr18:20178701-20196570	10	17870	Del	OSBPL1A
chr18:54637618-54679876	5	42259	Del	
chr18:55649208-55658599	6	9392	Del	
chr18:55649208-55688923	11	39716	Del	
chr18:56202243-56210914	7	8672	Del	
chr18:59164885-59180491	7	15607	Del	KDSR
chr18:59167645-59180491	5	12847	Del	KDSR
chr18:6970523-7127068	76	156546	Del	LAMA1
chr19:16253163-16288607	8	35445	Del	
chr19:16253163-16300498	10	47336	Del	KLF2
chr19:37785092-37860843	12	75752	Del	ANKRD27,RGS9BP
chr19:41344025-41366905	8	22881	Del	ZNF565
chr19:41353729-41366905	7	13177	Del	ZNF565
chr19:50877990-50954126	13	76137	Del	FBXO46,QPCTL,SNRPD2
chr19:50880466-50954126	12	73661	Del	FBXO46,QPCTL,SNRPD2
chr19:62304065-62341712	11	37648	Del	USP29,ZIM3
chr19:8989937-9060226	10	70290	Del	
chr20:34970620-35070812	10	100193	Del	RBL1,SAMHD1
chr20:34970620-35151129	14	180510	Del	RBL1,SAMHD1
chr20:34984285-35070812	9	86528	Del	RBL1,SAMHD1
chr20:49892937-49899639	5	6703	Del	
chr20:50019338-50066879	15	47542	Del	
chr20:50052538-50119963	24	67426	Del	
chr21:26056693-26094576	5	37884	Del	GABPA
chr22:27369427-27486448	14	117022	Del	CHEK2,HSCB,TTC28
