# Supplementary material for: Age-Dependent Brain Gene Expression and Copy Number Anomalies in Autism Suggest Distinct Pathological Processes at Young Versus Mature Ages
Source: PLoS Genet. 2012 Mar 22;8(3):e1002592. doi: 10.1371/journal.pgen.1002592 (PMC3310790; doi:10.1371/journal.pgen.1002592)
Supplement: Table S9 — References for genes in noted pathways and networks. Important genes from the pathways and networks identified by gene expression analyses are listed. Gene category, references, and analysis corresponding to these genes are presented. Gex = gene expression. (PDF) [file pgen.1002592.s013.pdf]

Table S9. References for genes			
**Gene**	**Category**	**Reference**	**Analysis**
FAS	Apoptosis	Sancho-Martinez and Martin-Villalba 2009	Gex young vs. young
BCL3	Apoptosis	Brocke-Heidrich et al., 2006	Gex young vs. young
NOD1	Apoptosis	Jae Gyu et al., 2010	Gex young vs. young
FAT2	Apoptosis	Cavallaro et al., 2004	Gex young vs. young
PLP1	Apoptosis	Southwood and Gow, 2001	Gex young vs. young
CLN8	Apoptosis	Vantaggiato et al., 2009	Gex young vs. young
PTPRH	Apoptosis	Takada et al., 2002	Gex young vs. young
BAD	Apoptosis	Danial 2008	Gex diagnosis main effect
CASP8	Apoptosis	Frisch 2008	Gex diagnosis main effect
CASP10	Apoptosis	Muhlethaler-Mottet et al., 2011	Gex diagnosis main effect
MDM2	Apoptosis	Manfredi 2010	Gex diagnosis main effect
TSC22D3	Apoptosis	Soundararajan et al., 2007	Gex young vs. young
PPP1CA	Apoptosis, cell division and proliferation	Yu et al., 2008; Luo et al., 2007	Gex young vs. young
ROBO1	Axon guidance	Devine and Key 2008	Gex diagnosis main effect
NEK3	Cell cycle and division	Tanaka and Nigg, 1999	Gex young vs. young
BRCA1	Cell cycle and division	Wang 2007	Gex young vs. young
CHEK2	Cell cycle and division	Sato et al., 2010	Gex young vs. young
FANCB	Cell cycle and division	Wang 2007	Gex young vs. young
CHP2	Cell cycle and division	Cam et al., 2009	Gex young vs. young
CDC20	Cell cycle and division	Yu et al., 2007	Gex young vs. young
TRIOBP	Cell cycle and division	Yu et al., 2008	Gex young vs. young
ANKRD38	Cell cycle and division	Kakinuma et al., 2009	Gex young vs. young
FOSL2	Cell cycle and division	Maaser et al., 2008	Gex young vs. young
CHTF18	Cell cycle and division	Hanna et al., 2001	Gex young vs. young
CDC25	Cell cycle and division	Ivanova et al., 2011	Gex diagnosis main effect
ATRX	Cell cycle and division	De La Fuente et al., 2004	Gex diagnosis main effect
CTNNB1	Cell cycle and division	Chenn and Walsh 2002	Gex diagnosis main effect
p21	Cell cycle and division	Jung et al., 2010	Gex diagnosis main effect
14-3-3 (YWHAZ)	Cell cycle and division, neuronal migration	Toyo-oka et al., 2003; Darling et al., 2005	Gex diagnosis main effect
PTEN	Cell proliferation	Groszer et al., 2001; Butler et al., 2005; Page et al., 2009	Gex diagnosis main effect
PRKACB	Cell Proliferation	Stork and Schmitt, 2002; Cohen 2003; Tasken and Aandahl, 2004	Gex diagnosis main effect
PRKCZ	Cell Proliferation	Reyland 2009	Gex diagnosis main effect
TSC1	Cortical lamination and neuronal migration	Hay 2005; Orlova and Crino 2010	Gex diagnosis main effect
MMP2	Cytoskeleton and extracellular matrix remodeling	Lukes et al., 1999; Rauch 2004	Gex diagnosis main effect
NID1	Cytoskeleton and extracellular matrix remodeling	Miosge et al., 2001	Gex diagnosis main effect
TIMP1	Cytoskeleton and extracellular matrix remodeling	Lukes et al., 1999; Stamenkovic 2003	Gex diagnosis main effect
COL4A3	Cytoskeleton and extracellular matrix remodeling and Development	Zagris 2000	Gex diagnosis main effect

ErbB4	Cytoskeleton and extracellular matrix remodeling, development	Chong et al., 2008; Stamenkovic 2003	Gex diagnosis main effect
HGF	Development	Achim et al., 1997	Gex diagnosis main effect
FGFRL1	Development	Trueb and Taeschler 2006	Gex diagnosis main effect
ADORA2A	Development and apoptosis	Huang et al., 2001	Gex diagnosis main effect
FGF13	Neural patterning and differentiation	Nishimoto et al., 2007	Gex young vs. young
DLX4	Neural patterning and differentiation	Depew et al., 2005	Gex young vs. young
GREM1	Neural patterning and differentiation	Hsu et al., 1998	Gex young vs. young
NDE1	Neural patterning and differentiation	Feng and Walsh 2004	Gex young vs. young
NODAL	Neural patterning and differentiation	Schier et al., 2003	Gex young vs. young
HOXD1	Neural patterning and differentiation	Zakany et al., 2001	Gex young vs. young
PCSK6	Neural patterning and differentiation	Constam and Robertson, 2000	Gex young vs. young
NTRK3	Neural patterning and differentiation	Bartkowska et al., 2007	Gex young vs. young
POU6F2	Neural patterning and differentiation	Zhou et al., 1996	Gex young vs. young
WNT3	Neural patterning and differentiation	Lee et al., 2000	Gex young vs. young
HYDIN	Neurodevelopment	Dawe et al., 2007	Gex young vs. young
RELN	Neuronal migration	Tissir and Goffinet 2003	Gex diagnosis main effect
PAK1	Neuronal migration	Causeret et al., 2009	Gex diagnosis main effect
