# Supplementary material for: Age-Dependent Brain Gene Expression and Copy Number Anomalies in Autism Suggest Distinct Pathological Processes at Young Versus Mature Ages
Source: PLoS Genet. 2012 Mar 22;8(3):e1002592. doi: 10.1371/journal.pgen.1002592 (PMC3310790; doi:10.1371/journal.pgen.1002592)
Supplement: Text S1 — Supporting materials. Supplementary methods, acknowledgements and figures are provided. (DOCX) [file pgen.1002592.s014.docx]

**Text S1**

**Supporting Materials**

**for**

**Age Dependent Brain Gene Expression and Copy Number Anomalies in Autism Suggest Distinct Pathological Processes at Young Versus Mature Ages**

Maggie L Chow*, Tiziano Pramparo*, Mary E Winn, Cynthia Carter Barnes, Hai-Ri Li, Lauren Weiss, Jian-Bing Fan, Sarah Murray, Craig April, Haim Belinson, Xiang-Dong Fu**, Anthony Wynshaw-Boris**, Nicholas J Schork**, Eric Courchesne**

* = co-1st authors

** = co-senior authors

**Supplementary Methods**

**Gene Expression Analysis**

**Cases**

Fifty-seven frozen, postmortem, cortical tissue blocks (1-2 cc) of prefrontal cortex from young autistic and control cases aged 2-56 years were obtained from the National Institute of Child Health and Human Development, University of Maryland brain bank and from the Autism Tissue Program. All autistic cases met criteria for autistic disorder on the Autism Diagnostic Interview-Revised (1), the Autism Diagnostic Observation Schedule (2), or were diagnosed by medical records (Table S1). Research procedures employed in this study were approved by the IRB of UC San Diego (protocol number 091205).

**Brain Sample Collection**

Due to documented variability of neighboring brain areas (3,4), care was taken to ensure tissue blocks were from comparable regions using anatomical landmarks. When available, tissue from the superior frontal gyrus of the dorsal lateral prefrontal cortex (DLPFC; approximately BA9/46) was dissected in each case. When this area was not available, we sampled from the middle frontal gyrus.

**DASL-based Labeling, Hybridization, and Scanning**

Given the difficulties surrounding expression profiling of partially degraded RNA, we used the DNA annealing, selection, and ligation (DASL)-based approach for gene expression profiling (5), which is efficacious even with formalin-fixed, paraffin-embedded tissue (6,7). All available samples were processed given that RNA Integrity Numbers (RINs) are not predictive of the utility of RNA samples in DASL experiments (8).

**Exclusion Criteria**

Detailed quality control and data preprocessing procedures are published elsewhere (14). Four autistic and 4 control female cases passed all quality control checks and had high quality microarray results. 5 female cases were excluded according to criteria below. However, due to the discrepancy in ages between useable autistic (4, 7, 18, 49 years) and control female (4, 4, 6, 9 years) cases, these cases were excluded from final expression profiling analyses.

Of the remaining all-male cases, 11 were excluded due to poor quality gene expression hybridization based on quality control using visualizations from the *lumi* package in Bioconductor (9,10). Several criteria were considered to identify possible outliers. Samples were eliminated due to low median distribution amplitudes and because the range or interquartile range of intensity values were visually different from the remainder of the arrays. Samples were also eliminated due to poor correlations with other samples based on “distance from center” outlier detection methods in the *lumi* package. All samples with average clustering more disparate than the distance between the two batches were removed. We confirmed these choices with analysis of pair-wise correlations between biological and technical replicates using several visualization techniques and multivariate distance matrix regression (11). Further, according to outlier removal procedures from Oldham et al. (12), the mean normalized inter-array correlation (IAC) of the remaining borderline outlying samples was maximized for the dataset. Finally, we performed all preprocessing steps, differential expression and enrichment analysis including and excluding borderline outliers to ensure that inclusion of single cases could not account for the reported results. The final dataset consisted of 33 high quality arrays.

**Data Preprocessing**

Raw data exported from Illumina’s BeadStudio software underwent outlier removal, log2 transformation and quantile normalization as implemented in the *lumi* package for R/Bioconductor (10). Samples were processed at two different time points, and average linkage hierarchical clustering revealed that batch effects were present and required removal by statistical tools. Additionally, some of the autistic cases had a history of seizures, creating potential confounds to the data. To simultaneously remove variance attributed to batch and seizures, we performed batch and covariate correction using ComBat (13). Multivariate Distance Matrix Regression (11) with 10,000 permutations confirmed that variance attributable to batch and seizures were decreased following correction. Detailed information regarding the methodology for normalizing gene expression data and removing outliers is provided in Chow et al. (14).

**Enrichment Analysis**

In MetaCore, pathways are defined as sets of linear consecutive signals or metabolic transformations that have been confirmed as a whole by inferred relationships or experimental data. Map Folders are comprised of three to five M-Pathways and divided into regulatory, metabolic, disease, toxicity and drug action sections. GeneGO network processes are network models of main cellular processes that are created manually by GeneGO using GO processes and GeneGO pathway maps.

Differentially affected pathways were generated using the ‘compare experiments’ option. MetaCore Network Analysis was performed using the differentially expressed genes listed in each functional domain in the fold-change graphs. The shortest path algorithm with 2 genes as the maximum number of steps in the path was selected.

# Copy Number Variation (CNV) Analysis

Extraction of genomic DNA from 5-10 mg of frozen brain tissue from grey and white matter from 55 cases was performed using the QiaAMP DNA micro kit according to manufacturer’s protocol. gDNA from each sample was quantified using a NanoDrop® spectrophotometer. These samples were genotyped on the Illumina® 660 Bead Array (Illumina Inc., San Diego, California) platform according to the manufacturer’s protocol. SNP calls were obtained using the Illumina® Genome Studio software and the Illumina Human660W manifest file for normalization and genotype clustering. Cnvi probes and zeroed SNPs were excluded from the final reports. Samples with call rates below 97% or with sex mismatch were removed before CNV analysis. QC parameters used during the CNV detection are described below.

CNV calls were first performed using the PennCNV software (http://www.openbioinformatics.org/penncnv/) implementing the wave adjustment procedure via the “–gcmodel” argument (15). Thirty cases (14 autism and 16 controls) passed the software default quality control measures for CNV calls based on the standard deviation of the Log Ratio of allele intensities (LRR_SD <0.25) across all calls and the Waving Factor (-0.04<WF <0.04).

We first analyzed only male cases (12 autism and 12 control; Figure 4A and 4B) and identified a total of about 850 CNVs (>5 consecutive SNPs and >5 kb in size). Known nonpathogenic regions reported in the Database of Genomic Variants (<http://projects.tcag.ca/cgi-bin/variation/gbrowse/hg18>) were filtered without upper size limits. Filtering occurred when two overlapping CNV regions shared at least 50% of their sequences. No large-size CNVs (>1Mb) were identified in single cases that could potentially drive the gene enrichment results.

We next analyzed male and female cases together (14 autism and 16 controls; Figure S4A) using the same parameters. We identified a total of about 1300 CNVs (>5 consecutive SNPs and >5 kb in size) and then proceeded with the filtering of common regions. To exclude the possibility of biased CNV detection given the small sample size, we compared the CNV results between autism cases and controls for number, distribution and size of the predicted regions (Figure S4B-F). We found no significant difference in the average number of CNVs, gene content of CNVs or the average size of CNVs between autism cases and controls. The size distribution of CNVs was less than 1 Mb (Figure S4B-F). Thus, there was no obvious bias in the CNV parameters between autism cases and controls.

Due to possible high false positive rates, we re-analyzed all cases using CNVision, a recently described analysis pipeline (16) ([www.cnvision.org](http://www.cnvision.org/)) that merges the results of PennCNV, QuantiSNP and GNOSIS. PennCNV and QuantiSNP use a Hidden Markov Model algorithm for CNV prediction. GNOSIS implements an algorithm based on sliding windows that use the logR and BAF thresholds determined from Illumina SNP data. Within this pipeline, quality control for each software are as follow: GNOSIS looks for outliers using call rates>95%; PennCNV uses LRR_SD<0.28, B allele frequency drift (BAF_Drift)<0.01 and WF within 0.05 and -0.05; QuantiSNP uses BAF≤0.1, LRR_SD≤0.4, BAF_SD≤0.2 and LRR_outliers≤0.1.

Final CNV calls were selected using high confidence thresholds: >50% calls detected with both PennCNV and QuantiSNP algorithms gave 91% of positive predicted value (PPV) and >50% calls detected with all three algorithms yielded 95% of PPV. The filtering of common CNVs was performed as described above. The analysis of the male cases yielded about 350 CNVs (11 autism and 13 controls). Gene enrichment was performed considering both the gene content of gene-rich CNVs and the nearest gene at the 5- and 3-prime end of gene-desert CNVs. As performed with PennCNV alone, we next analyzed male and female cases together. However, we were unable to compare the gene enrichment of the two categories due to the significant difference in the number of cases that passed QC (12 autism versus 19 controls).

GO enrichment of the gene content of these regions was also performed using the MetaCore software suite (FDR<0.01 and FDR<0.05). GeneGO Network Processes of each of these gene lists are reported in Figures 4 and S4.

# Gene Association Analysis Using Autism Genetic Datasets

We utilized the AGRE-NIMH Broad/Johns Hopkins Medical Institute (Broad/JHMI) sample as an experimental dataset using original quality control filters and the Children’s Hospital of Philadelphia (CHOP) sample dataset as a replication sample, as previously described (17). Genotyping QC methods to clean the association datasets are described in Weiss et al., 2009. AGRE SNP data described in Wang et al. (18) were filtered as follows: 1) SNPs missing >10% data were excluded; 2) individuals missing >10% data were excluded; 3) SNPs with HWE p<0.001 were excluded; 4) SNPs with AF<0.01 were excluded; 5) SNPs with >10 Mendelian errors were excluded; 6) mis-identified and overlapping subjects with the Weiss et al. (17) dataset were excluded.

Genes annotated in Gene Ontology (GO; [http://www.geneontology.org/](http://www.geneontology.org/)) and GeneGO (<http://www.genego.com/metacore.php>) databases as being involved in cell cycle (and different phases of the cell cycle), growth factor, synaptogenesis, apoptosis and inflammatory processes were isolated for analysis using the Ingenuity® software suite, while the Wnt pathway genes and targets were identified from the Wnt homepage (http://www.stanford.edu/group/nusselab/cgi-bin/wnt/).

We used PLINK (19) (<http://pngu.mgh.harvard.edu/~purcell/plink/>) to retrieve the SNPs within a window of 20kb around each gene and to perform set-based association analysis. The family-based transmission disequilibrium test (TDT) used for association in this data is not biased by population stratification. The set-based test allows permutations for each gene-set and correction for the multiple SNPs within each set. However, the p-values reported here have not been corrected for the multiple sets tested.

The set-based analysis was performed as follows: 1) for each SNP in a set, LD was determined with other SNPs and a threshold of r^2^=0.5 was set for independence; 2) family-based TDT was then performed; 3) for each set, all independent SNPs were selected with p-values of p=0.05 and below in descending order according to statistical significance after removal of SNPs in LD with previously selected SNPs; 4) the statistic of the set was the mean of the selected single SNP statistics; 5) permutation of the dataset was performed 10,000 times, keeping LD between SNPs constant by permuting phenotype labels; 6) for each permuted dataset, steps 2 to 4 were repeated; 7) the empirical p-value for a set was the proportion of times that the permutation p-value exceeded the original one for that set. We did not perform correction for the number of sets tested.

Significantly associated pathways (p<0.05) in the cell cycle gene set were then broken down into twenty-one subsets of genes denoting specific phases of the cell cycle using Ingenuity and set-based tests were again performed on the experimental dataset. Subsets that were found to be significant in the Broad/JHMI sample were then tested on the CHOP sample for replication using the same set-based analysis.

**Supplementary Acknowledgements**

We send our appreciation to all parents who made the difficult choice to support brain research through the donation of brain tissue from their loved ones. Tissue for this study was provided by the National Institute of Child Health and Human Development Brain and Tissue Bank for Developmental Disorders (Baltimore, MD) under contracts N01-HD-4-3368 and N01-HD-4-3383, the Brain and Tissue Bank for Developmental Disorders (Miami, FL), Autism Tissue Program (Princeton, NJ) and Harvard Brain Tissue Resource Center (Belmont, MA). We thank Dr. Ronald Zielke at the National Institute of Child Health and Human Development Brain and Tissue Bank for Developmental Disorders and Dr. Jane Pickett at the Autism Tissue Program for facilitation of tissue acquisition and Dr. Joeseph Buckwalter, Dr. Cynthia Schumann, Robert Johnson and Robert Vigorito for help in tissue dissection and collection. We also thank Stephan J. Sanders at the Child Study Center, Yale University for providing the up-to-date CNVision software and support in bringing it up at our facility and Joseph Shieh at the University of California San Francisco for his assistance with the CNV analysis. Insightful comments on the manuscript were provided by Dr. Karen Pierce. Dr. Jian-Bing Fan and Dr. Craig April declare stock and employment interest in Illumina, Inc.

**Acknowledgements for Autism Biomaterials and Clinical Data**The collection of data and biomaterials in one project that participated in the National Institute of Mental Health (NIMH) Autism Genetics Initiative has been supported by National Institute of Health grants MH52708, MH39437, MH00219, and MH00980; National Health Medical Research Council grant 0034328; and by grants from the Scottish Rite, the Spunk Fund, Inc., the Rebecca and Solomon Baker Fund, the APEX Foundation, the National Alliance for Research in Schizophrenia and Affective Disorders (NARSAD), the endowment fund of the Nancy Pritzker Laboratory (Stanford); and by gifts from the Autism Society of America, the Janet M. Grace Pervasive Developmental Disorders Fund, and families and friends of individuals with autism. The Principal Investigators and Co-Investigators were: Stanford University, Stanford: Neil Risch, Ph.D., Richard M. Myers, Ph.D., Donna Spiker, Ph.D., Linda J. Lotspeich, M.D., Joachim Hallmayer, M.D., Helena C. Kraemer, Ph.D., Roland D. Ciaranello, M.D., Luca L. Cavalli-Sforza, M.D., University of Utah, Salt Lake City: William M. McMahon, M.D. and P. Brent Petersen. The Stanford team is indebted to the parent groups and clinician colleagues who referred families. The Stanford team extends our gratitude to the families with individuals with autism who were our partners in this research.

The collection data and biomaterials also come from the Autism Genetic Resource Exchange (AGRE) collection. This program has been supported by a National Institute of Health (grants MH64547 and 1U24MH081810) and Autism Speaks, Inc. (formerly the Cure Autism Now Foundation). The Principal Investigator of grant MH64547 is Daniel H. Geschwind, M.D., Ph.D. (UCLA). The Co-Principal Investigators include Stanley F. Nelson, M.D., and Rita Cantor, Ph.D. (UCLA), Christa Lese Martin, Ph.D. (U. Chicago), T. Conrad Gilliam, Ph.D. (Columbia). Co-investigators include Maricela Alarcon, Ph.D., Kenneth Lange, Ph.D., Sarah J. Spence M.D., Ph.D. (UCLA), David H. Ledbetter Ph.D. (Emory) and Hank Juo, M.D., Ph.D. (Columbia).

The Principal Investigator of grant 1U24MH081810 is Clara M. Lajonchere, Ph.D. (USC). The Co-Principal Investigators include Steven Moldin, Ph.D. (USC), Janet Miller, J.D., Ph.D. (Autism Speaks), Mark Urata, M.D. (CHLA), Constantinos Sioutas, Ph.D. (USC), David Amaral, Ph.D. (UC Davis), Curtis Deutsch, Ph.D. (UMASS).

Scientific oversight of the AGRE program is provided by the AGRE steering committee: Dan Geschwind, M.D., Ph.D., UCLA; Maja Bucan, Ph.D., University of Pennsylvania; W. Ted Brown, M.D., Ph.D., F.A.C.M.G., N.Y.S. Institute for Basic Research in Developmental Disabilities; Rita M. Cantor, Ph.D., UCLA; John N. Constantino, M.D., Washington University School of Medicine, St. Louis; T. Conrad Gilliam, Ph.D., University of Chicago; Martha Herbert, M.D., Ph.D., Harvard Medical School; Clara Lajonchere, Ph.D., Autism Speaks; David H. Ledbetter, Ph.D., Emory University; Christa Lese-Martin, Ph.D., Emory University; Janet Miller, J.D., Ph.D., Autism Speaks; Stanley F. Nelson, M.D., UCLA; Gerard D. Schellenberg, Ph.D., University of Pennsylvania; Carol A. Samanago-Sprouse, Ed.D., George Washington University; Sarah Spence, M.D., Ph.D., NIMH; Matthew State, M.D., Ph.D., Yale University; Rudolph E. Tanzi, Ph.D., Massachusetts General Hospital.

**References**

1. Lord C, Rutter M, Couteur A. Autism Diagnostic Interview-Revised: A revised version of a diagnostic interview for caregivers of individuals with possible pervasive developmental disorders. Journal of Autism and Developmental Disorders [Internet]. 1994 Oct [cited 2010 Oct 18];24(5):659–85. Available from: http://www.springerlink.com/content/w362007746715g45/

2. Lord C, Risi S, Lambrecht L, Cook EH, Leventhal BL, DiLavore PC, et al. The Autism Diagnostic Observation Schedule—Generic: A Standard Measure of Social and Communication Deficits Associated with the Spectrum of Autism [Internet]. Springer Netherlands; 2000 [cited 2010 Oct 18]. Available from: http://www.springerlink.com/content/m1227k1w710w6874/

3. Rehen SK, Yung YC, McCreight MP, Kaushal D, Yang AH, Almeida BSV, et al. Constitutional aneuploidy in the normal human brain. The Journal of neuroscience : the official journal of the Society for Neuroscience [Internet]. 2005 Mar [cited 2010 Oct 1];25(9):2176–80. Available from: http://www.jneurosci.org/cgi/content/abstract/25/9/2176

4. Lein ES, Hawrylycz MJ, Ao N, Ayres M, Bensinger A, Bernard A, et al. Genome-wide atlas of gene expression in the adult mouse brain. Nature [Internet]. 2007 Jan;445(7124):168–76. Available from: http://www.ncbi.nlm.nih.gov/pubmed/17151600

5. Chow ML, Li H-R, Winn ME, April C, Barnes CC, Wynshaw-Boris A, et al. Genome-wide expression assay comparison across frozen and fixed postmortem brain tissue samples. BMC genomics [Internet]. 2011 Jan [cited 2011 Oct 9];12:449. Available from: http://www.pubmedcentral.nih.gov/articlerender.fcgi?artid=3179967&tool=pmcentrez&rendertype=abstract

6. April C, Klotzle B, Royce T, Wickham-Garcia E, Boyaniwsky T, Izzo J, et al. Whole-genome gene expression profiling of formalin-fixed, paraffin-embedded tissue samples. PloS one [Internet]. 2009 Jan [cited 2011 Mar 17];4(12):e8162. Available from: http://www.pubmedcentral.nih.gov/articlerender.fcgi?artid=2780295&tool=pmcentrez&rendertype=abstract

7. Fan J-B, Yeakley JM, Bibikova M, Chudin E, Wickham E, Chen J, et al. A versatile assay for high-throughput gene expression profiling on universal array matrices. Genome research [Internet]. 2004 May;14(5):878–85. Available from: http://www.ncbi.nlm.nih.gov/pubmed/15123585

8. Abramovitz M, Ordanic-Kodani M, Wang Y, Li Z, Catzavelos C, Bouzyk M, et al. Optimization of RNA extraction from FFPE tissues for expression profiling in the DASL assay. BioTechniques [Internet]. 2008 Mar [cited 2010 Oct 1];44(3):417–23. Available from: http://www.pubmedcentral.nih.gov/articlerender.fcgi?artid=2672087&tool=pmcentrez&rendertype=abstract

9. Gentleman RC, Carey VJ, Bates DM, Bolstad B, Dettling M, Dudoit S, et al. Bioconductor: open software development for computational biology and bioinformatics. Genome biology [Internet]. 2004 Jan [cited 2010 Oct 19];5(10):R80. Available from: http://www.pubmedcentral.nih.gov/articlerender.fcgi?artid=545600&tool=pmcentrez&rendertype=abstract

10. Du P, Kibbe W a, Lin SM. lumi: a pipeline for processing Illumina microarray. Bioinformatics (Oxford, England) [Internet]. 2008 Jul;24(13):1547–8. Available from: http://www.ncbi.nlm.nih.gov/pubmed/18467348

11. Zapala MA, Schork NJ. Multivariate regression analysis of distance matrices for testing associations between gene expression patterns and related variables. Proceedings of the National Academy of Sciences of the United States of America [Internet]. 2006 Dec [cited 2010 Aug 12];103(51):19430–5. Available from: http://www.pnas.org/cgi/content/abstract/103/51/19430

12. Oldham MC, Konopka G, Iwamoto K, Langfelder P, Kato T, Horvath S, et al. Functional organization of the transcriptome in human brain. Nature neuroscience [Internet]. 2008 Nov [cited 2010 Oct 1];11(11):1271–82. Available from: http://dx.doi.org/10.1038/nn.2207

13. Johnson WE, Li C, Rabinovic A. Adjusting batch effects in microarray expression data using empirical Bayes methods. Biostatistics (Oxford, England) [Internet]. 2007 Jan [cited 2010 Oct 1];8(1):118–27. Available from: http://biostatistics.oxfordjournals.org/cgi/content/abstract/8/1/118

14. Chow M, Winn M, Li H-R, April C, Barnes CC, Wynshaw-Boris A, et al. (*in press)* Genome-wide Brain Gene Expression Microarray Data Preprocessing and Quality Control Scheme using the Illumina DASL Assay.

15. Wang K, Li M, Hadley D, Liu R, Glessner J, Grant SFA, et al. PennCNV: an integrated hidden Markov model designed for high-resolution copy number variation detection in whole-genome SNP genotyping data. Genome research [Internet]. 2007 Nov [cited 2011 Feb 24];17(11):1665–74. Available from: http://www.pubmedcentral.nih.gov/articlerender.fcgi?artid=2045149&tool=pmcentrez&rendertype=abstract

16. Sanders SJ, Ercan-Sencicek AG, Hus V, Luo R, Murtha MT, Moreno-De-Luca D, et al. Multiple Recurrent De Novo CNVs, Including Duplications of the 7q11.23 Williams Syndrome Region, Are Strongly Associated with Autism. Neuron [Internet]. 2011 Jun 9 [cited 2011 Jun 13];70(5):863–85. Available from: http://www.ncbi.nlm.nih.gov/pubmed/21658581

17. Weiss LA, Arking DE, Daly MJ, Chakravarti A. A genome-wide linkage and association scan reveals novel loci for autism. Nature [Internet]. 2009 Oct;461(7265):802–8. Available from: http://www.ncbi.nlm.nih.gov/pubmed/19812673

18. Wang K, Zhang H, Ma D, Bucan M, Glessner JT, Abrahams BS, et al. Common genetic variants on 5p14.1 associate with autism spectrum disorders. Nature [Internet]. 2009 May;459(7246):528–33. Available from: http://www.ncbi.nlm.nih.gov/pubmed/19404256

19. Purcell S, Neale B, Toddbrown K, Thomas L, Ferreira M, Bender D, et al. PLINK: A Tool Set for Whole-Genome Association and Population-Based Linkage Analyses. The American Journal of Human Genetics [Internet]. 2007 Sep [cited 2010 Aug 19];81(3):559–75. Available from: http://www.pubmedcentral.nih.gov/articlerender.fcgi?artid=1950838&tool=pmcentrez&rendertype=abstract

**Table S9 references:**

Achim C. Expression of HGF and cMet in the developing and adult brain. Developmental Brain Research. 1997 Sep ;102(2):299-303.

Anney R, Klei L, Pinto D, Regan R, Conroy J, Magalhaes TR, et al. A genomewide scan for common alleles affecting risk for autism. Human molecular genetics. 2010 Jul ;19(20):4072-82.

Arking DE, Cutler DJ, Brune CW, Teslovich TM, West K, Ikeda M, et al. A common genetic variant in the neurexin superfamily member CNTNAP2 increases familial risk of autism. American journal of human genetics. 2008 Jan ;82(1):160-4.

Auranen M, Nieminen T, Majuri S, Vanhala R, Peltonen L, Jarvela I. Analysis of autism susceptibility gene loci on chromosomes 1p, 4p, 6q, 7q, 13q, 15q, 16p, 17q, 19q and 22q in Finnish multiplex families Molecular psychiatry. 2000 ;5(3):320-322.

Auranen M, Vanhala R, Varilo T, Ayers K, Kempas E, Ylisaukko-Oja T, et al. A genomewide screen for autism-spectrum disorders: evidence for a major susceptibility locus on chromosome 3q25-27. American journal of human genetics. 2002 Oct ;71(4):777-90.

Bacchelli E, Blasi F, Biondolillo M, Lamb JA, Bonora E, Barnby G, et al. Screening of nine candidate genes for autism on chromosome 2q reveals rare nonsynonymous variants in the cAMP-GEFII gene. Molecular psychiatry. 2003 Nov ;8(11):916-24.

Bakkaloglu B, O'Roak BJ, Louvi A, Gupta AR, Abelson JF, Morgan TM, et al. Molecular cytogenetic analysis and resequencing of contactin associated protein-like 2 in autism spectrum disorders. American journal of human genetics. 2008 Jan ;82(1):165-73.

Barnby G, Abbott A, Sykes N, Morris A, Weeks DE, Mott R, et al. Candidate-gene screening and association analysis at the autism-susceptibility locus on chromosome 16p: evidence of association at GRIN2A and ABAT. American journal of human genetics. 2005 Jun ;76(6):950-66.

Bartkowska K, Paquin A, Gauthier AS, Kaplan DR, Miller FD. Trk signaling regulates neural precursor cell proliferation and differentiation during cortical development.Development (Cambridge, England). 2007 Dec ;134(24):4369-80.

Ben-Shachar S, Lanpher B, German JR, Qasaymeh M, Potocki L, Nagamani SCS, et al. Microdeletion 15q13.3: a locus with incomplete penetrance for autism, mental retardation, and psychiatric disorders. Journal of medical genetics. 2009 Jun ;46(6):382-8.

Blasi F, Bacchelli E, Carone S, Toma C, Monaco AP, Bailey AJ, et al. SLC25A12 and CMYA3 gene variants are not associated with autism in the IMGSAC multiplex family sample. European journal of human genetics : EJHG. 2006 Jan ;14(1):123-6.

Brocke-Heidrich K, Ge B, Cvijic H, Pfeifer G, Löffler D, Henze C, et al. BCL3 is induced by IL-6 via Stat3 binding to intronic enhancer HS4 and represses its own transcription. Oncogene. 2006 Nov ;25(55):7297-304.

Brune CW, Korvatska E, Allen-Brady K, Cook EH, Dawson G, Devlin B, et al. Heterogeneous association between engrailed-2 and autism in the CPEA network. American journal of medical genetics. Part B, Neuropsychiatric genetics : the official publication of the International Society of Psychiatric Genetics. 2008 Mar ;147B(2):187-93.

Buxbaum JD, Silverman J, Keddache M, Smith CJ, Hollander E, Ramoz N, et al. Linkage analysis for autism in a subset families with obsessive-compulsive behaviors: evidence for an autism susceptibility gene on chromosome 1 and further support for susceptibility genes on chromosome 6 and 19. Molecular psychiatry. 2004 Feb ;9(2):144-50.

Cam HP, Chen ES, Grewal SIS. Transcriptional scaffolds for heterochromatin assembly. Cell. 2009 Feb ;136(4):610-4.

Cantor RM, Kono N, Duvall JA, Alvarez-Retuerto A, Stone JL, Alarcón M, et al. Replication of autism linkage: fine-mapping peak at 17q21. American journal of human genetics. 2005 Jun ;76(6):1050-6.

Causeret F, Terao M, Jacobs T, Nishimura YV, Yanagawa Y, Obata K, et al. The p21-activated kinase is required for neuronal migration in the cerebral cortex. Cerebral cortex (New York, N.Y. : 1991). 2009 Apr ;19(4):861-75.

Cavallaro S, D'Agata V, Alessi E, Coffa S, Alkon DL, Manickam P, et al. Gene expression profiles of apoptotic neurons. Genomics. 2004 Sep ;84(3):485-96.

Chong VZ, Webster MJ, Rothmond DA, Weickert CS. Specific developmental reductions in subventricular zone ErbB1 and ErbB4 mRNA in the human brain. International journal of developmental neuroscience : the official journal of the International Society for Developmental Neuroscience. 2008 Nov ;26(7):791-803.

Cohen MM. The hedgehog signaling network. American journal of medical genetics. Part A. 2003 Nov ;123A(1):5-28.

Constam DB, Robertson EJ. SPC4/PACE4 regulates a TGFbeta  signaling network during axis formation Genes &amp; Dev. 2000 ;14(9):1146-1155.

Cook EH, Courchesne R, Lord C, Cox NJ, Yan S, Lincoln A, et al. Evidence of linkage between the serotonin transporter and autistic disorder. Molecular psychiatry. 1997 May ;2(3):247-50.

Danial NN. BAD: undertaker by night, candyman by day. Oncogene. 2008 Dec ;27 Suppl 1S53-70.

Darling D, Yingling J, Wynshaw-Boris A. Role of 14–3–3 Proteins in Eukaryotic Signaling and Development Current Topics in Developmental Biology. 2005 ;68281-315.

Dawe HR, Shaw MK, Farr H, Gull K. The hydrocephalus inducing gene product, Hydin, positions axonemal central pair microtubules. BMC biology. 2007 Jan ;533.

De La Fuente R, Viveiros MM, Wigglesworth K, Eppig JJ. ATRX, a member of the SNF2 family of helicase/ATPases, is required for chromosome alignment and meiotic spindle organization in metaphase II stage mouse oocytes. Developmental biology. 2004 Aug ;272(1):1-14.

Depew MJ, Simpson CA, Morasso M, Rubenstein JLR. Reassessing the Dlx code: the genetic regulation of branchial arch skeletal pattern and development. Journal of anatomy. 2005 Nov ;207(5):501-61.

Devine CA, Key B. Robo-Slit interactions regulate longitudinal axon pathfinding in the embryonic vertebrate brain. Developmental biology. 2008 Jan ;313(1):371-83.

Durand CM, Betancur C, Boeckers TM, Bockmann J, Chaste P, Fauchereau F, et al. Mutations in the gene encoding the synaptic scaffolding protein SHANK3 are associated with autism spectrum disorders. Nature genetics. 2007 Jan ;39(1):25-7.

Fei P, Yin J, Wang W. New Advances in the DNA Damage Response Network of Fanconi Anemia and BRCA proteins: FAAP95 Replaces BRCA2 as the True FANCB Protein Cell Cycle. 2005 Jan ;4(1):80-86.

Feng Y, Walsh CA. Mitotic spindle regulation by Nde1 controls cerebral cortical size. Neuron. 2004 Oct ;44(2):279-93.

Freitag CM, Staal W, Klauck SM, Duketis E, Waltes R. Genetics of autistic disorders: review and clinical implications. European child & adolescent psychiatry. 2010 Mar ;19(3):169-78.

Freitag CM. The genetics of autistic disorders and its clinical relevance: a review of the literature. Molecular psychiatry. 2007 Jan ;12(1):2-22.

Frisch SM. Caspase-8: fly or die. Cancer research. 2008 Jun ;68(12):4491-3.

Hanna JS, Kroll ES, Lundblad V, Spencer FA. Saccharomyces cerevisiae CTF18 and CTF4 are required for sister chromatid cohesion. Molecular and cellular biology. 2001 May ;21(9):3144-58.

Herman GE, Butter E, Enrile B, Pastore M, Prior TW, Sommer A. Increasing knowledge of PTEN germline mutations: Two additional patients with autism and macrocephaly.American journal of medical genetics. Part A. 2007 Mar ;143(6):589-93.

Hsu D, Economides A, Wang X, Eimon P, Harland R. The Xenopus Dorsalizing Factor Gremlin Identifies a Novel Family of Secreted Proteins that Antagonize BMP Activities Molecular Cell. 1998 Apr ;1(5):673-683.

Huang NK, Lin YW, Huang CL, Messing RO, Chern Y. Activation of protein kinase A and atypical protein kinase C by A(2A) adenosine receptors antagonizes apoptosis due to serum deprivation in PC12 cells. The Journal of biological chemistry. 2001 Apr ;276(17):13838-46.

IMGSAC. A genome wide screen for autism: Strong evidence for linkage to chromosomes 2q, 7q and 16p. Am. J. Hum. Genet. 2001 ;69570-581.

Ingram JL, Stodgell CJ, Hyman SL, Figlewicz DA, Weitkamp LR, Rodier PM. Discovery of allelic variants ofHOXA1 andHOXB1: Genetic susceptibility to autism spectrum disorders Teratology. 2000 Dec ;62(6):393-405.

International Molecular Genetic Study of Autism Consortium. A full genome screen for autism with evidence for linkage to a region on chromosome 7q. International Molecular Genetic Study of Autism Consortium Human Molecular Genetics. 1998 Mar ;7(3):571-578.

Itsara A, Cooper GM, Baker C, Girirajan S, Li J, Absher D, et al. Population analysis of large copy number variants and hotspots of human genetic disease. American journal of human genetics. 2009 Feb ;84(2):148-61.

Ivanova T, Gómez-Escoda B, Hidalgo E, Ayté J. G 1/S transcription and the DNA synthesis checkpoint: Common regulatory mechanisms. Cell cycle (Georgetown, Tex.). 2011 Mar ;10(6):

Jacob S, Brune CW, Carter CS, Leventhal BL, Lord C, Cook EH. Association of the oxytocin receptor gene (OXTR) in Caucasian children and adolescents with autism. Neuroscience letters. 2007 Apr ;417(1):6-9.

Jae Gyu K, Sung Joong L, Kagnoff MF. Nod1 is an essential signal transducer in intestinal epithelial cells infected with bacteria that avoid recognition by toll-like receptors Infection and immunity. 2010 ;72(3):1487-1495.

Jamain S, Betancur C, Quach H, Philippe A, Fellous M, Giros B, et al. Linkage and association of the glutamate receptor 6 gene with autism. Molecular psychiatry. 2002 Jan ;7(3):302-10.

Jamain S, Quach H, Betancur C, Råstam M, Colineaux C, Gillberg IC, et al. Mutations of the X-linked genes encoding neuroligins NLGN3 and NLGN4 are associated with autism. Nature genetics. 2003 May ;34(1):27-9.Available from: http://www.ncbi.nlm.nih.gov/pubmed/12669065

Junaid MA, Kowal D, Barua M, Pullarkat PS, Sklower Brooks S, Pullarkat RK. Proteomic studies identified a single nucleotide polymorphism in glyoxalase I as autism susceptibility factor. American journal of medical genetics. Part A. 2004 Nov ;131(1):11-7.

Jung Y-S, Qian Y, Chen X. Examination of the expanding pathways for the regulation of p21 expression and activity. Cellular signalling. 2010 Jul ;22(7):1003-12.

Kakinuma N, Zhu Y, Wang Y, Roy BC, Kiyama R. Kank proteins: structure, functions and diseases. Cellular and molecular life sciences : CMLS. 2009 Aug ;66(16):2651-9.

Kim S-J, Cox N, Courchesne R, Lord C, Corsello C, Akshoomoff N, et al. Transmission disequilibrium mapping at the serotonin transporter gene (SLC6A4) region in autistic disorder. Molecular psychiatry. 2002 Jan ;7(3):278-88.

Kumar RA, Christian SL. Genetics of autism spectrum disorders. Current Neurology and Neuroscience Reports. 2009 Apr ;9(3):188-197.

Lauritsen MB, Als TD, Dahl HA, Flint TJ, Wang AG, Vang M, et al. A genome-wide search for alleles and haplotypes associated with autism and related pervasive developmental disorders on the Faroe Islands. Molecular psychiatry. 2006 Jan ;11(1):37-46.

Lee SM, Tole S, Grove E, McMahon AP. A local Wnt-3a signal is required for development of the mammalian hippocampus. Development (Cambridge, England). 2000 Feb ;127(3):457-67.

Lerer E, Levi S, Salomon S, Darvasi A, Yirmiya N, Ebstein RP. Association between the oxytocin receptor (OXTR) gene and autism: relationship to Vineland Adaptive Behavior Scales and cognition. Molecular psychiatry. 2008 Oct ;13(10):980-8.

Li J, Tabor HK, Nguyen L, Gleason C, Lotspeich LJ, Spiker D, et al. Lack of association between HoxA1 and HoxB1 gene variants and autism in 110 multiplex families. American journal of medical genetics. 2002 Jan ;114(1):24-30.

Lukes a, Mun-Bryce S, Lukes M, Rosenberg G a. Extracellular matrix degradation by metalloproteinases and central nervous system diseases. Molecular neurobiology. 1999 Jun ;19(3):267-84.Available from: http://www.ncbi.nlm.nih.gov/pubmed/10495107

Luo W, Peterson A, Garcia BA, Coombs G, Kofahl B, Heinrich R, et al. Protein phosphatase 1 regulates assembly and function of the beta-catenin degradation complex. The EMBO journal. 2007 Mar ;26(6):1511-21.

Maaser K, Borlak J. A genome-wide expression analysis identifies a network of EpCAM-induced cell cycle regulators. British journal of cancer. 2008 Nov ;99(10):1635-43.

Manfredi JJ. The Mdm2-p53 relationship evolves: Mdm2 swings both ways as an oncogene and a tumor suppressor. Genes & development. 2010 Aug ;24(15):1580-9.

Martin I, Gauthier J, D'Amelio M, Védrine S, Vourc'h P, Rouleau GA, et al. Transmission disequilibrium study of an oligodendrocyte and myelin glycoprotein gene allele in 431 families with an autistic proband. Neuroscience research. 2007 Dec ;59(4):426-30.

Maussion G, Carayol J, Lepagnol-Bestel A-M, Tores F, Loe-Mie Y, Milbreta U, et al. Convergent evidence identifying MAP/microtubule affinity-regulating kinase 1 (MARK1) as a susceptibility gene for autism. Human molecular genetics. 2008 Aug ;17(16):2541-51.

McCauley JL, Li C, Jiang L, Olson LM, Crockett G, Gainer K, et al. Genome-wide and Ordered-Subset linkage analyses provide support for autism loci on 17q and 19p with evidence of phenotypic and interlocus genetic correlates. BMC medical genetics. 2005 Jan ;61.

Miller DT, Shen Y, Weiss LA, Korn J, Anselm I, Bridgemohan C, et al. Microdeletion/duplication at 15q13.2q13.3 among individuals with features of autism and other neuropsychiatric disorders. Journal of medical genetics. 2009 Apr ;46(4):242-8.

Miosge N, Holzhausen S, Zelent C, Sprysch P, Herken R. Nidogen-1 and nidogen-2 are found in basement membranes during human embryonic development. The Histochemical journal. 33(9-10):523-30.

Moessner R, Marshall CR, Sutcliffe JS, Skaug J, Pinto D, Vincent J, et al. Contribution of SHANK3 mutations to autism spectrum disorder. American journal of human genetics. 2007 Dec ;81(6):1289-97.

Molloy CA, Keddache M, Martin LJ. Evidence for linkage on 21q and 7q in a subset of autism characterized by developmental regression. Molecular psychiatry. 2005 Aug ;10(8):741-6.

Mühlethaler-Mottet A, Flahaut M, Bourloud KB, Nardou K, Coulon A, Liberman J, et al. Individual caspase-10 isoforms play distinct and opposing roles in the initiation of death receptor-mediated tumour cell apoptosis. Cell death & disease. 2011 Jan ;2(1):e125.

Nishimoto S, Nishida E. Fibroblast growth factor 13 is essential for neural differentiation in Xenopus early embryonic development. The Journal of biological chemistry. 2007 Aug ;282(33):24255-61.

Pagnamenta AT, Wing K, Sadighi Akha E, Knight SJL, Bölte S, Schmötzer G, et al. A 15q13.3 microdeletion segregating with autism. European journal of human genetics : EJHG. 2009 May ;17(5):687-92.

Philippe A, Guilloud-Bataille M, Martinez M, Gillberg C, Råstam M, Sponheim E, et al. Analysis of ten candidate genes in autism by association and linkage. American journal of medical genetics. 2002 Mar ;114(2):125-8.

Philippe A. Genome-wide scan for autism susceptibility genes. Paris Autism Research International Sibpair Study Human Molecular Genetics. 1999 May ;8(5):805-812.

Raiford KL, Shao Y, Allen IC, Martin ER, Menold MM, Wright HH, et al. No association between the APOE gene and autism. American journal of medical genetics. Part B, Neuropsychiatric genetics : the official publication of the International Society of Psychiatric Genetics. 2004 Feb ;125B(1):57-60.

Rauch U. Extracellular matrix components associated with remodeling processes in brain. Cellular and molecular life sciences : CMLS. 2004 Aug ;61(16):2031-45.

Rehnström K, Ylisaukko-oja T, Nieminen-von Wendt T, Sarenius S, Källman T, Kempas E, et al. Independent replication and initial fine mapping of 3p21-24 in Asperger syndrome. Journal of medical genetics. 2006 Feb ;43(2):e6.

Reyland ME. Protein kinase C isoforms: Multi-functional regulators of cell life and death. Frontiers in bioscience : a journal and virtual library. 2009 Jan ;142386-99.

Risch N, Spiker D, Lotspeich L, Nouri N, Hinds D, Hallmayer J, et al. A genomic screen of autism: evidence for a multilocus etiology. American journal of human genetics. 1999 Aug ;65(2):493-507.

Robinson PD, Schutz CK, Macciardi F, White BN, Holden JJ. Genetically determined low maternal serum dopamine beta-hydroxylase levels and the etiology of autism spectrum disorders. American journal of medical genetics. 2001 Apr ;100(1):30-6.

Ronald A, Butcher LM, Docherty S, Davis OSP, Schalkwyk LC, Craig IW, et al. A genome-wide association study of social and non-social autistic-like traits in the general population using pooled DNA, 500 K SNP microarrays and both community and diagnosed autism replication samples. Behavior genetics. 2010 Jan ;40(1):31-45.

Sancho-Martinez I, Martin-Villalba A. Tyrosine phosphorylation and CD95: a FAScinating switch. Cell cycle (Georgetown, Tex.). 2009 Mar ;8(6):838-42.

Sato K, Ohta T, Venkitaraman AR. A mitotic role for the DNA damage-responsive CHK2 kinase. Nature cell biology. 2010 May ;12(5):424-5.

Schier AF. Nodal signaling in vertebrate development. Annual review of cell and developmental biology. 2003 Jan ;19589-621.

Segurado R, Conroy J, Meally E, Fitzgerald M, Gill M, Gallagher L. Confirmation of association between autism and the mitochondrial aspartate/glutamate carrier SLC25A12 gene on chromosome 2q31. The American journal of psychiatry. 2005 Nov ;162(11):2182-4.

Serajee FJ, Zhong H, Mahbubul Huq AHM. Association of Reelin gene polymorphisms with autism. Genomics. 2006 Jan ;87(1):75-83.

Silverman JM, Buxbaum JD, Ramoz N, Schmeidler J, Reichenberg A, Hollander E, et al. Autism-related routines and rituals associated with a mitochondrial aspartate/glutamate carrier SLC25A12 polymorphism. American journal of medical genetics. Part B, Neuropsychiatric genetics : the official publication of the International Society of Psychiatric Genetics. 2008 Apr ;147(3):408-10.

Skaar DA, Shao Y, Haines JL, Stenger JE, Jaworski J, Martin ER, et al. Analysis of the RELN gene as a genetic risk factor for autism. Molecular psychiatry. 2005 Jun ;10(6):563-71.

Soundararajan R, Wang J, Melters D, Pearce D. Differential activities of glucocorticoid-induced leucine zipper protein isoforms. The Journal of biological chemistry. 2007 Dec ;282(50):36303-13.

Southwood C, Gow A. Molecular pathways of oligodendrocyte apoptosis revealed by mutations in the proteolipid protein gene. Microscopy research and technique. 2001 Mar ;52(6):700-8.

Stamenkovic I. Extracellular matrix remodelling: the role of matrix metalloproteinases. The Journal of pathology. 2003 Jul ;200(4):448-64.

Stork P. Crosstalk between cAMP and MAP kinase signaling in the regulation of cell proliferation Trends in Cell Biology. 2002 Jun ;12(6):258-266.

Stuart SW, King CH, Pai GS. Autism spectrum disorder, Klinefelter syndrome, and chromosome 3p21.31 duplication: a case report. MedGenMed : Medscape general medicine. 2007 Jan ;9(4):60.

Stubbs EG, Magenis RE. HLA and autism Journal of Autism and Developmental Disorders. 1980 Mar ;10(1):15-19.

Sutcliffe JS, Delahanty RJ, Prasad HC, McCauley JL, Han Q, Jiang L, et al. Allelic heterogeneity at the serotonin transporter locus (SLC6A4) confers susceptibility to autism and rigid-compulsive behaviors. American journal of human genetics. 2005 Aug ;77(2):265-79.

Swanberg SE, Nagarajan RP, Peddada S, Yasui DH, LaSalle JM. Reciprocal co-regulation of EGR2 and MECP2 is disrupted in Rett syndrome and autism. Human molecular genetics. 2009 Feb ;18(3):525-34.

Szatmari P, Paterson AD, Zwaigenbaum L, Roberts W, Brian J, Liu X-Q, et al. Mapping autism risk loci using genetic linkage and chromosomal rearrangements. Nature genetics. 2007 Mar ;39(3):319-28.

Takada T, Noguchi T, Inagaki K, Hosooka T, Fukunaga K, Yamao T, et al. Induction of apoptosis by stomach cancer-associated protein-tyrosine phosphatase-1. The Journal of biological chemistry. 2002 Sep ;277(37):34359-66.

Tanaka K, Nigg EA. Cloning and characterization of the murine Nek3 protein kinase, a novel member of the NIMA family of putative cell cycle regulators. The Journal of biological chemistry. 1999 May ;274(19):13491-7.

Taskén K, Aandahl EM. Localized effects of cAMP mediated by distinct routes of protein kinase A. Physiological reviews. 2004 Jan ;84(1):137-67.

Torres A. The transmission disequilibrium test suggests that HLA-DR4 and DR13 are linked to autism spectrum disorder Human Immunology. 2002 Apr ;63(4):311-316.

Torres AR, Sweeten TL, Cutler A, Bedke BJ, Fillmore M, Stubbs EG, et al. The association and linkage of the HLA-A2 class I allele with autism. Human immunology. 2006 ;67(4-5):346-51.

Toyo-oka K, Shionoya A, Gambello MJ, Cardoso C, Leventer R, Ward HL, et al. 14-3-3epsilon is important for neuronal migration by binding to NUDEL: a molecular explanation for Miller-Dieker syndrome. Nature genetics. 2003 Jul ;34(3):274-85.

Trikalinos TA, Karvouni A, Zintzaras E, Ylisaukko-oja T, Peltonen L, Järvelä I, et al. A heterogeneity-based genome search meta-analysis for autism-spectrum disorders. Molecular psychiatry. 2006 Jan ;11(1):29-36.

Trueb B, Taeschler S. Expression of FGFRL1, a novel fibroblast growth factor receptor, during embryonic development. International journal of molecular medicine. 2006 Apr ;17(4):617-20.

Vantaggiato C, Redaelli F, Falcone S, Perrotta C, Tonelli A, Bondioni S, et al. A novel CLN8 mutation in late-infantile-onset neuronal ceroid lipofuscinosis (LINCL) reveals aspects of CLN8 neurobiological function. Human mutation. 2009 Jul ;30(7):1104-16.

Wang L, Jia M, Yue W, Tang F, Qu M, Ruan Y, et al. Association of the ENGRAILED 2 (EN2) gene with autism in Chinese Han population. American journal of medical genetics. Part B, Neuropsychiatric genetics : the official publication of the International Society of Psychiatric Genetics. 2008 Jun ;147B(4):434-8.

Warren R. Strong association of the third hypervariable region of HLA-DRß1 with autism. Journal of Neuroimmunology. 1996 Jul ;67(2):97-102.

Wu S, Jia M, Ruan Y, Liu J, Guo Y, Shuang M, et al. Positive association of the oxytocin receptor gene (OXTR) with autism in the Chinese Han population. Biological psychiatry. 2005 Jul ;58(1):74-7.

Yang MS, Gill M. A review of gene linkage, association and expression studies in autism and an assessment of convergent evidence. International journal of developmental neuroscience : the official journal of the International Society for Developmental Neuroscience. 2007 Apr ;25(2):69-85.

Yirmiya N, Pilowsky T, Nemanov L, Arbelle S, Feinsilver T, Fried I, et al. Evidence for an association with the serotonin transporter promoter region polymorphism and autism. American journal of medical genetics. 2001 May ;105(4):381-6.

Ylisaukko-oja T, Alarcón M, Cantor RM, Auranen M, Vanhala R, Kempas E, et al. Search for autism loci by combined analysis of Autism Genetic Resource Exchange and Finnish families. Annals of neurology. 2006 Jan ;59(1):145-55.

Yu H. Cdc20: a WD40 activator for a cell cycle degradation machine. Molecular cell. 2007 Jul ;27(1):3-16.

Yu J, Lan J, Zhu Y, Li X, Lai X, Xue Y, et al. The E3 ubiquitin ligase HECTD3 regulates ubiquitination and degradation of Tara. Biochemical and biophysical research communications. 2008 Mar ;367(4):805-12.

Yu Y-M, Pace SM, Allen SR, Deng C-X, Hsu L-C. A PP1-binding motif present in BRCA1 plays a role in its DNA repair function. International journal of biological sciences. 2008 Jan ;4(6):352-61.

Zagris N. Extracellular matrix in development of the early embryo. Micron. 2000 Jun ;32(4):427-438.

Zákány J, Kmita M, Alarcon P, Pompa J-L de la, Duboule D. Localized and Transient Transcription of Hox Genes Suggests a Link between Patterning and the Segmentation Clock Cell. 2001 Jul ;106(2):207-217.

Zhou H, Yoshioka T, Nathans J. Retina-derived POU-domain factor-1: a complex POU-domain gene implicated in the development of retinal ganglion and amacrine cells. The Journal of neuroscience : the official journal of the Society for Neuroscience. 1996 Apr ;16(7):2261-74.
